# Mesoionic Imines (MIIs): Strong Donors and Versatile Ligands for Transition Metals and Main Group Substrates

**DOI:** 10.1002/anie.202200653

**Published:** 2022-04-19

**Authors:** Richard Rudolf, Nicolás I. Neuman, Robert R. M. Walter, Mark. R. Ringenberg, Biprajit Sarkar

**Affiliations:** ^1^ Lehrstuhl für Anorganische Koordinationschemie Institut für Anorganische Chemie University of Stuttgart Pfaffenwaldring 55 70569 Stuttgart Germany; ^2^ Instituto de Desarrollo Tecnológico para la Industria Química INTEC, UNL-CONICET Predio CONICET Santa Fe “Dr. Alberto Cassano” Colectora Ruta Nacional 168, Km 0 Paraje El Pozo S3000ZAA) Santa Fe Argentina

**Keywords:** C−H Activation, Electronic Ambivalence, Fluorine Specific Interactions, Mesoionic Imines, Strong Donors

## Abstract

We report the synthesis and the reactivity of 1,2,3‐triazolin‐5‐imine type mesoionic imines (MIIs). The MIIs are accessible by a base‐mediated cycloaddition between a substituted acetonitrile and an aromatic azide, methylation by established routes and subsequent deprotonation. C=O‐stretching frequencies in MII−CO_2_ and −Rh(CO)_2_Cl complexes were used to determine the overall donor strength. The MIIs are stronger donors than the N‐heterocyclic imines (NHIs). MIIs are excellent ligands for main group elements and transition metals in which they display substituent‐induced fluorine‐specific interactions and undergo C−H activation. DFT calculations gave insights into the frontier orbitals of the MIIs. The calculations predict a relatively small HOMO–LUMO gap compared to other related ligands. MIIs are potentially able to act as both π‐donor and π‐acceptor ligands. This report highlights the potential of MIIs to display exciting properties with a huge potential for future development.

## Graphical abstract

Experimental and computational data disclose an interesting view on the reactivity of mesoionic imines (MIIs), whose versatile synthesis and coordination behavior are reported herein. MIIs appear to be stronger donors compared to their NHI‐congeners and exhibit unique properties like fluorine specific interaction or C−H‐activation.

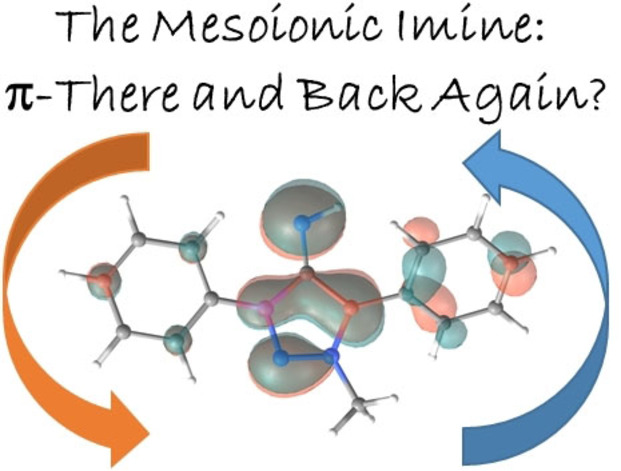

## Introduction

**Scheme 1:**
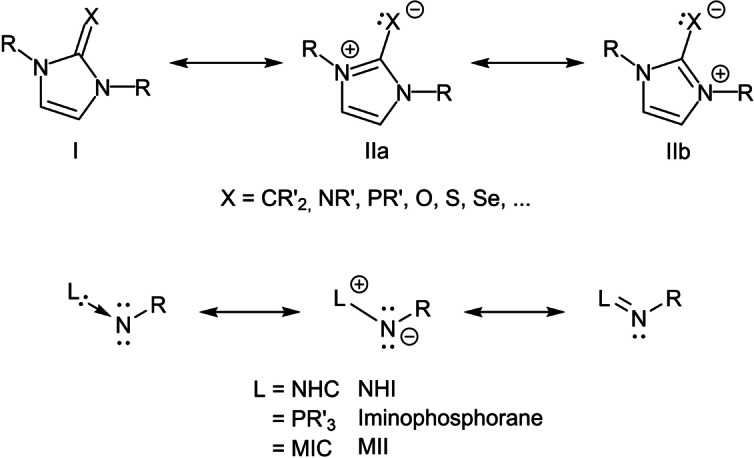
Top: Mesomeric structures of NHC‐main group element fragment X adducts. Bottom: Formal contemplation of the bonding situation in N‐heterocyclic imines and their analogues.

N‐heterocyclic carbenes (NHC) represent a group of compounds that are irreplaceable since their first isolation in 1991 by Arduengo et al.^[1]^ The variety of applications stem from homogeneous catalysis,^[2]^ material science^[3]^ and medicinal chemistry^[4]^ to name a few. Replacement of the free electron pair in NHCs with an organic moiety or atom X (X=CR′_2_, NR′, PR′, O, S, Se, …) results in compounds with novel properties and a reduced propensity towards back bonding due to a high electron population on the exocyclic fragment X.[^5^, ^6^, ^7^] Formulation of mesomeric structures (Scheme 1) shows that the exocyclic C−X bond is strongly polarised towards the exocyclic fragment. As the imidazolium moiety is highly capable of stabilising the resulting positive charge, the mesomeric structures IIa and IIb are stabilised, hence a high electron density is observed on the X‐fragment.

The resulting compounds for X=NR are generally called N‐heterocyclic imines (NHIs). Formally, the structure of NHIs can be formulated as an NHC‐stabilised nitrene (Scheme 1, bottom). According to this consideration, NHIs can be described as NHC‐analogues of iminophosphoranes and neutral amides. And indeed, NHIs show similar donor capacity as iminophosphoranes[^8^, ^9^] while they are better donors compared to NHCs^[10]^ or nitrogen‐based ligands like pyridines and imines.^[11]^ NHIs are therefore found to stabilise late transition metal complexes[^7^, ^12^] and main group element fragments.[^13^, ^14^] Mesoionic carbenes (MICs) of the 1,2,3‐triazol‐5‐ylidene type are a relatively new class of ligands that belong to the broader NHC family.[^15^, ^16^] Even though the use of the term MICs for these classes of compounds can be controversially discussed, there is indeed some justification for using the term MICs for such compounds.^[17]^ Their much stronger donor abilities compared to classical NHCs, and their relatively tuneable π‐acceptor properties,^[6]^ make them an exciting class of compounds in their own right in transition metal and main group chemistry.[^16^, ^18^] Recent prominent applications of MIC containing compounds have been, amongst others, in redox chemistry,[^19^, ^20^, ^21^] photochemistry,[^19^, ^21^, ^22^, ^23^] electrocatalysis,[^23^, ^24^] cooperative catalysis,^[25]^ and redox‐switchable catalysis.^[26]^ Considering the current interest in MIC ligands and their compounds, it is certainly worthwhile to look for access to compounds of the mesoionic olefin (MIOs) and mesoionic imine (MIIs) with a 1,2,3‐triazolium core. Both these types of compounds are likely to deliver exciting chemistry that will be comparable to, but also intriguing and often different from, their NHO and NHI congeners. That this can be expected is corroborated by looking at the chemistry of MICs vis a vis classical NHCs. In this regard, the synthesis, isolation and characterisation of MIOs and their compounds were recently reported.[^27^, ^28^]

Since the term “mesoionic” was defined in the mid twentieth century,^[29]^ the chemistry of MIIs has been a widely established field in heterocyclic chemistry with a focus on the synthesis and gauging of the reactivity.[^30^, ^31^] Many of the studied compounds were only proposed as intermediates in reactions^[32]^ or decomposed upon generation[^33^, ^34^, ^35^, ^36^] while stable MIIs were almost exclusively isolated with an electron‐withdrawing moiety on the exocyclic N (Scheme 1, bottom: R=EWG, L=heterocycles).[^34^, ^35^, ^36^, ^37^] The stable MIIs were found to act as N‐nucleophiles^[38]^ and react as 1,3‐dipoles in numerous cycloaddition reactions.^[39]^ The electronic properties go beyond the intriguing formulation of structures “on paper” as the mesoionic character strongly influences many of the investigated physical properties. In this regard, quantum chemical calculations,^[40]^ crystallographic data,^[40]^ heteronuclear NMR studies[^30^, ^41^] and intense colouring^[42]^ of the compounds point to the mesoionic property. Applications in coordination chemistry of MIIs besides the “classical” and non‐mesoionic NHI has been to the best of our knowledge not reported yet.

In this contribution, we report on the synthesis, isolation and characterization of MIIs. The focus of this work here is on the development of a modular synthetic route for MIIs of the 1,2,3‐triazolin‐3‐imine type, exploration of their donor properties, and the investigations of their reactivity towards transition metals and main group substrates, with an aim of establishing this new class of compounds in synthetic chemistry with potential applications.

## Results and Discussion

**Scheme 2:**
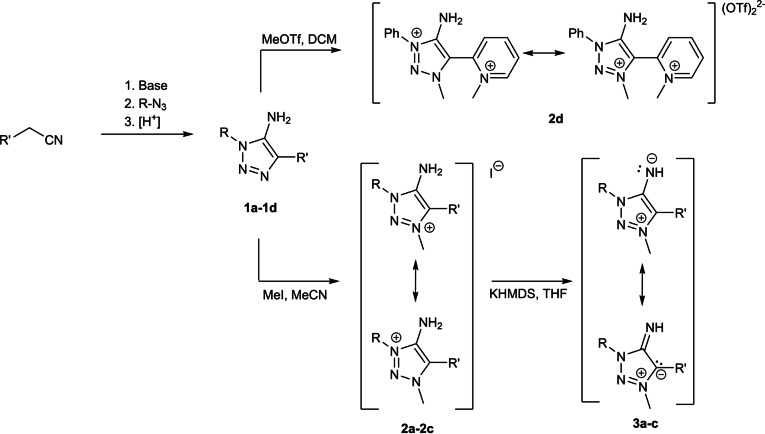
General synthetic route to MIIs and their precursors. a) R=R′=Ph; b) R=Mes, R′=Ph; c) R=R′=Mes; d) R=Ph, R′=Py.

The synthesis of the first MII with a 1,2,3‐triazolin core was reported recently by Haraguchi et al. by a fluoride mediated amination of 4‐chloro‐1,2,3‐triazolium salts followed by deprotonation.^[43]^ In parallel to this development, we identified 5‐amino‐1,2,3‐triazoles as versatile building block for MIIs, which we therefore aimed to construct. The 5‐amino‐1,2,3‐triazoles **1 a**–**d** were obtained based on a reported base‐mediated cycloaddition^[44]^ of a substituted acetonitrile derivative with an aromatic azide (Scheme 2).

**Scheme 3:**
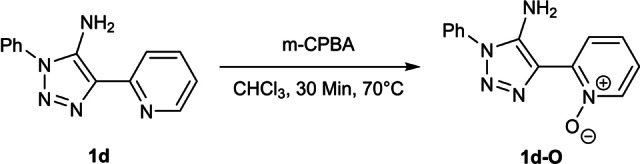
Generation of the N‐oxide from **1 d**.

Subsequent methylation with methyl iodide yielded the desired triazolium iodides **2 a**–**c** in satisfying to nearly quantitative yields. Methylation of **1 d** with MeI yielded a product mixture from which no products could be identified. We therefore conducted the methylation of **1 d** with MeOTf. The methylation of the C4‐pyridyl substituted triazole **1 d** with MeOTf was not selective towards either the triazole or the pyridyl moiety and a product mixture with the respective mono‐ and bismethylated triazolium salts was obtained. Only the bismethylated product **2 d** could be isolated (Scheme 2). Protection of the pyridiyl‐moiety via the respective N‐oxide with m‐CPBA^[45]^ prior to methylation yielded the desired product **1 d**‐**O** (Figure S69^[46]^) but isolation attempts of the pure form failed (Scheme 3).

**Figure 1:**
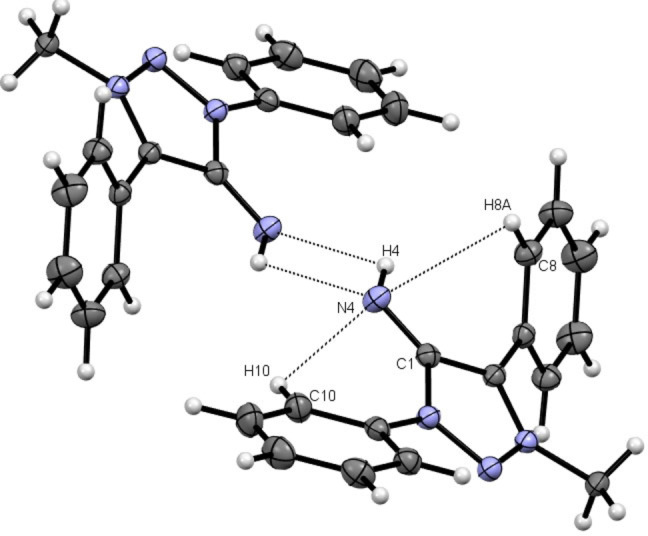
X‐ray solid‐state structure of **3 a**. Ellipsoids are all set at 50 % probability. Selected bond parameters in [Å] and [°]: C1−N4 1.296(2), N4··· H4 2.72(2), N4−H4··· N4 121.8(1), H4−N4··· H4 58.2(1), N4−H10 2.32(2), H10−N4−C1 94.8(4), N4−H8A 2.90(2), H8A−N4−C1 75.7(3).

**Figure 2:**
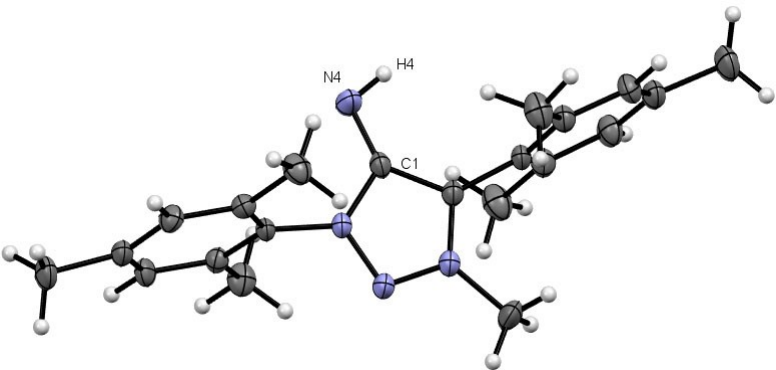
X‐ray solid‐state structure of **3 c**. Ellipsoids are all set at 50 % probability.^[46]^

The molecular structure of **3 a** ^[46]^ in the crystal shows a dimeric structure resulting from hydrogen‐bonding interactions from the exocyclic N‐fragment with the proton of another **3 a** molecule and *vice versa* incorporating a (N4−H4⋅⋅⋅N4′−H4′⋅⋅⋅)‐parallelogram (Figure 1). The solid state structure of **3 c** shows a monomeric molecular structure in the crystal^[46]^ with the mesityl moieties nearly ideally perpendicular to the triazole ring (Figure 2). The distinct C1−N4 bond length for **3 a** and **3 c** is in the range of reported bond length of NHIs.^[47]^ Beside intermolecular interactions, intramolecular interactions of N4 with the o−H atoms of the phenyl moieties (H10 and H8A) are apparent in the obtained molecular structure of **3 a**, which is further discussed in the Supporting Information. It is noticeable that the C1−N4 bond order significantly increases in the series of **1 a**–**3 a** as the C1−N4 bond significantly shortens in the series and the respective C1−N4 vibrational are blueshifted in the series (Table 1),^[46]^ which is also observed in the respective bismesityl substituted congeners **1**–**3 c**.

**Table 1:** Selected bond lengths and spectroscopic data for selected compounds^[46]^ and reported imine‐B(C_6_F_5_)_3_ adducts **6 a**–**c**.

Compound	*d*_C1−N4_ [Å]	*d*_N4−B1_[Å]	$\tilde{\nu }$_C1−N3_ [cm^−1^]^[a]^	*δ* ^1H^(N−*H*) [ppm]
**1 a**	1.362(2)	–	1598	4.12^[d]^ 4.65^**[**e]^
**1 b**	–	–	–	4.08^[d]^ 4.47^**[**e]^
**1 c**	1.359(4)		–	3.39^[d]^ 4.40^**[**e]^
**2 a**	1.353(3)	–	1613	5.07^[d]^ 5.47^**[**e]^
**2 b**	–	–	–	5.32^[d]^ 5.40^**[**e]^ 5.54^**[**e]^
**2 c**	1.363(7)	–	–	5.28^[d]^ 5.53^**[**e]^
**3 a**	1.296(2)	–	1719	4.78^**[**e]^ 5.49^**[**f]^
**3 c**	1.300(3)	–	–	2.33^**[**e]^ 3.48^**[**f]^
**3 a‐B(C_6_F_5_)_3_**	1.343(6)	1.581(7)	1646	4.93^**[**f]^
**6 a^[^** ^b]^	1.297(6)	1.642(8)	1646	–
**6 b^[^** ^b]^	1.293(2)	1.640(2)	1651	–
**6 c^[^** ^b]^	1.285(2)	1.627(3)	–^**[**c]^	–

[a] Powder. [b] ref. [48]. [c] No data from ref. [48]. [d] Recorded in CDCl_3_. [e] Recorded in CD_3_CN. [f] Recorded in C_6_D_6_.

**Scheme 4:**
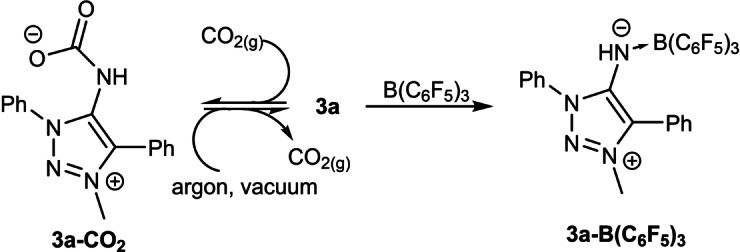
Reaction of **3 a** with CO_2_ B(C_6_F_5_)_3_.

Reaction of a solution of **3 a** with CO_2_ gas in non‐polar solvents yielded a colourless precipitate after several minutes (Scheme 4). According to ^1^H NMR ‐spectroscopy removal of the CO_2_ atmosphere resulted in CO_2_ loss and regeneration of the free ligand **3 a**. Indication for the reformation of the free ligand **3 a** was observed as the colourless solid was redissolved after removal of the CO_2_‐atmosphere and a bright yellow solution was obtained.

The apparently reversible CO_2_ fixation was further investigated by NMR‐ and IR‐spectroscopy. ^1^H NMR spectroscopic measurements after reaction of a solution of **3 a** with CO_2_ gas in deuterated acetonitrile show that a selective reaction occurs and a well‐defined product was obtained (Figure S42). ^13^C NMR spectroscopic measurements lead to the same result (Figure S43). Flushing the solution with argon afforded the ^1^H NMR ‐spectrum of the free ligand **3 a** again, confirming the reversibility of the CO_2_ fixation by **3 a**. IR‐experiments show a strong band rising at ∼1613 cm^−1^ (Figure S44). This characteristic band can be assigned to the symmetrical C=O stretching frequency of **3 a**‐**CO_2_** as analogous compounds show similar IR‐spectra.^[14]^ The C=O stretching frequency of **3 a**‐**CO_2_** is found to be red shifted compared to imidazolin‐2‐imine carboxylates which were reported to be in the range of 1670–1650 cm^−1^ for different substituted imines.^[14]^ This behaviour would follow the general trend of triazolylidene based ligands being stronger donors compared to imidazolylidene based ligands. As the N‐CO_2_ bond of the adduct breaks easily after exposure to either argon or ambient conditions, attempts to isolate **3 a**‐**CO_2_** failed.

**Figure 3:**
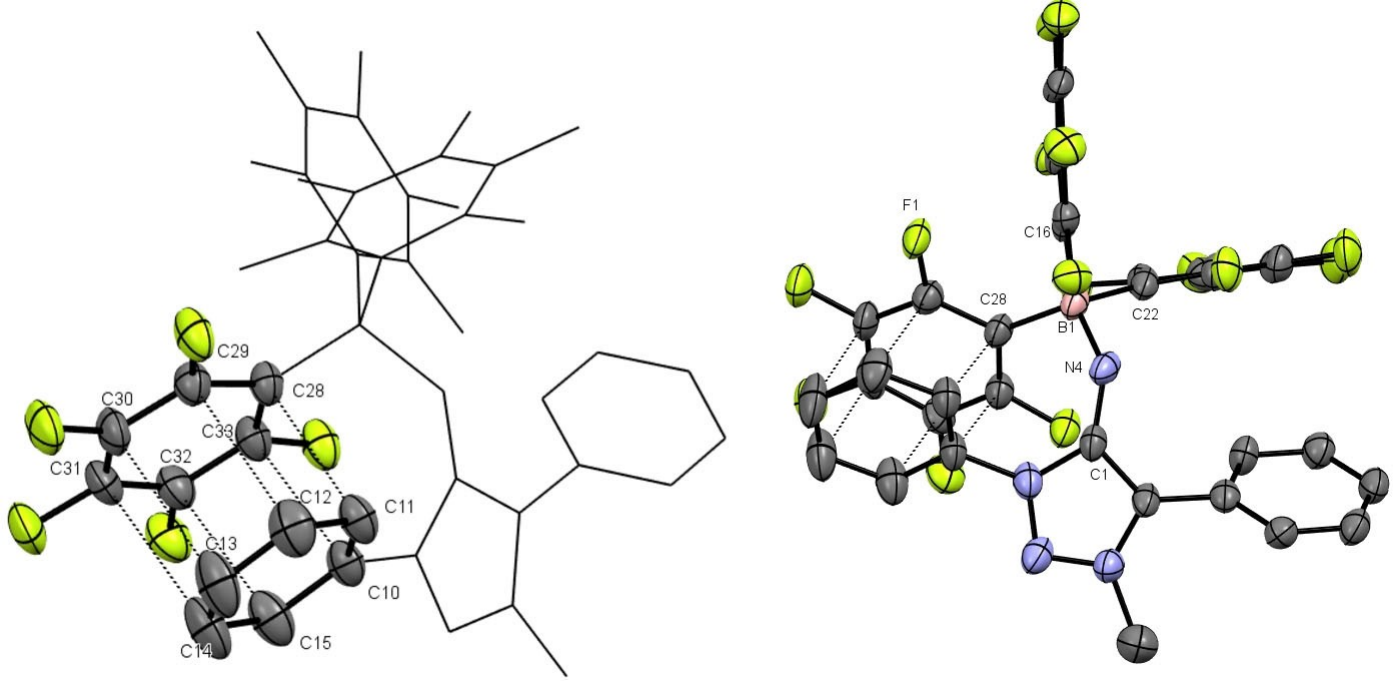
X‐ray solid‐state structure of **3 a‐B(C_6_F_5_)_3_**. Ellipsoids are all set at 50 % probability. Hydrogen‐atoms were omitted for clarity. Selected bond parameters in [Å] and [°]: C1−N4 1.343(6), N4−B1 1.581(7); C1‐N4‐B1 132.8(4), N4‐B1‐C22 105.6(3), N4‐B1‐C16 110.3(4), N4‐B1‐C22 109.4(4), C16‐B1‐C22 104.6(4), C16‐B1‐C28 104.6(4), C22‐B1‐C28 112.2(4) C10−C33 3.468(7), C11−C28 3.606(7), C12−C29 3.812(7), C13−C30 3.914(9), C14−C31 3.798(9), C15−C32 3.545(8). Left: Selected atoms were omitted to highlight π(C_6_F_5_)‐π(C_6_H_5_)‐interactions.

The reaction of B(C_6_F_5_)_3_ with **3 a** yielded the borane complex **3 a**‐**B(C_6_F_5_)_3_** (Scheme 4). The complex was characterised by spectroscopic methods and by single crystal X‐ray diffraction.^[46]^ Examination of the molecular structure in the crystal shows, that **3 a** coordinates via the exocyclic N‐fragment as expected (Figure 3). Upon coordination, the C1−N4 bond is elongated but still shorter than if the BR_3_‐fragment is formally exchanged with a proton (**2 a**) (Table 1). This behaviour was validated as the respective symmetrical vibrational frequencies of the C1−N4 bond is red shifted in moving from **3 a** to **3 a**‐**B(C_6_F_5_)_3_** to **2 a**. It is apparent that the C1−N4 is best characterised as intermediate between a double and single bond.

The C1−N4 bond length in the analogous, acyclic, imine‐B(C_6_F_5_)_3_ complexes **6 a**–**c** were found to be in the same range as in **3 a** (Figure S50 and Table 1).^[48]^ **3 a**‐**B(C_6_F_5_)_3_** does not follow this trend as the C1−N4 bond is much longer in **3 a**‐**B(C_6_F_5_)_3_** compared to **6 a**–**c**. On the other hand, the N4−B1 bond in **3 a**‐**B(C_6_F_5_)_3_** is significantly shorter compared to the acyclic derivatives (Table 1). The boron center in **3 a**‐**B(C_6_F_5_)_3_** shows a nearly ideal tetrahedral coordination geometry. The N4−B1 bond length with 1.58(1) Å is between reported N−B bond lengths of model compounds with purely dative (*d*_N−B_≈1.76 Å) or purely covalent (*d*_N−B_≈1.49 Å) bonds.^[49]^ Considerable N4‐B1 covalence in **3 a**‐**B(C6F5)** should therefore be assigned, which is supported by DFT calculations described below. Deviation from ideal tetrahedral bonding angles are attributed to π(C_6_F_5_)‐π(C_6_H_5_)‐interactions.

We conclude that **3 a** acts as a strong N‐donor by shifting electron density of the heterocyclic backbone onto the exocyclic N‐fragment, yielding an electron‐rich donor.

**Scheme 5:**
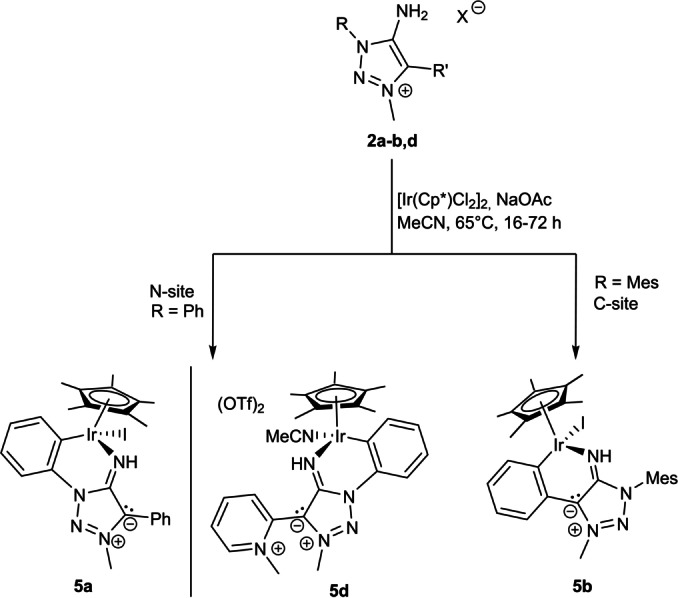
Regioselectivity of the ligands **2 a**, **2 b** and **2 d** towards C−H‐activation with [IrCp*Cl_2_]_2_.

To check the viability of the MIIs as ligands for transition metals we employed both the reactions of the corresponding amino‐triazolium salts with a suitable metal fragment either in the presence of a base or the direct reaction of isolated MIIs with metal fragments. The reaction of **2 a** with [Ir(Cp*)Cl_2_]_2_ under basic conditions (Scheme 5) resulted in a cyclometallated MII complex with the C−H activation taking place exclusively at the phenyl ring of the N1‐substituent. This observation is consistent with previous results on related 1,2,3‐triazol‐5‐ylidene based complexes.[^50^, ^51^] A similar reaction with **2 b** resulted in exclusive C−H activation at the phenyl ring of the C4‐substituent (Scheme 5). This is to be expected as the N1‐atom in **2 b** has a mesityl substituent.^[52]^ Surprisingly, the reaction of [Ir(Cp*)Cl_2_]_2_ with **2 d** under basic conditions (NaOAc) selectively yielded the cyclometallated product **5 d** via C−H‐activation of the N1‐phenyl substituent according to the obtained molecular structure and ^1^H NMR ‐spectroscopy. Interestingly, **5 a** was obtained in higher yields (75 %) than **5 b** (40 %). The observed intramolecular hydrogen bonding in **3 a** (Figure 1) implies that the hydrogen‐bonding of the exocyclic N‐fragment with the *ortho*‐protons of the N‐bound phenyl moiety is the driving force of the observed regioselectivity.

**Figure 4:**
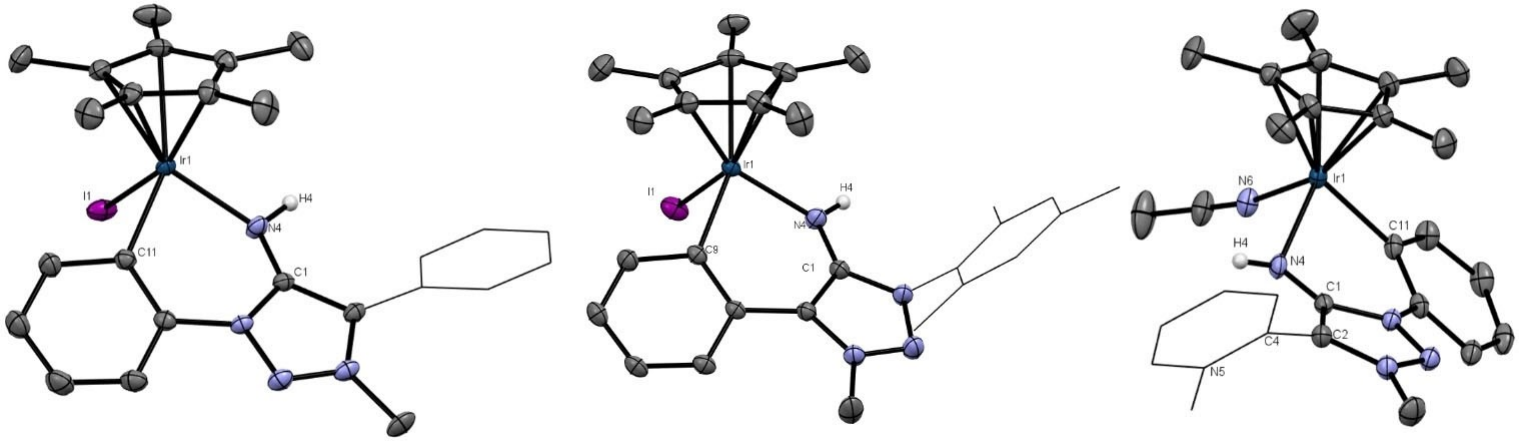
X‐ray solid‐state structure of MII‐IrCp*‐complexes. Ellipsoids are all set at 50 % probability. Selected Hydrogen‐atoms were omitted for clarity. Selected bond parameters in [Å] and [°]. Left: **5 a**: C1−N4 1.312(3), C1−N4−Ir1 123.6(2), N4−Ir1 2.069(2), I1−Ir1 2.711(1), C11−Ir1 2.053(2), Cp−Ir1 1.820(1); N4‐Ir1‐C11 86.9(1), C11‐Ir1‐I1 87.7(1), N4‐Ir1‐I1 89.6(1), Cp‐Ir1‐N4 126.2(1), Cp‐Ir1‐C11 130.1(1), Cp‐Ir1‐I1 123.6(1). Middle: **5 b**: C1−N4 1.307(3), C1−N4−Ir1 123.1(2), N4−Ir1 2.055(2), I1−Ir1 2.702(1), C9−Ir1 2.062(3), Cp−Ir1 1.820(1); N4‐Ir1‐C9 88.3(1), C9‐Ir1‐I1 88.0(1), N4‐Ir1‐I1 87.8(1), Cp‐Ir1‐N4 127.2(1), Cp‐Ir1‐C9 128.6(1), Cp‐Ir1‐I1 128.6(1). Co‐crystallised solvent (CH_2_Cl_2_) was omitted. Right: **5 d**: C1−N4 1.322(3); C1‐N4‐Ir1 117.4(2), N4−Ir1 2.096(2), N6−Ir1 2.045(2), C11−Ir1 2.062(3), Cp−Ir1 1.808(1); N4‐Ir1‐C11 84.9(1), C11‐Ir1‐N6 91.9(1), N4‐Ir1‐N6 86.2(1), Cp‐Ir1‐N4 127.1(1), Cp‐Ir1‐C11 126.7(1), Cp‐Ir1‐N6 126.7(1), N5‐C4‐C2‐C1 71.3(3), N5‐C4‐C2‐N3 −115.8(3). Counter‐anions were omitted for clarity.^[46]^

The molecular structures of the obtained iridium complexes **5 a**, **5 b** and **5 d** show that the iridium atom is coordinated in a highly distorted piano stool fashion (Figure 4).^[46]^ For all three complexes, most structural parameters are in the same range (Table S1). The C1−N4 bond is elongated in the row **5 a**–**5 d**. All three values are in between those of the free ligand **3 a** and the triazolium salt **2 a** (Table 1). The C1−N4 bond in all complexes is therefore best characterised as intermediate between an imine and an amine C−N bond like already presented for **3 a**‐**B(C_6_F_5_)_3_**. While in **5 a** and **5 b** a more iminic‐bond is observed, the smaller C1−N4−Ir1 angle and the significantly longer C1−N4 bond length in **5 d** suggest a more pronounced amidic bonding mode in **5 d**.

**Figure 5:**
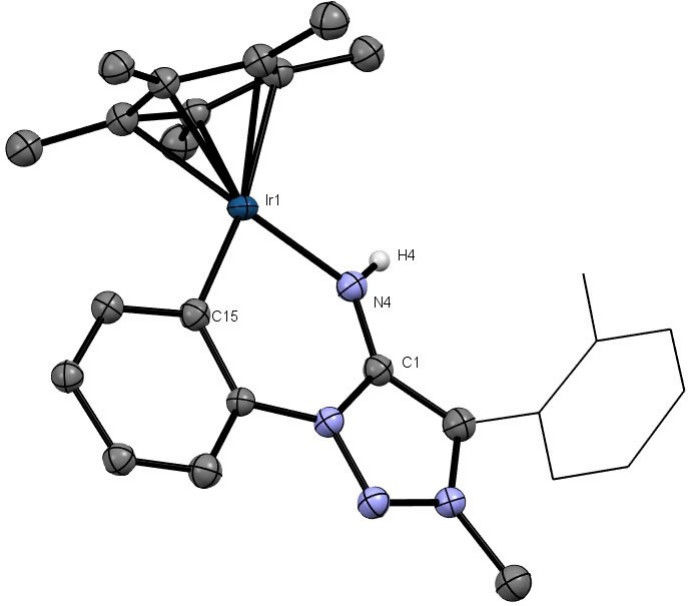
X‐ray solid‐state structure of **5 d‐acetone**. Ellipsoids are all set at 50 % probability. Selected Hydrogen‐atoms were omitted for clarity.^[46]^

**Scheme 6:**
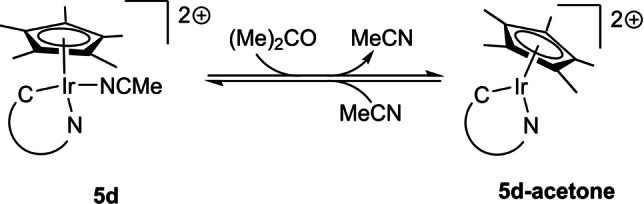
Reversible MeCN coordination and release of **5 d‐acetone**.

The auxiliary ligand MeCN in **5 d** can be removed by dissolving **5** in acetone according to CHN‐analysis and single‐crystal X‐ray diffractometry (Figure 5).^[46]^ This dissociation results in a coordinatively unsaturated Ir^III^ complex **5 d‐acetone** (Scheme 6). This transformation is reversible, and the stability of 5d‐acetone with a coordinatively unsaturated Ir^III^ center points to the strong π‐donor ability of the MII ligand.

The reaction of **3 a** with [Ir(COD)Cl]_2_ (Scheme S53) yielded a product mixture according to ^1^H NMR ‐spectroscopy (Figure S50) from which no product could be unequivocally determined. High‐resolution mass spectrometry did provide evidence for the formation of a MII−Ir(COD)Cl complex. The expected complex **3 a‐Ir(COD)Cl** was detected as either [*M*−H]^+^ (Figure S51) or [*M*−(HCl)−H]^+^ (Figure S52). Storage of this mixture in deuterated benzene overnight at room temperature yielded single crystals suitable for X‐ray diffractometry, revealing a cyclometallated species **4** (Figure S71) coordinated in a bidentate fashion by the imine ligand like in **5 a**.^[46]^ Cyclometallation reactions with Ir‐triazolylidene complexes bearing a N1‐substituted phenyl‐moiety are known reactivities and it is generally assumed that the cyclometallation is an equilibrium between a cyclometallated and a monodentate species.^[50]^

**Scheme 7:**
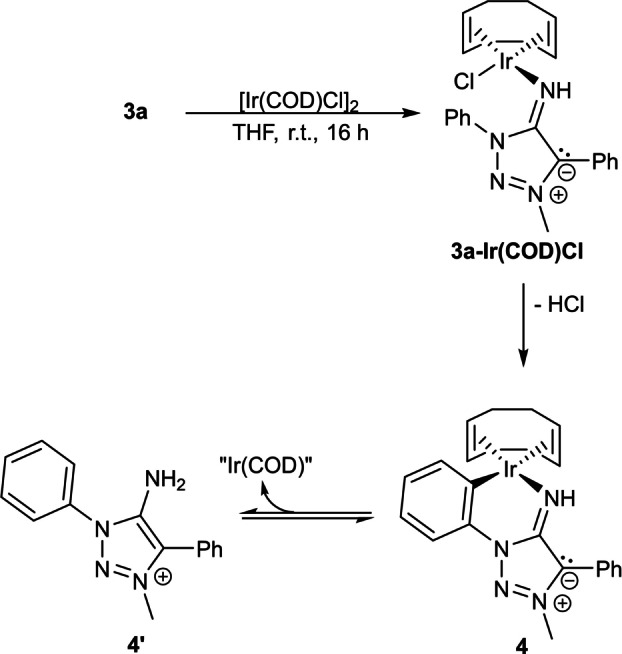
Expected reactivity of **3 a** towards [Ir(COD)Cl]_2_ and proposed further reactivity of **3 a‐Ir(COD)Cl**.

We therefore investigated whether analogous behaviour is observed in the system of **3 a** and [Ir(COD)Cl]_2_. The addition of either triethylamine or an ethereal HCl‐solution resulted in the selective formation of one species respectively: Addition of triethylamine into the aforementioned product mixture in CD_2_Cl_2_ resulted in the disappearance of one set of signals compared to the native ^1^H NMR ‐spectrum (Figure S53). The aromatic region of the resulting solution showed four distinct pyridine‐type signals and one phenyl‐moiety suggesting the formation of the cyclometallated species **4**. The addition of an ethereal HCl‐solution into the product mixture in CD_2_Cl_2_ on the other hand yielded aromatic signals of two inequivalent phenyl moieties comparable to the aromatic signals in the ^1^H NMR‐spectrum of **2 a** (Figure S10). The suspicion of a triazolium‐type species was further confirmed by the magnitude of the signals arising from the exocyclic protons. The integral shows that the exocyclic protons of this species have the magnitude of 2 relative to the N‐C*H*_3_‐moiety. Interestingly, the obtained product after HCl‐addition is not equivalent to the triazolium chloride **2 a(Cl)**, as we initially thought (Figure S57). Temperature dependent ^1^H NMR‐measurements show that the concentration of the triazolium salt **4′** increases upon cooling while the concentration of **4** decreases in a linear fashion (Figure S54, S55 and S56), thus confirming the presence of an equilibrium and a reversible HCl‐addition/abstraction. Through *van't Hoff* analysis, thermodynamic parameters of this equilibrium were determined (Table S3). As the entropy change was found to be positive, a dissociative mechanism (Scheme 7) is proposed.

**Scheme 8:**
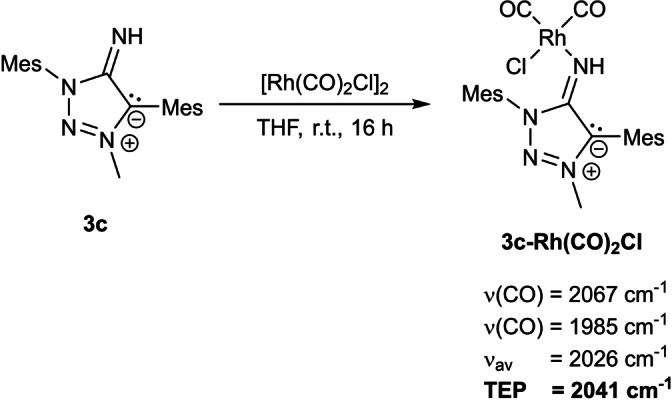
Reaction of **3 c** with [Rh(CO)_2_Cl]_2_ and characteristic CO‐bands of **3 c‐Rh(CO)_2_Cl**.

We conclude that the cyclometallation is the result of a preactivation of corresponding phenyl‐coordination site by ligand‐centred effects in **3 a**. We therefore used **3 c** as the ligand of choice to determine the TEP (*Tollmann‐Electronic‐Parameter*) of the MII ligands. By the addition of [Rh(CO)_2_Cl]_2_ into a solution of **3 c** in THF at room temperature the desired complex **3 c‐Rh(CO)_2_Cl** was obtained (Scheme 8). The complex was isolated in a pure form according to CHN‐analysis and ^1^H NMR ‐spectroscopy (Figure S35) after filtration of the residue in diethylether over a pad of cellite. The identity of the product and the connectivity within the molecule was confirmed by single‐crystal X‐ray diffractometry of a single crystal (Figure S72) of poor quality besides CHN‐analysis, NMR‐technqiues, IR‐spectroscopy and ESI‐MS. The poor data quality was found to be unsuitable for discussion of bond lengths in the molecular structure.

The resulting IR‐spectrum of **3 c‐Rh(CO)_2_Cl** shows three characteristic bands at 1597 cm^−1^ (*ν*(C‐N_exo_)), 1985 cm^−1^ and 2067 cm^−1^ (Figure S40). The latter two are assigned to the respective C=O‐stretching frequencies, from which the TEP‐values were determined.^[53]^ The obtained TEP indicates that MIIs are stronger donors than the respective benzimidazole based NHI,^[8]^ analogous neutral amides^[54]^ and generally MICs^[55]^ while mesoionic olefins are better donors than MIIs.^[27]^

**Figure 6:**
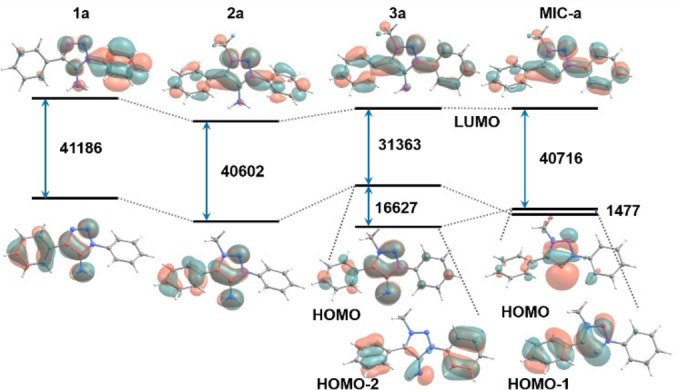
Energy levels, HOMO–LUMO gaps (in cm^−1^) and frontier orbitals, including the HOMO and LUMO, isosurfaces (isovalue=0.03) for **1 a**, **2 a**, **3 a** and **MIC‐a**. For **3 a** and **MIC‐a** the HO orbitals of σ and π symmetry are shown.

**Figure 7:**
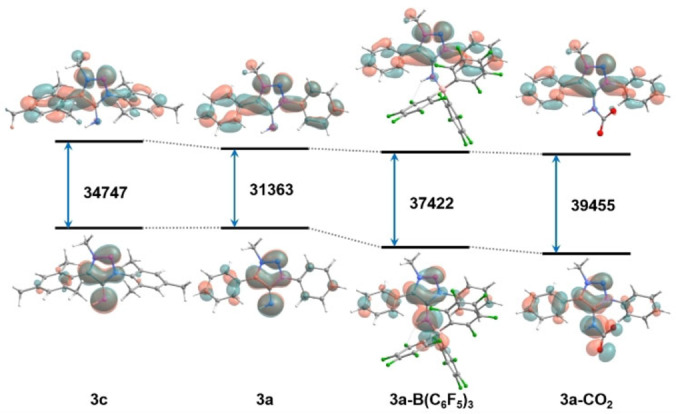
Energy levels, HOMO–LUMO gaps (in cm^−1^) and HOMO and LUMO orbital isosurfaces (isovalue=0.03) for **3 c**, **3 a**, **3 a**‐**B(C_6_F_5_)_3_** and **3 a‐CO_2_**.

Density Functional Theory calculations at the PBE0/def2‐TZVP/SMD(CH_2_Cl_2_) level, performed with Orca, version 4.2.1,^[56]^ were employed to analyse the electronic characteristics of some of the reported compounds. Figures 6 and 7 show the frontier orbitals (HOMO and LUMO) of compounds **1 a**–**3 a** and **MIC‐a**, and **3 c**, **3 a**, **3 a‐B(C_6_F_5_)_3_** and **3 a‐CO_2_**, respectively, as well as the HOMO–LUMO gaps in cm^−1^. For **3 a** and **MIC‐a**, the HOMO‐2 and HOMO‐1 orbitals, respectively, are shown. The HOMO orbitals in all cases except **MIC‐a**, have a noticeable π‐character on the NH_*x*_ moiety (*x*=1, 2). The HOMO and HOMO‐1 orbitals of **MIC‐a** have σ and π symmetry, respectively, while the opposite is the case for the HOMO and HOMO‐2 orbitals of **3 a**. In both cases this indicates that these ligands can act as σ‐ and π‐donors. In all cases it can be observed that the LUMO is centered on the (Ar)_2_MIC moiety, indicating that these compounds could also potentially act as π‐acceptors. In particular, **3 a** has a lower HOMO–LUMO gap compared to **MIC‐a**, suggesting the former to be a better π‐acceptor than the analogous carbene. Complexation of **3 a** with B(C_6_F_5_)_3_ and CO_2_ slightly perturbs the HOMO orbital, with delocalisation into the Lewis acid fragment, while it does not affect the LUMO orbital, which remains (Ar)_2_MIC‐centered. This suggests that in both cases there is a π‐donor effect but not a π‐acceptor one. This is reasonable given the strongly electron‐withdrawing nature of the C_6_F_5_ groups and of the carbonyl atoms in CO_2_. Additionally, the fact that the Ir^III^ complexes tolerate cyclometallated carbanionic donors despite the presence of the strongly donating MIIs, likely also indirectly points to the operation of additional π‐acceptor character of the MIIs. In order to unequivocally test if **3 a** could act as π‐acceptor, complexes with relatively electron‐rich metal centers should be studied.

**Figure 8:**
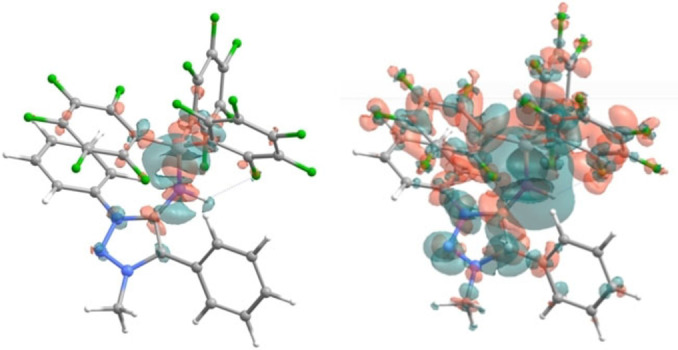
Electron density difference isosurfaces, obtained by subtracting the electron density of the fragments **3 a** and B(C_6_F_5_)_3_ from that of the complex **3 a**‐**B(C_6_F_5_)_3_**. Red indicates a region of positive (excess), and green one of negative (defect) electron density. Left: isovalue=0.005; right: isovalue=0.001.

More insight into the bonding between **3 a** and B(C_6_F_5_)_3_ can be obtained by plotting the electron density difference between the **3 a**‐**B(C_6_F_5_)_3_** complex and its composing parts.^[57]^ This electron density difference plot is shown in Figure 8, where red indicates positive (excess) and green indicates negative (defect) electron density.

The plots indicate that upon complexation, electron density from the MIC moiety is delocalized into the B(C_6_F_5_)_3_ moiety, particularly the C_6_F_5_ carbon π‐orbitals. This is congruent with a π‐donor behaviour of **3 a**, and with the strong electron‐withdrawing character of the C_6_F_5_ fragments. Additionally, the electron density excess (red) in the region between the N and B atoms shows the covalent bond of σ character, as earlier suggested by the N−B bond lengths.

## Conclusion

In summary, we have established a general synthetic route for MIIs. First reactivity tests towards complexation with main group substrates and transition metals have shown that these MII based ligands exceed their imidazolylidene derived NHI counterparts in donor strength. The reactivity of the MIIs with main group substrates displayed their potential in CO_2_ activation, as well as their ability to induce fluorine‐specific interactions. Their reactions with late transition metals showed their ability to induce C−H activation, and their propensity to stabilize coordinatively unsaturated Ir complexes. These are also very rare examples of the coordination of any NHI (in our specific case the MIIs) to late transition metals, and to the best of our knowledge, the only examples with such imines in which the ligands additionally undergo C−H activation. DFT calculations predict a small HOMO–LUMO gap, and the ability of the MIIs to act as both π‐donors as well as π‐acceptors. These ligands are thus potentially electronically ambivalent, and should be able to adjust electron densities at the coordination partner on demand. In view of the modular synthetic route reported for the access of these MIIs, and their rich chemistry towards both main group substrates and transition metals, we expect this new ligand class to play a major role in several branches of chemistry in the near future.

## Conflict of interest

The authors declare no conflict of interest.

1

## Supporting information

As a service to our authors and readers, this journal provides supporting information supplied by the authors. Such materials are peer reviewed and may be re‐organized for online delivery, but are not copy‐edited or typeset. Technical support issues arising from supporting information (other than missing files) should be addressed to the authors.

Supporting Information

## Data Availability

The data that support the findings of this study are available in the Supporting Information of this article.
