# Supplementary material for: Mesoionic Imines (MIIs): Strong Donors and Versatile Ligands for Transition Metals and Main Group Substrates
Source: Angew Chem Int Ed Engl. 2022 Apr 19;61(25):e202200653. doi: 10.1002/anie.202200653 (PMC9322014; doi:10.1002/anie.202200653)
Supplement: Supplementary file 1 — Supporting Information [file ANIE-61-0-s001.pdf]

Angewandte Chemie
Eine Zeitschrift der Gesellschaft Deutscher Chemiker

Supporting Information

# Mesoionic Imines (MIIs): Strong Donors and Versatile Ligands for Transition Metals and Main Group Substrates

*R. Rudolf, N. I. Neuman, R. R. M. Walter, M. R. Ringenberg, B. Sarkar**

**Table of Contents**

1. General Remarks .......... 3

2. Synthesis .......... 3

3. NMR-spectra .......... 11

4. IR-Spectra .......... 30

5. Detailed discussion of the crystallographic and spectroscopic data of **1-3** .......... 32

6. Complexation reactions .......... 33

6.1 Preparation of **3a-$CO_2$** solutions .......... 33

6.2 Preparation of **3a-$B(C_6F_5)_3$** .......... 35

6.3 Crystallographic data of Ir half sandwich complexes .......... 39

6.4 Preparation of **4**/**4’** solutions .......... 39

6.5 Preparation of **2a(Cl)** .......... 45

7. Computational details .......... 46

8. Crystallographic data .......... 58

References .......... 71

1. General Remarks

Manipulations were carried out using Schlenk-line techniques under the inert atmosphere of argon (Air liquide, ALPHAGAZTM 1, purity ≥ 99.999%). Phenylazide[1], Mesitylazide[2], **1a**[3] and **1d**[4] were prepared using published methods. Commercially available chemicals were used without further purification. Dry solvents were taken from solvent systems (GS GLOVEBOX or Innovative Technology PURESOLV) and degassed by ultra-sonication before use. NMR-spectra were recorded on either an *Avance 250*, *Avance 400*, *Avance 500* or *Avance 700* from Bruker. Chemical shifts in $^{1}$H-NMR-spectra are reported in ppm with reference to the residual solvents peaks ((δ($CHCl_3$) = 7.26 ppm, δ($C_6D_5H$) = 7.0 ppm, δ($HCDCl_2$) = 5.32 ppm, δ(($HD_2C)_2CO$) = 2.05 ppm, δ($HD_2CCN$) = 1.94 ppm)) relative to $Me_4Si$.[5] Heteronuclear NMR-spectra were calibrated using the Ξ-method[6]: $^{11}$B-NMR: 80 MHz/128 MHz, external standard: $BF_3{\cdot}OEt_2$, δ = 0.0 ppm. $^{13}$C-NMR: 63 MHz/101 MHz/126 MHz/ 176 MHz, external standard: $Me_4Si$, δ = 0.0 ppm. $^{19}$F-NMR: 376 MHz, external standard: $CFCl_3$, δ = 0.0 ppm. Coupling constants are given in Hz. Multiplets are denoted as follows: singlet (s), duplet (d), triplet (t), quartet (q), not-resolved multiplet (m), broad signal (br.) and combinations thereof. IR-spectrum were recorded on a *Nicolet iS5* from Thermo Fisher Scientific with an iD5 ATR-unit or with a liquid-cuvette ($CaF_2$-windows). Wavenumbers $\tilde{\nu}$ are reported in $cm^{-1}$. Mass spectrometry was performed on a *microTOFQ Bruker Daltonics*. Elemental analysis was performed on an *Elementar VarioMICRO* cube. X-ray diffractometry was carried out on either an *Apex II Duo* from Bruker with or a *STADIVARI* from STOE with molybdenum radiation ($\lambda_{K\alpha}$ = 0.71073 Å). Structures were solved by using SHELXL and refined using SHELXT.[7]

2. Synthesis

Synthesis of **1b**

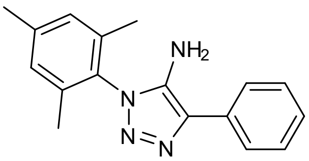


**1b**

Potassium butoxide (0.56 g, 5.02 mmol, 1.2 eq) and copper(I)-iodide were dissolved in DMSO (9 mL). The reaction vessel was placed into an ice-water bath and benzyl cyanide (0.49 g, 4.18 mmol, 1 eq) was added immediately dropwise. After complete addition 2,4,6-trimethylphenylazide (0.81 g, 5.02 mmol, 1.2 eq) was added dropwise into the solution. The solution was stirred for another 15 minutes after complete addition while still submerged in icy water. The solution was allowed to warm up to room temperature and stirring was continued for 2 hours. The reaction was quenched by the addition of icy water (100 mL), while the product precipitated as an orange solid. The solid was filtered off and washed with generous amounts of water (3x 100 mL). The solid was dissolved in $CH_2Cl_2$ (20 mL) and washed with aqueous $NH_4OH$-EDTA solution until the aqueous layer was colourless. The combined organic layers were dried ($Na_2SO_4$) and filtrated. The solvents were removed under reduced pressure yielding an orange oil. The crude product was dissolved in $Et_2O$ (5 mL). The addition of hexane resulted in the deposition of the product as a red oil. Decantation of the liquid and removing of volatiles on high vacuum yielded the desired product as an orange solid, which was used without further purification (1.69 mmol, 48%, 559 mg).

**$^{1}$H-NMR** (500 MHz, $CDCl_3$) δ: 7.79 (d, *J* = 7.27 Hz, Phenyl-o-*H*, 2H), 7.46 (t, *J* = 7.76 Hz, Phenyl-m-*H*, 2H), 7.29 (d, *J* = 7.36 Hz, Phenyl-p-*H*, 1H), 7.03 (s, Aryl-*H*, 2H), 3.85 (s, Trz-N*H*$_2$, 2H), 2.36 (s, Aryl-p-C*H*$_3$, 3H), 2.04 (s, Aryl-o-C*H*$_3$, 6H).

**$^{13}$C-NMR** (126 MHz, $CDCl_3$) δ: 140.7 (Aryl-o-*C*), 138.0, 136.6 (Aryl-p-*C*), 132.0, 129.7, 129.5 (Aryl-m-*C*), 129.0 (Phenyl-m-*C*), 128.6 (Phenyl-i-*C*), 126.7 (Phenyl-p-*C*), 125.2 (Phenyl-o-*C*), 21.2 (Aryl-p-*C*H$_3$), 17.4 (Aryl-o-*C*H$_3$).

**EA:** calc. ($C_{17}H_{18}N_4$, 278.36 g/mol) C 73.35, H 6.52, N 20.13; found C 72.95, H 6.61, N 19.21.

**MS (ESI):** m/z = 279.16 $[M+H]^+$, 301.14 $[M+Na]^+$.

**HRMS (ESI):** calc. ($C_{17}H_{18}N_4$) m/z = 279.1604 $[M+H]^+$, found m/z = 279.1604 $[M+H]^+$.

Synthesis of **1c**

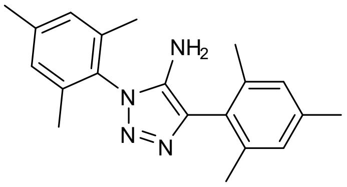

**1c**

2,4,6-Trimethylphenylacetonitrile (1.05 g, 6.59 mmol, 1 eq) was submitted into an oven dried Schlenk flask. THF (50 mL) was added under a flow of argon and the solution was cooled to -40 °C. A solution of *n*-butyllithium in hexanes (2.64 mL, 2.5 M, 1 eq) was added dropwise to the stirring solution and the solution was stirred for 10 minutes. 2,4,6-Trimethylphenylazide was added dropwise into the solution and the solution was over night while slowly heating up to room temperature. The solution was slowly poured into icy water (150 mL) and stirred for another 30 minutes at room temperature until all ice melted. The solution was extracted with EtOAc (3x 100 mL). The combined organic layers were washed with brine (2x 100 mL), dried ($Na_2SO_4$) and filtrated over a pad of wool. After removal of the solvent under reduced pressure the crude product was obtained as an orange oil. The oil was dissolved in the least amount of boiling ethanol (~ 10 mL) as possible. After the solution was allowed to cool to room temperature crystallisation was induced by adding a few seed crystals from an earlier batch. The vessel was placed into a freezer for 2-3 days (-20 °C) for quantitative crystallisation. The product was obtained after filtration and washing of the filter cake with ice-cold ethanol as colourless needles. Seed crystals could be obtained by placing the oversaturated ethanol-solution in the fridge (6 °C) for several days. (1.98 mmol, 42%, 0.80 g).
Single crystals suitable for X-ray diffractometry were obtained by slow diffusion of hexane into a saturated solution of the product in $CH_2Cl_2$ at room temperature over the course of several days.
**$^1$H-NMR** (700 MHz, $CDCl_3$) δ: 7.02 (s, Aryl-m-*H*, 2H), 6.96 (s, Aryl-m-*H*, 2H), 3.39 (s, -N*H*$_2$, 2H), 2.35 (s, Aryl-p-C*H*$_3$, 3H), 2.32 (s, Aryl-p-C*H*$_3$, 3H), 2.16 (s, Aryl-o-C*H*$_3$, 6H), 2.06 (s, Aryl-o-C*H*$_3$, 6H).
**$^{13}$C-NMR** (126 MHz, $CDCl_3$) δ: 140.4, 138.9, 138.3, 138.2, 136.4, 130.2 (Aryl-m-*C*), 129.4 (Aryl-m-*C*), ($C_{Trz}$-NH$_2$), 127.7, 126.3, 21.22 (Aryl-p-*C*H$_3$), 21.22 (Aryl-p-*C*H$_3$), 20.12 (Aryl-o-*C*H$_3$), 17.32 (Aryl-o-*C*H$_3$).
**EA:** calc. ($C_{20}H_{24}N_4$, 320.20 g/mol) C 74.97, H 7.55, N 17.48; found C 74.89, H 7.86, N 17.07.
**MS (ESI):** m/z = 321.21 [M+H]$^+$, 343.19.14 [M+Na]$^+$.
**HRMS (ESI):** calc. ($C_{20}H_{24}N_4$) m/z = 321.2076 [M+H]$^+$, found m/z = 321.2074 [M+H]$^+$.

Generation of **1d-O**

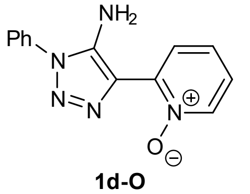

**1d-O**

According to a literature-known procedure[8], the triazole **1d** (307 mg, 1.29 mmol, 1 eq) and m-CPBA (380 mg, 2.59 mmol, 2 eq) were submitted to a roundbottom flask and dissolved in $CHCl_3$ (20 mL). The solution was stirred under reflux for 30 minutes. The solution was allowed to cool to room temperature. The solution was washed with an aqueous $Na_2S_2O_3$-solution. The organic layer was separated and the aqueous layer was extracted with $CH_2Cl_2$ (1x 20 mL). The combined organic layer were washed with aqueous NaOH-solution. The organic layer was separated, dried ($Na_2SO_4$) and filtrated over a pad of wool. The volume was reduced to ~2 mL and the solution was overlayered with EtOH yielding orange single crystals suitable for X-ray diffractometry after several days. Further investigation of the thus obtained crystals by NMR shows that the crsystalline material does not appear to be a well-defined product (Figure S7). A mixture was obtained. Besides diffractometric methods the product composition was confirmed by HRMS
**MS (ESI):** m/z = 254.10 [M+H]$^+$, 276.09 [M+Na]$^+$.
**HRMS (ESI):** calc. ($C_{13}H_{11}N_5O$) m/z = 276.0854 [M+Na]$^+$, found m/z = 276.0854 [M+H]$^+$.

General procedure for triazolium iodides **2a-c** (GP I)

According to a literature-known procedure [9], the appropriate triazole **1a-c** was submitted into an oven-dried Schlenk flask. MeCN and methyliodide were added under a flow of argon. The Schlenk flask was sealed with a stopcock and the mixture was heated to 60 °C. The stopper was lifted to release pressure and the solution was stirred for 3 days at 60 °C in a sealed flask. After 3 days, the reaction mixture was allowed to cool to room temperature and volatiles were removed under reduced pressure. The residue was extracted with $CH_2Cl_2$. Under intense stirring the desired triazolium iodides **2a-c** were precipitated as a fine powder by the addition of $Et_2O$. Filtration and removal of all volatiles under high vacuum yielded the desired product as white to yellowish powders.

Synthesis of **2a** according to GP I

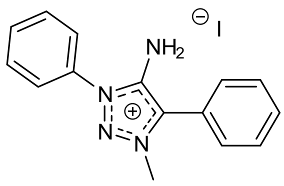


**2a**

- **1a** (406 mg, 1.72 mmol, 1 eq)
- MeCN (15 mL)
- MeI (1.35 g, 12.0 mmol, 7 eq)

Yield: 635 mg (1.68 mmol, 98%) as a yellow powder.
Single crystals suitable for X-ray diffractometry were obtained by slow diffusion of $Et_2O$ into a saturated solution of the product in $CH_2Cl_2$ at -7 °C over night.
**$^1$H-NMR** (250 MHz, $CDCl_3$) δ: 7.89-7.83 (m, Aryl-*H*, 2H), 7.80-7.74 (m, Aryl-*H*, 2H), 7.63-7.54 (m, Aryl-*H*, 6H), 5.07 (s, -N$H_2$, 2H), 4.15 (s. Trz-C$H_3$, 3H).
**$^1$H-NMR** (400 MHz, $CD_3CN$) δ: 7.76-7.70 (m, Aryl-*H*, 5H), 7.68-7.61 (m, Aryl-*H*, 5H), 5.47 (s, -N$H_2$, 2H), 4.05 (s. Trz-C$H_3$, 3H).
**$^{13}$C-NMR** (126 MHz, $CDCl_3$) δ: 143.3, 132.2, 131.9, 130.6, 130.4, 130.1, 125.7, 125.4, 121.9, 39.1 (N-$CH_3$).
**FTIR** (ATR, solid) $\tilde{\nu}$: 1613 ($C_{Trz}$-$N_{exo}$).
**EA:** calc. ($C_{15}H_{15}N_4I$, 378.22 g/mol): C 47.64, H 4.00, N 14.81; found C 47.52, H 4.14, N 14.80.
**MS (ESI):** m/z = 251.13 $[M]^+$.
**HRMS (ESI):** calc. ($C_{15}H_{15}N_4I$) m/z = 251.1291 $[M+H]^+$, found m/z = 251.1283 $[M+H]^+$.

Synthesis of **2b** according to GP I

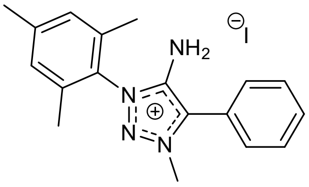


**2b**

- **1b** (264 mg, 0.95 mmol, 1 eq)
- MeCN (10 mL)
- MeI (0.74 g, 6.64 mmol, 7 eq)

Yield: 251 mg (0.69 mmol, 63%) as an off-white powder.
**$^1$H-NMR** (500 MHz, $CDCl_3$) δ: 7.80 (d, *J* = 6.9 Hz, Phenyl-*H*, 2H), 7.63 (t, *J* = 7.6 Hz, Phenyl-*H*, 2H), 7.58 (d, *J* = 7.6 Hz, Phenyl-p-*H*, 1H), 7.09 (s, Aryl-m-*H*, 2H), 5.40 (s, -N$H_2$, 2H), 4.29 (s, $N_{Trz}$-C$H_3$, 3H), 2.40 (s, Aryl-p-C$H_3$, 3H), 2.15 (s, Aryl-o-C$H_3$, 6H).
**$^{13}$C-NMR** (126 MHz, $CDCl_3$) δ: 143.6, 143.2, 135.8, 131.7 (Aryl-m-*C*), 130.4, 130.3, 130.0, 126.8, 124.4 ($C_{Trz}$-$NH_2$), 121.6, 39.8 ($N_{Trz}$-$CH_3$), 21.3 (Aryl-o-$CH_3$), 18.0 (Aryl-p-$CH_3$).
**EA:** calc. ($C_{18}H_{21}N_4I$, 420.30 g/mol): C 51.44, H 5.04, N 13.33; found C 51.31, H 5.08, N 13.03.
**MS (ESI):** m/z = 293.18 $[M]^+$.

**HRMS (ESI):** calc.($C_{18}H_{21}N_4I$) m/z = 293.1761 $[M]^+$, found m/z = 293.1757 $[M]^+$.

Synthesis of **2c** according to GP I

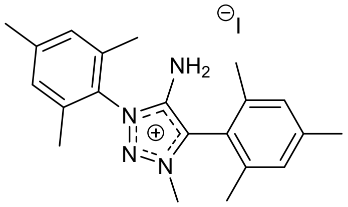

**2c**

- **1c** (421 mg, 1.31 mmol, 1 eq)
- MeCN (10 mL)
- MeI (1.03 g, 9.20 mmol, 7 eq)

Yield: 472 mg (1.02 mmol, 78%) as a yellow powder.

Single crystals suitable for X-ray diffractometry were obtained by slow diffusion of $Et_2O$ into a saturated solution of the product in $CH_2Cl_2$ at -7 °C over night.

**$^1$H-NMR** (500 MHz, $CDCl_3$) δ: 7.07 (s, Aryl-m-*H*, 2H), 7.02 (s, Aryl-m-*H*, 2H), 5.28 (br. s, -N$H_2$, 2H), 3.98 (s, $N_{Trz}$-C$H_3$, 3H), 2.35 (s, Aryl-p-C$H_3$, 3H), 2.32 (s, Aryl-p-C$H_3$, 3H), 2.13 (s, Aryl-o-C$H_3$, 6H), 2.10 (s, Aryl-o-C$H_3$, 6H).

**$^{13}$C-NMR** (126 MHz, $CDCl_3$) δ: 144.1, 143.2, 142.8, 139.0, 135.5, 130.4 (Aryl-m-*C*), 129.7 (Aryl-m-*C*), 126.9, 123.3 ($C_{Trz}$-$NH_2$), 116.7, 38.7 ($N_{Trz}$-$CH_3$), 21.4 (Aryl-p-$CH_3$), 21.3 (Aryl-p-$CH_3$), 20.1 (Aryl-o-$CH_3$), 17.5 (Aryl-o-$CH_3$).

**EA:** calc. ($C_{21}H_{27}N_4I$, 462.38 g/mol): C 54.55, H 5.89, N 12.12; found C 54.32, H 6.00, N 11.47.

**MS (ESI):** m/z = 335.22 $[M]^+$.

**HRMS (ESI):** calc.($C_{21}H_{27}N_4I$) m/z = 335.2230 $[M]^+$, found m/z = 335.2239 $[M]^+$.

Synthesis of **3a**

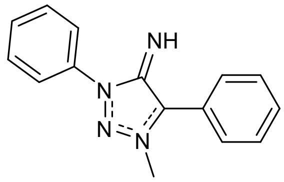

**3a**

Triazolium salt **2a** (300 mg, 0.79 mmol, 1 eq) and potassium hexamethyldisilazide (158 mg, 0.79 mmol, 1 eq) were submitted into an oven-dried Schlenk flask and cooled to -78 °C. THF (30 mL) was added. The reaction mixture was stirred for 30 minutes, warmed to room temperature and then stirred for 1 hour at room temperature. Volatiles were removed under reduced pressure. The residue was suspended in toluene (10 mL) and filtrated over a pad of cellite. The filter cake was extracted with toluene (2x 10 mL). Removal of the solvent under reduced pressure yielded the product as a bright yellow solid (0.64 mmol, 81 %, 160 mg). Pure product was obtained after trituration of the product with small amounts of toluene.

Single crystals suitable for X-ray diffractometry were obtained by storage of a saturated solution of the product in benzene at 4 °C for several weeks.

**$^1$H-NMR** (250 MHz, $C_6D_6$) δ: 8.79-8.68 (m, Aryl-*H*, 2H), 7.15-7.07 (m, Aryl-*H*, 2H), 6.92- 6.88 (m, Aryl-*H*, 4H), 6.86-6.78 (m, Aryl-*H*, 2H), 5.49 (s, br., =N*H*, 1H), 2.60 (s, Trz-C$H_3$, 3H).

**$^1$H-NMR** (500 MHz, $CD_3CN$) δ: 8.25 (dd, *J* = 7.6 Hz, *J* = 1.3 Hz, Aryl-*H*, 2H), 7.58-7.49 (m, Aryl-*H*, 6H), 7.43 (tm, *J* = 6.6 Hz, Aryl-*H*, 1H), 7.33 (tm, *J* = 7.6 Hz, Aryl-*H*, 1H), 4.78 (s, br., =N*H*, 1H), 3.91 (s, $N_{Trz}$-C$H_3$, 3H).

**$^1$H-NMR** (250 MHz, $CD_2Cl_2$) δ: 8.15-8.08 (m, Aryl-*H*, 2H), 7.48-7.29 (m, Aryl-H, 7H), 7.27-7.19 (m, Aryl-*H*, 1H), 4.71 (s, br., =N*H*, 1H), 3.83 (s, Trz-C$H_3$, 3H).

**$^{13}$C-NMR** (126 MHz, $C_6D_6$) δ: 137.5, 127.8, 127.7, 127.0, 126.9, 126.7, 126.5, 124.6, 119.4, 117.1, 35.6 ($N_{Trz}$-$CH_3$).

**$^{13}$C-NMR** (126 MHz, $CD_3CN$) δ: 154.8, 137.3, 128.9, 128.6, 128.1, 127.9, 127.3, 126.0, 120.6, 128.1, 37.8 ($N_{Trz}$-$CH_3$).

**FTIR** (ATR, solid) $\tilde{\nu}$: 1719 ($C_{Trz}$-$N_{exo}$).

**EA:** calc. ($C_{15}H_{14}N_4$, 250.51 g/mol) C 71.98, H 5.64, N 22.38; found. C 71.68, H 5.60, N 22.19.

**MS (ESI):** m/z = 251.13 $[M+H]^+$.

**HRMS (ESI):** calc. ($C_{15}H_{14}N_4$) m/z = 251.1291 $[M+H]^+$, found. m/z = 251.1284 $[M+H]^+$.

Synthesis of **3b**

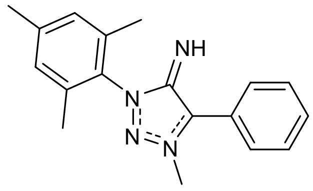

**3b**

Triazolium salt **2b** (154 mg, 0.37 mmol, 1 eq) and potassium hexamethyldisilazide (73 mg, 0.37 mmol, 1 eq) were submitted into an oven-dried Schlenk flask and cooled to -78 °C. THF (10 mL) was added. The reaction mixture was stirred for 30 minutes, warmed to room temperature and then stirred for 1 hour at room temperature. Volatiles were removed under reduced pressure. The residue was suspended in $Et_2O$ (5 mL) and filtrated over cellite. The filtrate was discarded. The filter cake was extracted with toluene (3x 10 mL). Removal of the solvent under reduced pressure yielded the product as an orange solid (0.21 mmol, 56 %, 60 mg).

Single crystals suitable for X-ray diffractometry were obtained by storage of a saturated solution of the product in toluene at -16 °C for several weeks. The obtained structure shows that **3b** and **2b** co-crystallised.

**$^1$H-NMR** (500 MHz, $C_6D_6$) δ: 7.73 (br, Phenyl-*H*, 2H), 7.07 (t, *J* = 7.6 Hz, Phenyl-*H*, 2H), 6.90 (t, *J* = 7.6 Hz, Phenyl-o-*H*, 2H), 6.59 (s, Aryl-m-*H*, 2H), 4.30 (br, Trz=N*H*, 1H), 2.93 (s, Trz-C*H*$_3$, 3H), 2.06 (s, Aryl-o-C*H*$_3$, 6H), 1.92 (s, Aryl-p-C*H*$_3$, 3H).

**$^{13}$C-NMR** (126 MHz, $C_6D_6$) δ: 139.5 (Aryl-p-*C*), 137.3 (Aryl-o-*C*), 129.6, 129.2 (Aryl-m-*C*), 128.7 (Aryl-m-*C*), 127.5, 126.9 ($C_{Trz}$=NH), 117.0 ($C_{Trz}$-Phenyl), 38.4 (N-*C*H$_3$), 21.1 (Aryl-o-*C*H$_3$), 18.0 (Aryl-p-*C*H$_3$).

**EA:** calc. ($C_{18}H_{20}N_4$, 292.39 g/mol) C 73.94, H 6.89, N 19.16; found C 72.96, H 6.55, N 18.75.

CHN-Analysis shows a major deviation from the calculated values. This can be explained by contamination of the starting material **2b** which could not be removed.

**MS (ESI):** m/z = 293.18 $[M+H]^+$.

**HRMS (ESI):** calc. ($C_{18}H_{20}N_4$) m/z = 293.1761 $[M+H]^+$, found m/z = 293.1765 $[M+H]^+$.

Synthesis of **3c**

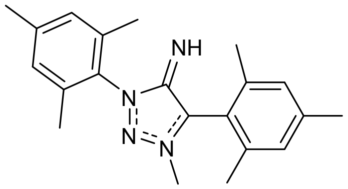

**3c**

Triazolium salt **2c** (230 mg, 0.50 mmol, 1 eq) and potassium hexamethyldisilazide (99 mg, 0.50 mmol, 1 eq) were submitted into an oven-dried Schlenk flask and cooled to -78 °C. THF (15 mL) was added. The reaction mixture was stirred for 30 minutes, warmed to room temperature and then stirred for 1 hour at room temperature. Volatiles were removed under reduced pressure. The residue was suspended in $Et_2O$ (10 mL) and filtrated over cellite. The filter cake was extracted with $Et_2O$ (3x 15 mL). Removal of the solvent under reduced pressure yielded the product as an orange solid (0.30 mmol, 60 %, 100 mg).. Pure **3c** was obtained after trituration with *n*-pentane.

Single crystals suitable for X-ray diffractometry were obtained by slow solvent-evaporation of a solution of the product in $C_6D_6$ at room temperature.

**$^1$H-NMR** (500 MHz, $C_6D_6$) δ: 6.77 (s, Aryl-*H*, 4H), 3.48 (s, br., =N*H*, 1H), 2.86 (s, Trz-C*H*$_3$, 3H), 2.30 (s, Aryl-o-C*H*$_3$, 6H), 2.14 (s, Aryl-o-C*H*$_3$, 6H), 2.13 (s, Aryl-p-C*H*$_3$, 3H), 2.08 (s, Aryl-p-C*H*$_3$, 3H).

**$^{13}$C-NMR** (126 MHz, $C_6D_6$) δ: 139.9, 139.5, 139.2, 137.1, 129.4, 128.9, 128.3, 128.1, 127.9, 36.1 (Trz-$CH_3$), 21.2 (Aryl-p-$CH_3$), 21.1 (Aryl-p-$CH_3$), 19.7 (Aryl-o-$CH_3$), 18.1 (Aryl-o-$CH_3$).

**MS (ESI):** m/z = 335.22 $[M+H]^+$.

**HRMS (ESI):** calc. ($C_{21}H_{26}N_4$) m/z = 335.2230 $[M+H]^+$, found m/z = 335.2230 $[M+H]^+$.

**EA:** calc. ($C_{21}H_{26}N_4$, 334.47 g/mol) C 75.41, H 7.84, N 16.75; found C 75.42, H 7.94, N 16.31.

Synthesis of **2d**

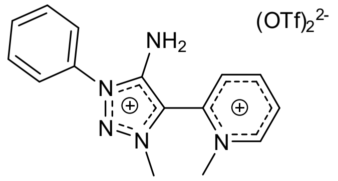


**2d**

Triazole **1d** (460 mg, 1.94 mmol, 1 eq) was submitted into an oven-dried Schlenk flask and dissolved in $CH_2Cl_2$ (25 mL). The solution was cooled to 0 °C and MeOTf (795 mg, 4.85 mmol, 2.5 eq) was added. The solution was stirred for 10 minutes and the stirred over night at room temperature. Volatiles were removed under high-vacuum and the residue was dissolved in acetone (10 mL). The product was precipitated by the addition of $CH_2Cl_2$. The formed solid was filtrated and washed with $CH_2Cl_2$. Removal of volatiles under reduced pressure yielded the product as a white solid (1.14 mmol, 59%, 626 mg).

**$^1$H-NMR** (500 MHz, $CD_3CN$) δ: 9.07 (d, $J$ = 6.3 Hz, Pyridyl-$H$, 1H), 8.75 (t, $J$ = 7.6 Hz, Pyridyl-$H$, 1H), 8.34 (d, $J$ = 8.2 Hz, Pyridyl-$H$, 1H), 8.31 (t, $J$ = 7.3 Hz, Pyridyl-$H$, 1H), 7.81-7.73 (m, Phenyl-$H$, 5H), 6.30 (s, br., Trz-N$H_2$, 2H), 4.35 (s, $N_{Py}$-C$H_3$, 3H), 4.13 (s, $N_{Trz}$-C$H_3$, 3H).

**$^{13}$C-NMR** (126 MHz, $CD_3CN$) δ: 149.2, 146.8, 145.8, 133.3, 132.2, 131.4, 130.5, 130.4, 125.5, 120.4 (q, $^1J_{CF}$ = 320 Hz, $SO_3CF_3$), 46.6 ($N_{Py}$-$CH_3$), 39.4 ($N_{Trz}$-$CH_3$).

**$^{19}$F-NMR** (235 MHz, $CD_3CN$) δ: - 78.3 (s, $SO_3CF_3$)

**EA:** calc. ($C_{17}H_{17}F_6N_5O_6S_2$, 565.46 g/mol) C 36.11, H 3.03, N 12.39; found C 36.11, H 3.21, N 12.45.

**MS (ESI):** m/z = 148.95 $[OTf]^-$, 236.12 $[M]^{2+}$.

**HRMS (ESI):** calc. ($C_{16}H_{14}F_3N_3O_3S_2$), m/z = 236.1182 $[M]^{2+}$, found. m/z = 236.1180 $[M]^{2+}$.

Preparation of **1d-O** single crystals suitable for X-ray diffractometry

According to a reported protocol [8], a round-bottom flask was equipped with a stir bar, the triazole **1d** (307 mg, 1.29 mmol, 1 eq) and mCPBA (580 mg, 2.59 mmol, 2 eq). Distilled chloroform (20 mL) was added and the solution was stirred under reflux for 30 Min. The solution was allowed to cool to room temperature. The organic phase was washed with aqueous $Na_2S_2O_3$-solution, followed by washing with aqueous NaOH-solution. The combined aqueous layers were extracted with $CH_2Cl_2$ (2x 10 mL). The combined organic layers were dried ($Na_2SO_4$) and filtrated. A crude product mixture was obtained after removal of the solvent under reduced pressure as a highly viscous red oil. Single crystals were obtained after several days by over layering a concentration solution of the crude product in $CH_2Cl_2$ with EtOH at room temperature.

Synthesis of **5a**

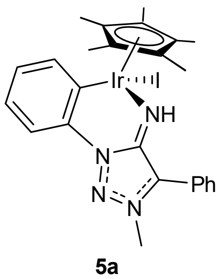


**5a**

The ligand precursor **2a** (18 mg, 0.05 mmol, 1 eq), NaOAc (23 mg, 0.29 mmol, 6 eq) and [Ir(Cp*)Cl$_2$]$_2$ (19 mg, 0.02 mmol, 0.5 eq) were submitted into an oven-dried Schlenk flask. Under a flow of argon, MeCN (6 mL) was added and the flask was sealed. The mixture was heated to 65 °C, the stop-cock was lifted to release pressure and the mixture was stirred at 65 °C in the sealed flask for three days. The mixture was allowed to cool to room temperature. The suspension was filtered through a pad of cellite and the solvent in the filtrate was removed under reduced pressure. The residue was suspended in Et$_2$O (10 mL) and sonicated with ultra-sound for 1 hour. The resulting red solid was filtrated and washed with small amounts of ice-cold Et$_2$O. The product was obtained in a pure form as red needles after crystallisation through slow diffusion of *n*-pentane at room temperature into a concentrated solution of the crude product in CH$_2$Cl$_2$ over the course of several days (0.04 mmol, 75%, 25 mg).

Single crystals suitable for X-ray diffractometry were obtained the same way.

**$^1$H-NMR** (700 MHz, CD$_2$Cl$_2$) δ: 7.80 (dd, *J* = 7.6 Hz, *J* = 1.4 Hz, Phenyl-*H*, 1H), 7.56 (t, *J* = 7.6 Hz, Phenyl-*H,* 2H), 7.55-7.51 (m, Phenyl-*H*, 2H), 7.47-7.45 (m, Phenyl-*H,* 2H), 6.96 (dt, *J* = 7.3 Hz, *J* = 1.3 Hz, Phenyl-*H*, 1H), 6.86 (dt, *J* = 7.5 Hz, *J* = 1.4 Hz, Phenyl-*H*, 1H), 3.92 (s, Trz-C*H*$_3$, 3H), 3.50 (s, =N*H*, 1H), 1.48 (s, Cp-(C*H*$_3$)$_5$, 15 H).

**$^{13}$C-NMR** (176 MHz, CD$_2$Cl$_2$) δ: 147.3, 147.1, 136.5, 135.4, 129.8, 129.2, 126.4, 125.2, 122.8, 120.9, 114.1, 87.4 ($C_{Cp*}$), 38.0 (Trz-C*H*$_3$), 9.1 (Cp-(*C*H$_3$)$_5$).

**EA:** calc. (C$_{25}$H$_{28}$IIrN$_4$, 703.65 g/mol) C 42.67, H 4.01, N 7.96; found C 41.49, H 3.92, N 7.70.

The low carbon content in CHN-analysis is presumably affiliated with the formation of iridium carbides during the combustion method as the hydrogen/nitrogen content fit the calculated values and no impurities were detected spectroscopically.

**MS (ESI):** m/z = 577.19 [M-I]$^+$, 251.13 [**2a**]$^+$.

**HRMS (ESI):** calc. (C$_{28}$H$_{34}$IIrN$_4$), m/z = 577.1938 [M-I]$^+$, found. m/z = 577.1934 [M-I]$^+$.

Synthesis of **5b**

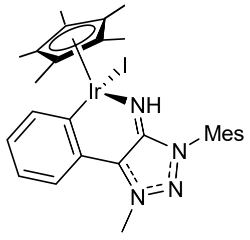


**5b**

The ligand precursor **2b** (28 mg, 0.07 mmol, 1 eq), NaOAc (33 mg, 0.40 mmol, 6 eq) and [Ir(Cp*)Cl$_2$]$_2$ (27 mg, 0.03 mmol, 0.5 eq) were submitted into an oven-dried Schlenk flask. Under a flow of argon, MeCN (6 mL) was added and the flask was sealed. The mixture was heated to 65 °C, the stop-cock was lifted to release pressure and the mixture was stirred at 65 °C in the sealed flask for three days. The mixture was allowed to cool to room temperature. The suspension was filtered through cellite and the solvent in the filtrate was removed under reduced pressure. The residue was suspended in Et$_2$O (10 mL) and sonicated with ultra-sound for 1 hour. The resulting red solid was filtrated and washed with small amounts of ice-cold Et$_2$O. The product was obtained in a pure form as red plates after crystallisation through slow diffusion of *n*-pentane at room temperature into a concentrated solution of the crude product in CH$_2$Cl$_2$ over the course of several days (0.03 mmol, 40%, 20 mg).

Single crystals suitable for X-ray diffractometry were obtained the same way.

**$^1$H-NMR** (500 MHz, CD$_2$Cl$_2$) δ: 7.87 (d, *J* = 7.6 Hz, Phenyl-H, 1H), 7.47 (d, *J* = 7.6 Hz, Phenyl-H, 1H), 7.00 (s, Aryl-m-*H*, 2H), 6.85-6.77 (m, Phenyl-H, 2H), 4.17 (s, Trz-C*H*$_3$, 3H), 2.77 (s, br., =N*H*, 1H), 2.29 (s, Aryl-p-C*H*$_3$, 3H), 2.04 (s, Aryl-o-C*H*$_3$, 6H), 1.45 (s, Cp-(C*H*$_3$)$_5$, 15 H).

**$^{13}$C-NMR** (126 MHz, CD$_2$Cl$_2$) δ: 148.8, 146.8, 141.7, 136.9, 130.2, 129.3, 128.5, 126.0, 125.1, 122.4, 119.4, 87.8 ($C_{Cp*}$), 41.9 (Trz-C*H*$_3$), 21.3 (Aryl-p-*C*H$_3$), 18.0 (Aryl-o-*C*H$_3$), 9.4 (Cp-(*C*H$_3$)$_5$).

**EA:** calc. (C$_{28}$H$_{34}$IIrN$_4$, 743.71 g/mol) C 45.10, H 4.60, N 7.51; found C 43.79, H 4.53, N 7.13.

The low carbon content in CHN-analysis is presumably affiliated with the formation of iridium carbides during the combustion method as the hydrogen/nitrogen content fit the calculated values and no impurities were detected spectroscopically.

**MS (ESI):** m/z = 619.24 [M-I]$^+$, 293.18 [**2b**]$^+$.

**HRMS (ESI):** calc. (C$_{28}$H$_{34}$IIrN$_4$), m/z = 619.2408 [M-I]$^+$, found. m/z = 619.2412 [M-I]$^+$.

Synthesis of **5d**

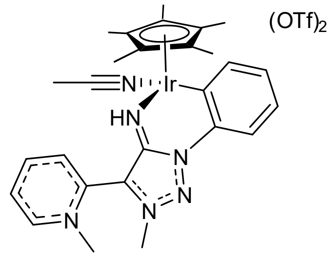

**5d**

The ligand precursor **2d** (147 mg, 0.26 mmol, 1 eq), NaOAc (128 mg, 1.56 mmol, 6 eq) and $[Ir(Cp^*)Cl_2]_2$ (104 mg, 0.13 mmol, 0.5 eq) were submitted into an oven-dried Schlenk flask. Under a flow of argon, MeCN (15 mL) was added and the flask was sealed. The mixture was heated to 65 °C, the stop-cock was lifted to release pressure and the mixture was stirred at 65 °C in the sealed flask over night. The mixture was allowed to cool to room temperature. The suspension was filtered through cellite. The solvent in the filtrate was reduced to ~2 mL and the product obtained in a pure form after precipitation by addition of $Et_2O$ as an orange powder (0.17 mmol, 66%, 160 mg).

Single crystals suitable for X-ray diffractometry were obtained as red needles by slow diffusion of $Et_2O$ at room temperature into a concentrated solution of the product in MeCN over night.

**$^{1}$H-NMR** (700 MHz, $CD_3CN$) δ: 9.00 (d, *J* = 6.1 Hz, Aryl-*H*, 1H), 8.68 (dt, *J* = 7.9 Hz, *J* = 1.9 Hz, Aryl-*H*, 1H), 8.28 (dd, *J* = 7.8 Hz, *J* = 1.2 Hz, Aryl-*H*, 1H), 8.21-8.18 (m, Aryl-*H*, 1H), 7.81-7.78 (m, Aryl-*H*, 1H), 7.69-7.67 (m, Aryl-*H*, 1H), 7.17-7.15 (m, Aryl-*H*, 2H), 4.86 (s, =N*H*, 1H), 4.22 (s, $N_{Py}$-C*H*$_3$, 3H), 3.98 (s, $N_{Trz}$-C*H*$_3$, 3H), 1.96 (s, MeCN), 1.53 (s, Cp-(C*H*$_3$)$_5$, 15 H).

**$^{13}$C-NMR** (176 MHz, $CD_3CN$) δ: 149.7, 148.1, 147.2, 143.3, 140.4, 135.9, 135.0, 133.2, 129.8, 128.6, 125.5, 121.6 (q, $^{1}J_{CF}$ = 321 Hz, $SO_3$*C*$F_3$), 116.4, 112.1, 90.3 (*C*$_{Cp^*}$), 47.3 ($N_{Py}$-*C*$H_3$), 39.8 (Trz-*C*$H_3$), 8.5 (Cp-(*C*$H_3$)$_5$).

**EA:** calc. ($C_{29}H_{33}F_6IrN_6O_6S_2$, 931.95 g/mol) C 37.38, H 3.57, N 9.02; found C 37.35, H 3.61, N 8.80.

**MS (ESI):** m/z = 742.16 [M-(OTf)-(MeCN)]$^+$, 594.22 [M-OTf-MeCN+H]$^+$, 296.61 [M-$(OTf)_2$-MeCN]$^{2+}$.

**HRMS (ESI):** calc. ($C_{29}H_{33}F_6IrN_6O_6S_2$), m/z = 742.1645 [M-(OTf)-(MeCN)]$^+$, found. m/z = 742.1646 [M-(OTf)-(MeCN)]$^+$.

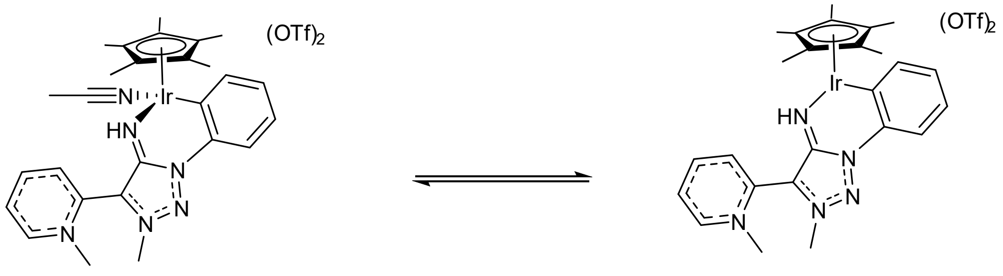

**5d** **5d-acetone**

The auxiliary MeCN-ligand could be removed by solving **5** in acetone followed by precipitation with $Et_2O$ as a green powder. Crystallisation by diffusion of either $Et_2O$ or *n*-pentane at room temperature into a concentrated solution of **5d-acetone** in acetone over the course of several days yielded a pure product in the form of green plates. Single crystals suitable for X-ray diffractometry were obtained as green plates following the same procedure.

**$^{1}$H-NMR** (500 MHz, $(D_3C)_2CO$) δ: 11.30 (s, br., =N*H*, 1H), 9.44 (d, *J* = 6.3 Hz, Aryl-*H*, 1H), 9.16 (d, *J* = 7.8 Hz, Aryl-*H*, 1H), 8.93 (t, *J* = 7.8 Hz, Aryl-*H*, 1H), 8.72 (d, *J* = 7.7 Hz, Aryl-*H*, 1H), 8.58 (d, *J* = 8.4 Hz, Aryl-*H*, 1H), 8.47 (t, *J* = 6.8 Hz, Aryl-*H*, 1H), 7.75 (t, *J* = 7.7 Hz, Aryl-*H*, 1H), 7.52 (t, *J* = 7.3 Hz, Aryl-*H*, 1H), 4.63 (s, $N_{Py}$-C*H*$_3$, 3H), 4.45 (s, $N_{Trz}$-C*H*$_3$, 3H), 1.90 (s, Cp-(C*H*$_3$)$_5$, 15 H).

**$^{13}$C-NMR** (126 MHz, $(D_3C)_2CO$) δ: 151.8, 150.9, 147.7, 145.4, 140.1, 135.3, 134.4, 131.2, 129.3, 127.5, 122.1 (q, $^{1}J_{CF}$ = 321 Hz, $SO_3$*C*$F_3$), 117.9, 113.7, 94.5 (*C*$_{Cp^*}$), 47.8 ($N_{Py}$-*C*$H_3$), 40.6 (Trz-*C*$H_3$), 10.2 (Cp-(*C*$H_3$)$_5$).

**EA:** calc. ($C_{27}H_{30}F_6N_5O_6S_2Ir$, 890.89 g/mol) C 36.40, H 3.39, N 7.86; found C 36.55, H 3.45, N 7.71.

Synthesis of **3c-Rh(CO)$_2$Cl**

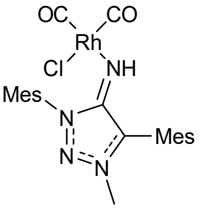

**3c-Rh(CO)$_2$Cl**

The ligand **3c** (62 mg, 0.19 mmol, 1 eq) and [Rh(CO)$_2$Cl]$_2$ (36 mg, 0.09 mmol, 0.5 eq) were submitted into an oven-dried Schlenk flask. Under a flow of argon, THF (5 mL) was added and the solution was stirred at room temperature over night. The solvent was removed under reduced pressure and the residue was suspended in Et$_2$O (5 mL). The solution was filtrated over cellite. The filter cake was extracted with Et$_2$O (2x 5 mL). The solvent in the residue was removed under reduced pressure yielding **3c-Rh(CO)$_2$Cl** a pale yellow powder in a pure form (0.04 mmol, 41%, 40 mg).

Single crystals suitable for X-ray diffractometry were obtained as yellow plates by slow diffusion of *n*-pentane at room temperature into a concentrated solution of the product in toluene over the course of several weeks.

**$^1$H-NMR** (250 MHz, C$_6$D$_6$) δ: 6.83 (s, Aryl-*H*, 2H), 6.68 (s, Aryl-*H*, 2H), 2.96 (s, br., =N*H*, 1H), 2.68 (s, Trz-C*H*$_3$, 3H), 2.18 (s, Aryl-o-C*H*$_3$, 12H), 2.14 (s, Aryl-p-C*H*$_3$, 3H), 2.03 (s, Aryl-p-C*H*$_3$, 3H).

**$^{13}$C-NMR** (63 MHz, C$_6$D$_6$) δ: 184.7 (d, $^1J_{CRh}$ = 65 Hz, Rh-*C*O), 180.8 (d, $^1J_{CRh}$ = 74 Hz, Rh-*C*O), 151.7, 140.1, 140.0, 139.0, 135.7, 128.8, 128.1, 128.0, 120.4, 119.2, 35.0, 20.1, 19.9, 19.1, 16.7.

**EA:** calc. (C$_{23}$H$_{26}$ClN$_4$O$_2$Rh, 528.84 g/mol) C 52.24, H 4.96, N 10.59; found C 52.23, H 4.98, N 10.51.

**MS (EI):** m/z = 528.1 $[M]^{+\cdot}$.

3. NMR-spectra

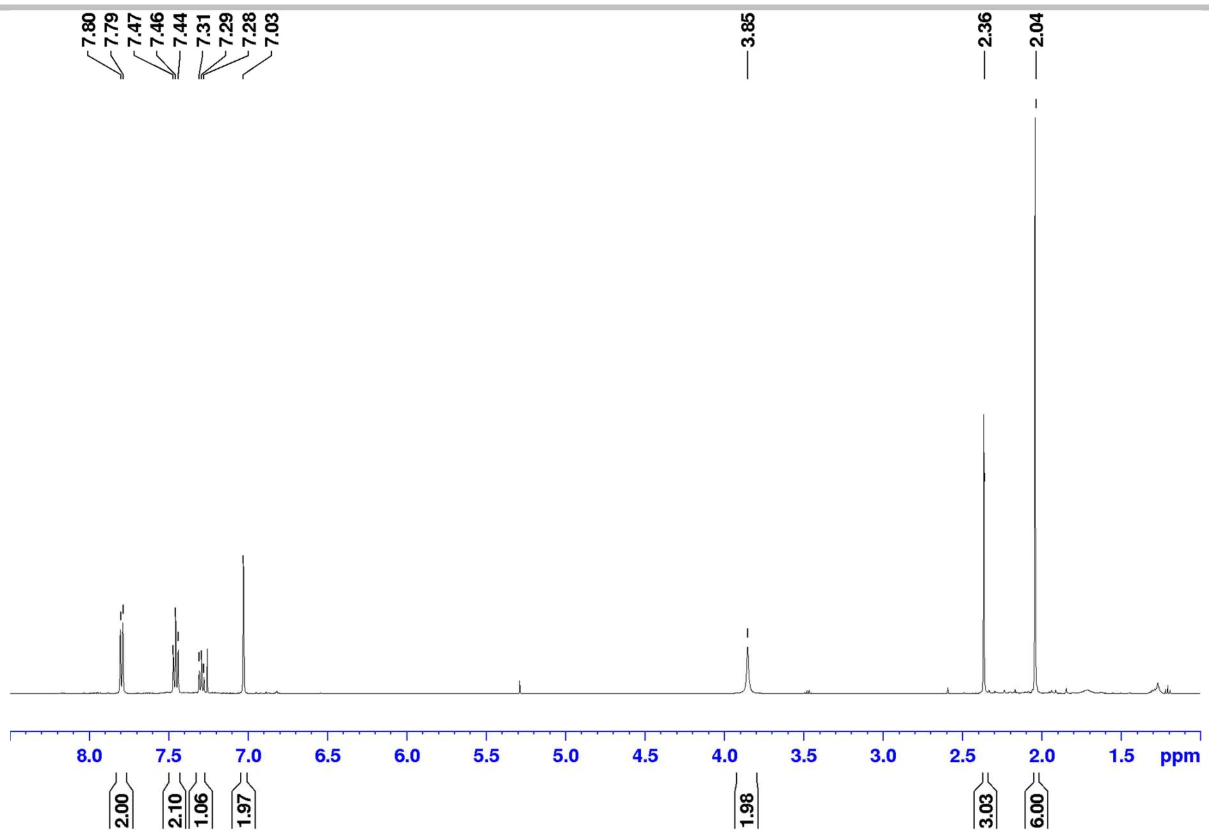


**Figure S1.** $^{1}H$-NMR-spectrum of **1b** ($CDCl_3$, 500 MHz).

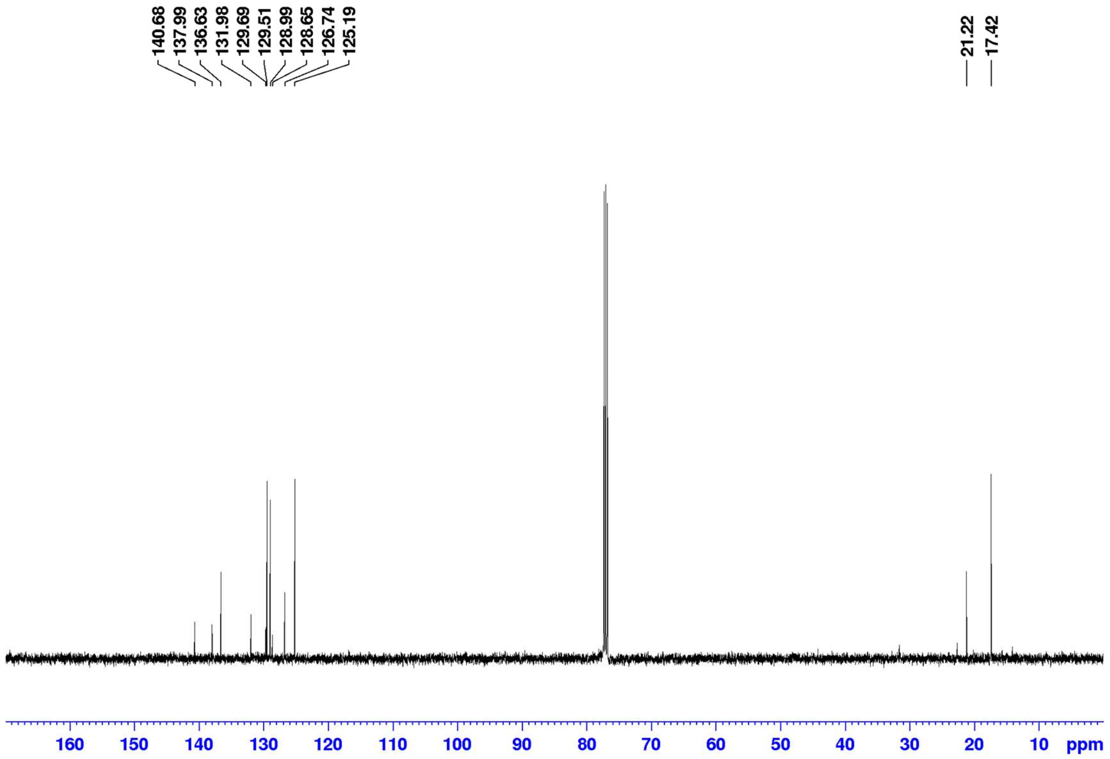


**Figure S2.** $^{13}C\{^{1}H\}$-NMR-spectrum of **1b** ($CDCl_3$, 126 MHz).

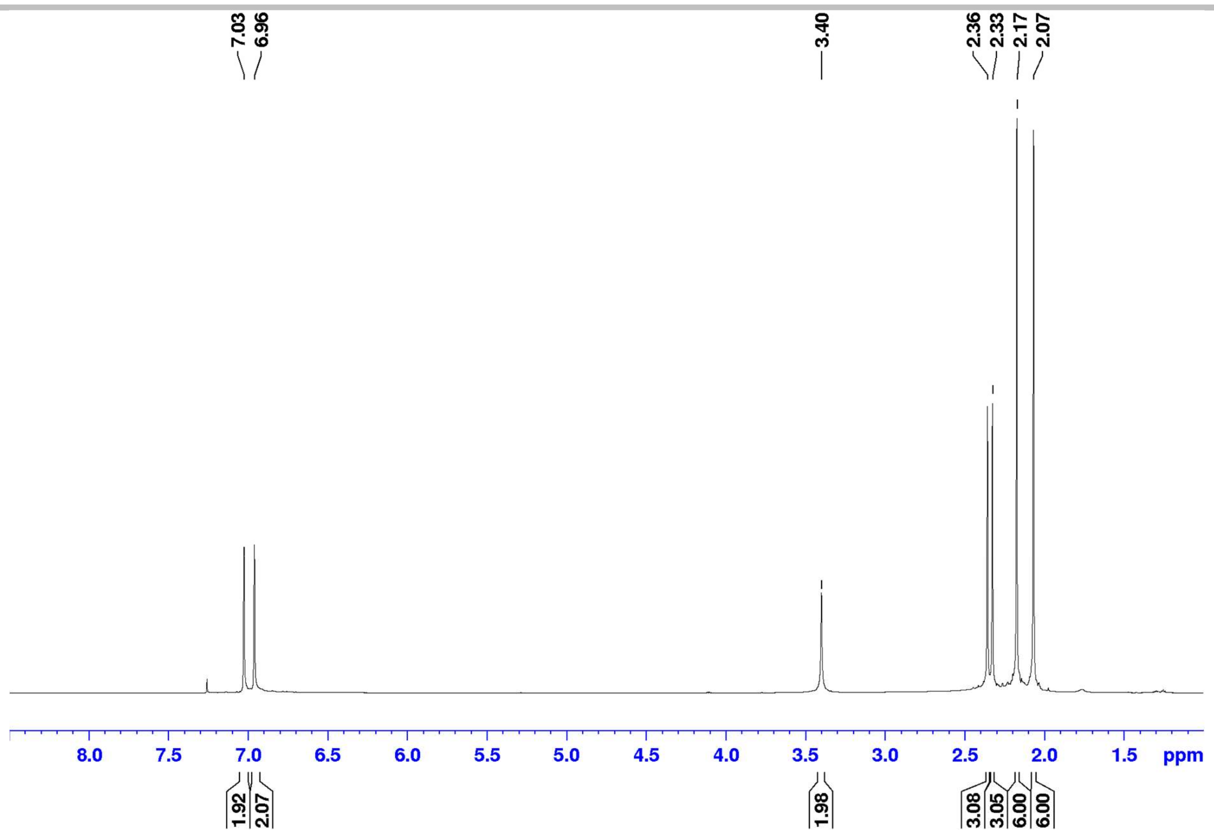


**Figure S3.** $^{1}$H-NMR-spectrum of **1c** ($CDCl_3$, 700 MHz).

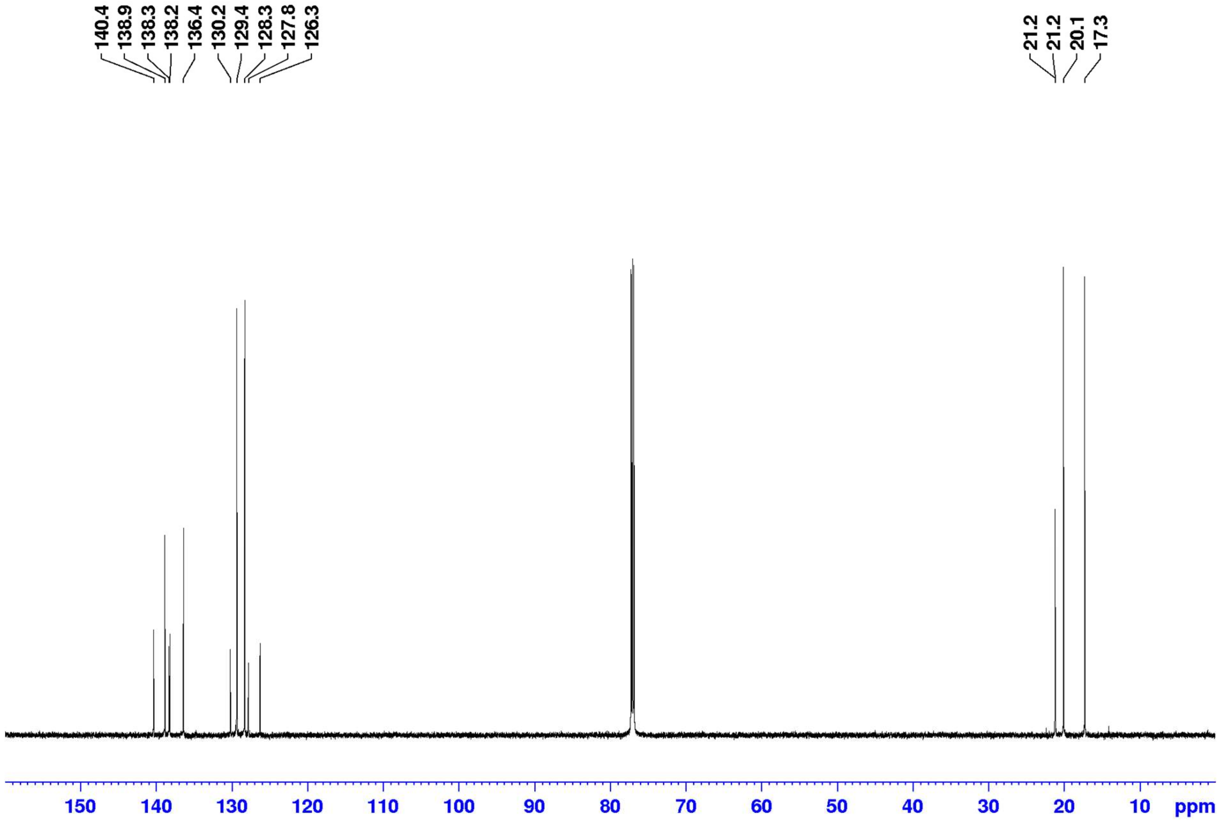


**Figure S4.** $^{13}$C{$^{1}$H]-NMR-spectrum of **1c** ($CDCl_3$, 176 MHz).

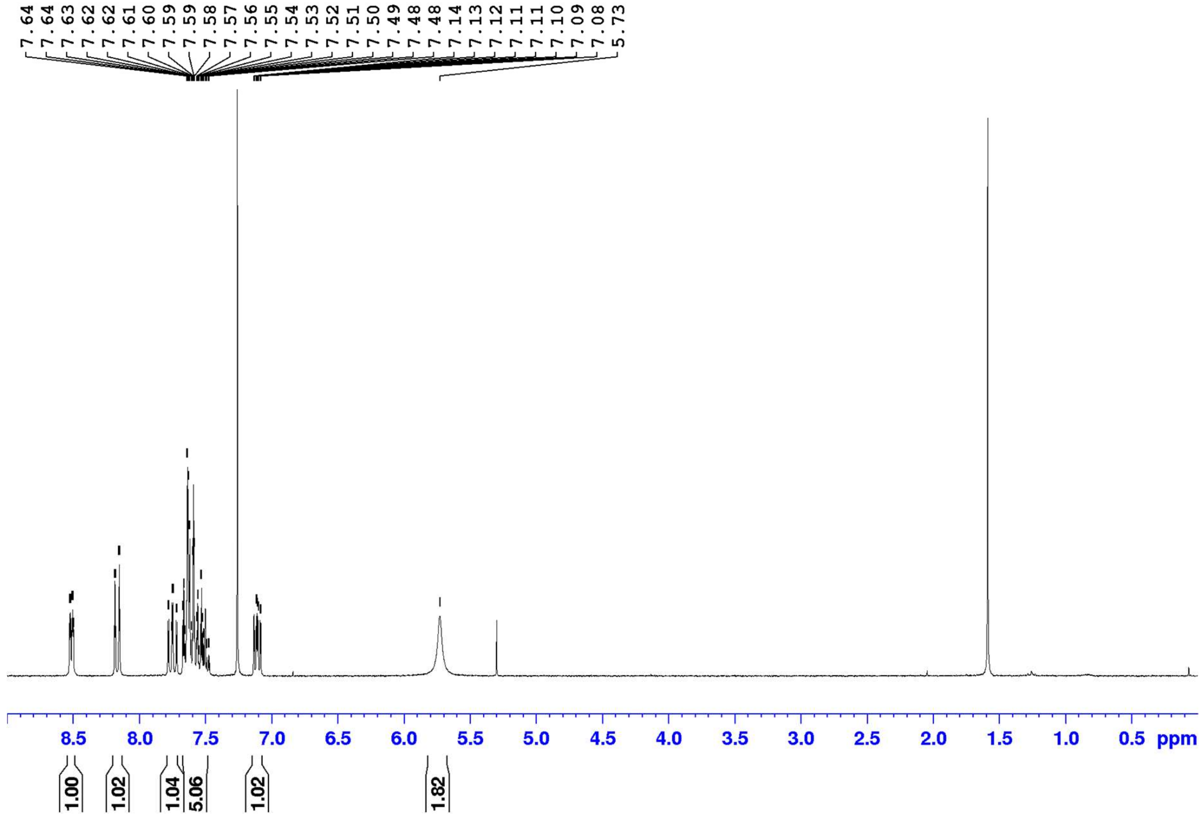


**Figure S5.** ¹H-NMR-spectrum of **1d** ($CDCl_3$, 250 MHz).

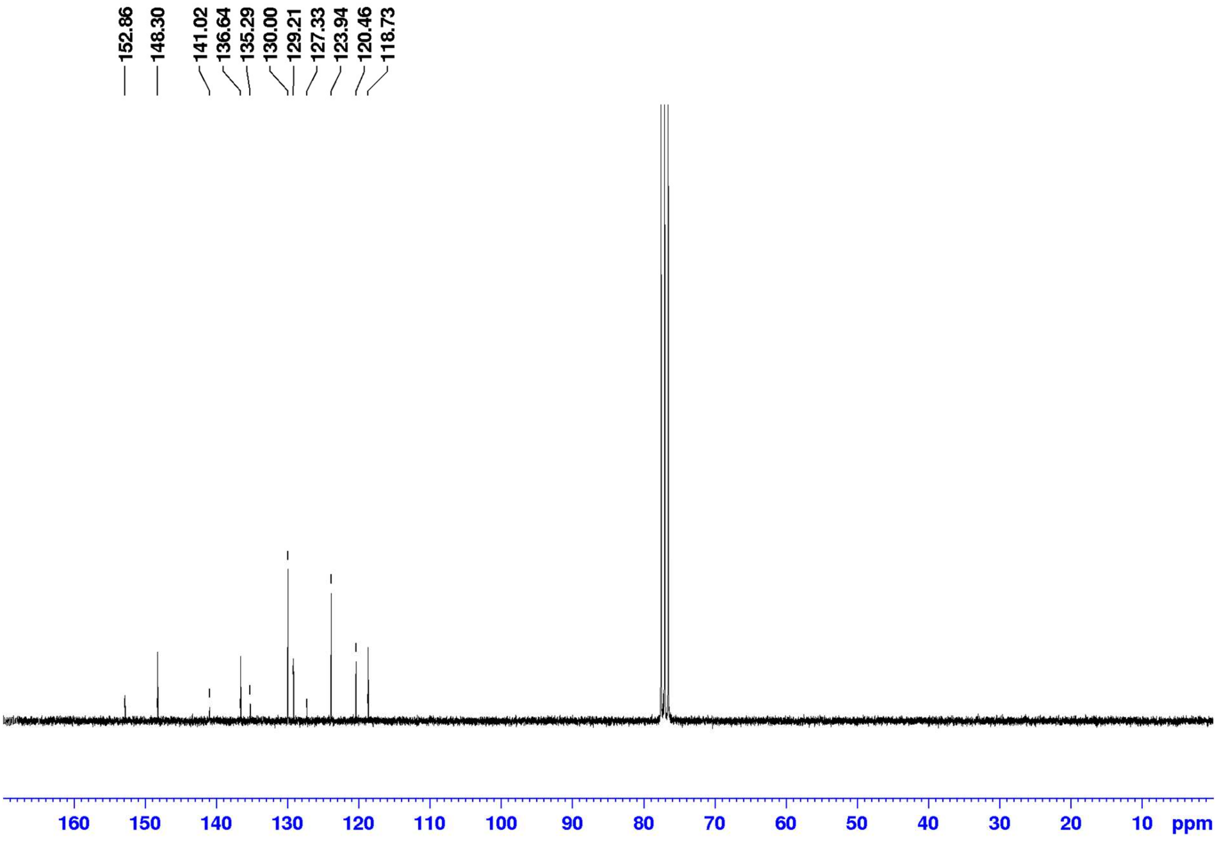


**Figure S6.** $^{13}C\{^1H\}$-NMR-spectrum of **1d** ($CDCl_3$, 63 MHz).

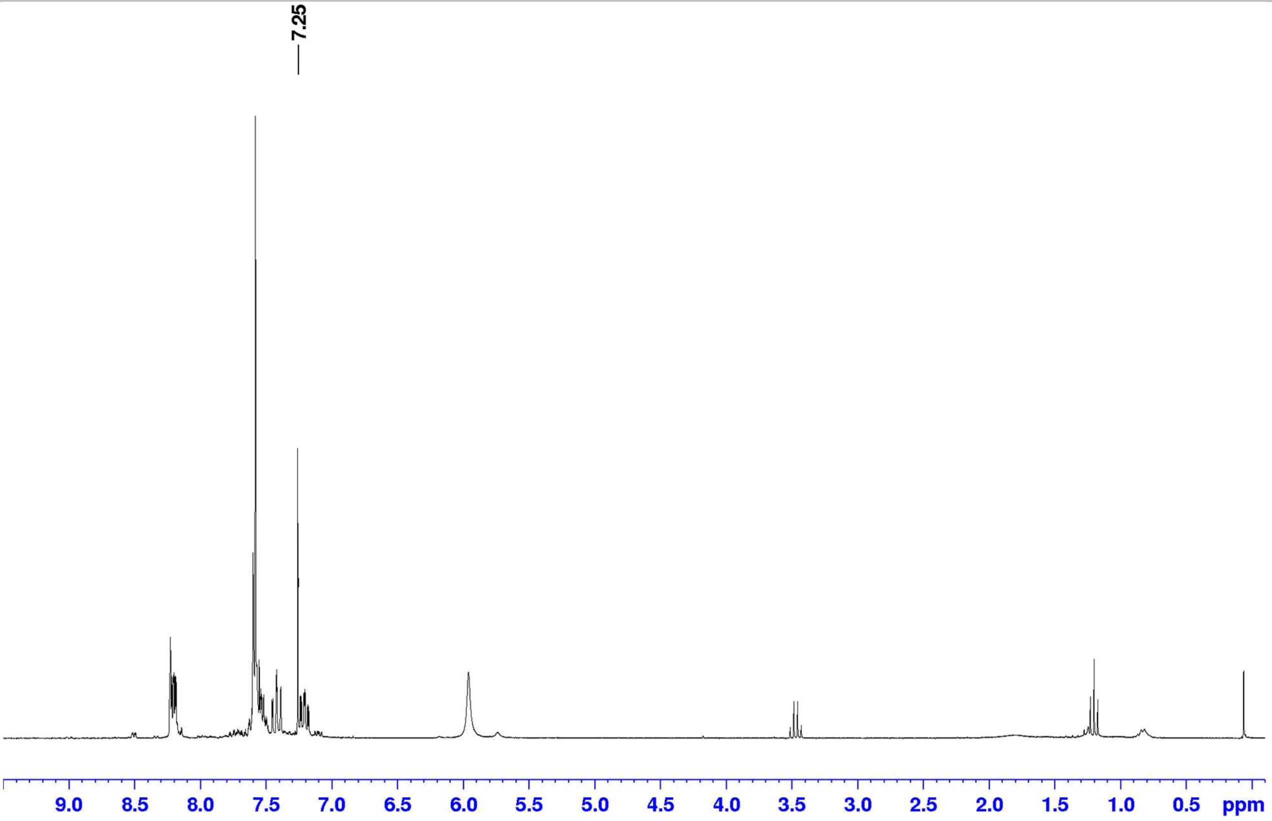


**Figure S7.** $^{1}$H-NMR-spectrum of the reaction of **1d** with m-CPBA ($CDCl_3$, 250 MHz).

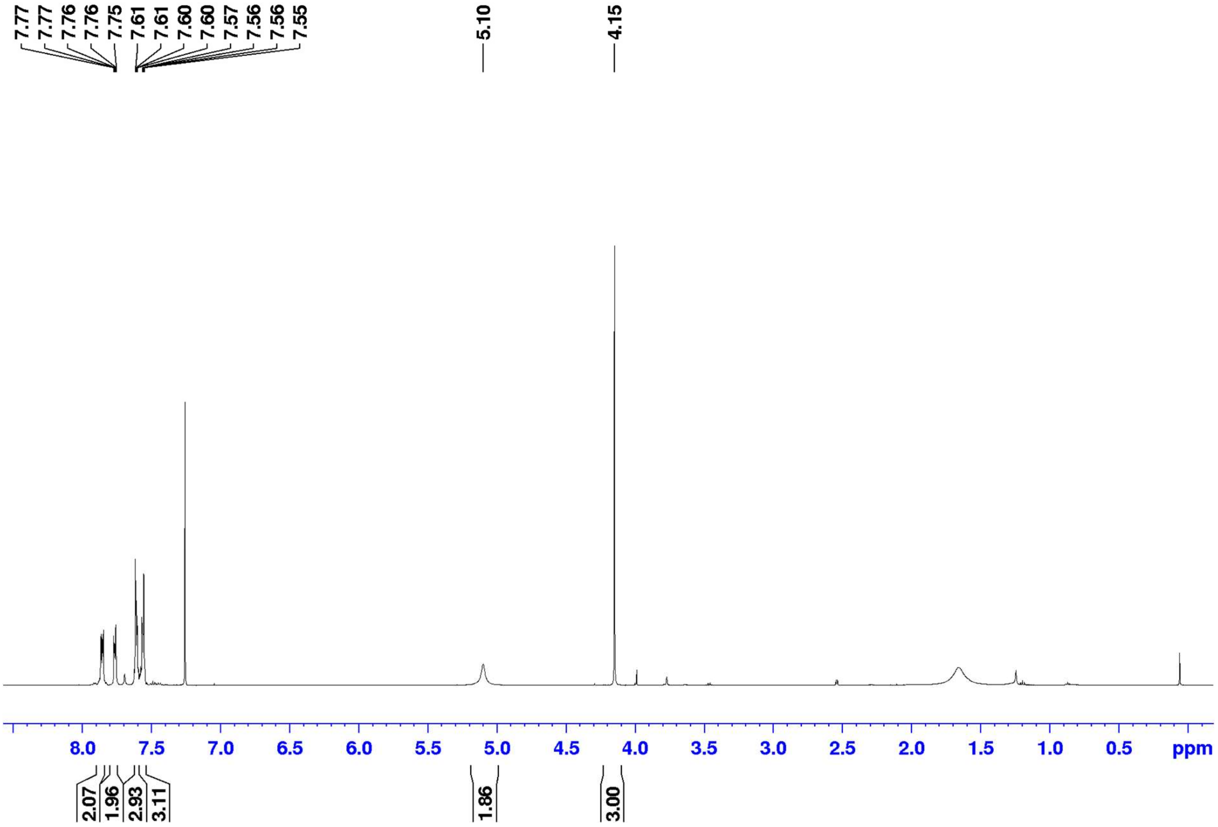


**Figure S8.** $^{1}$H-NMR-spectrum of **2a** ($CDCl_3$, 500 MHz).

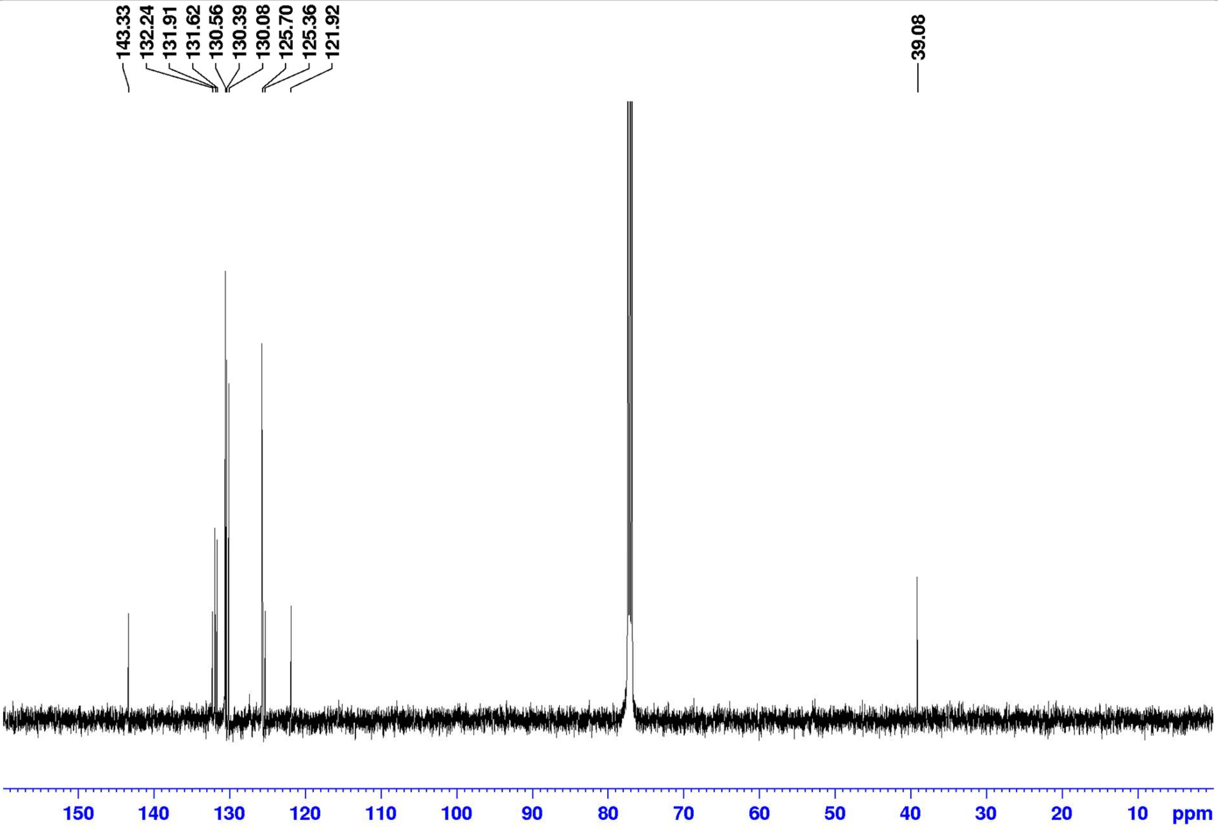


**Figure S9.** $^{13}C\{^1H\}$-NMR-spectrum of **2a** ($CDCl_3$, 126 MHz).

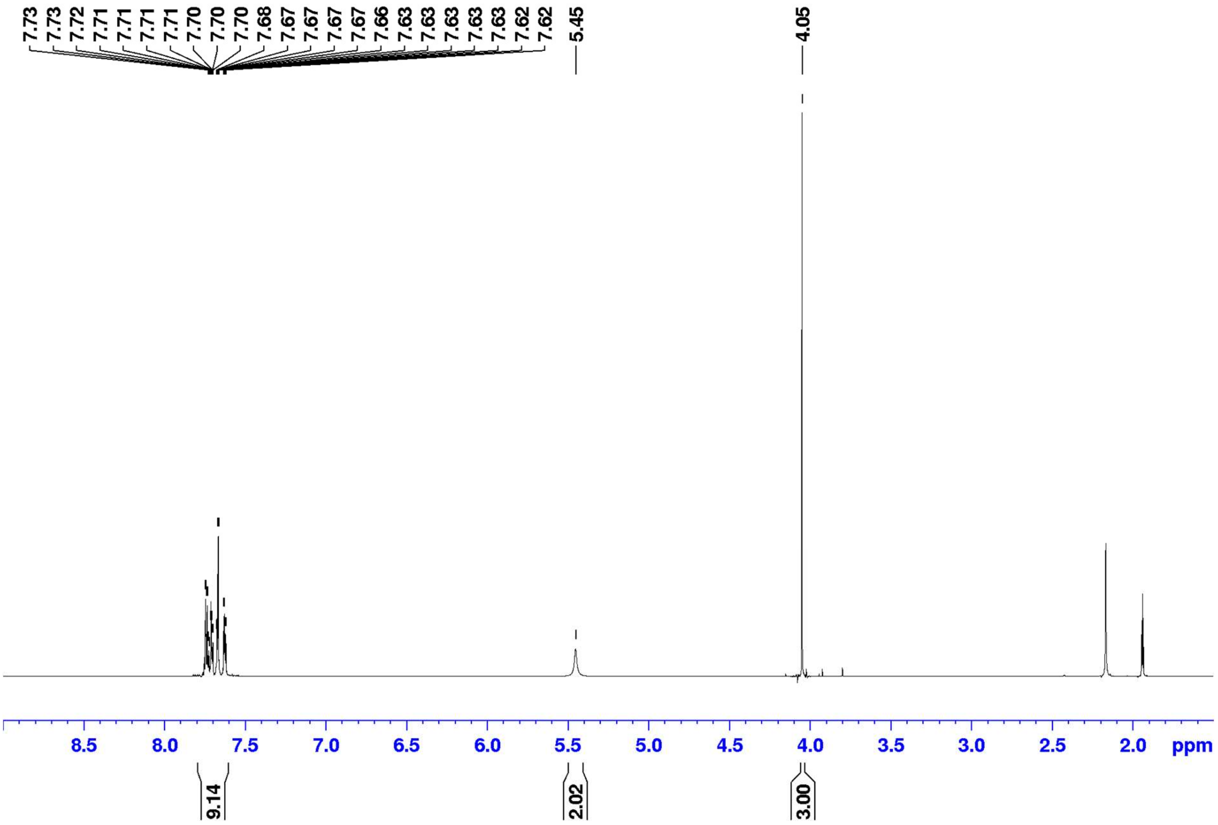


**Figure S10.** $^1H$-NMR-spectrum of **1d** ($CD_3CN$, 700 MHz).

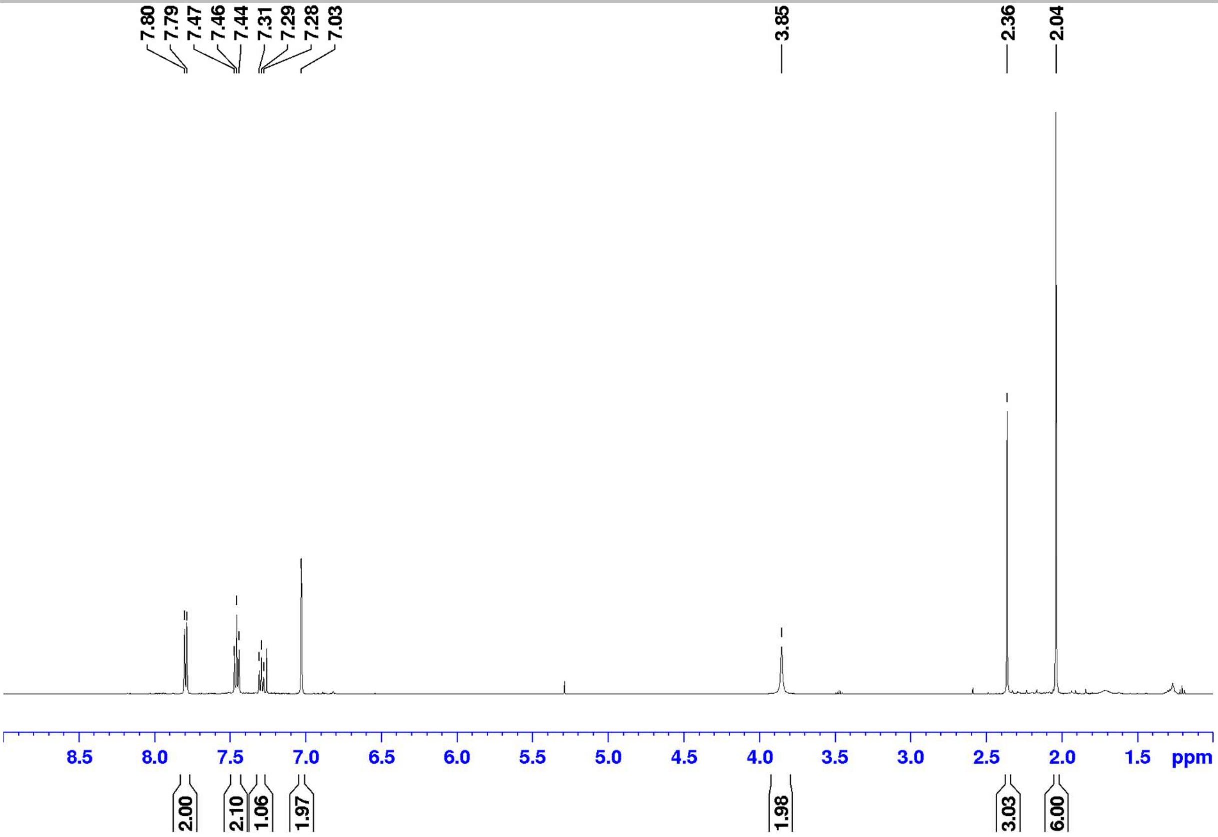


**Figure S11.** $^{1}$H-NMR-spectrum of **2b** ($CDCl_3$, 500 MHz).

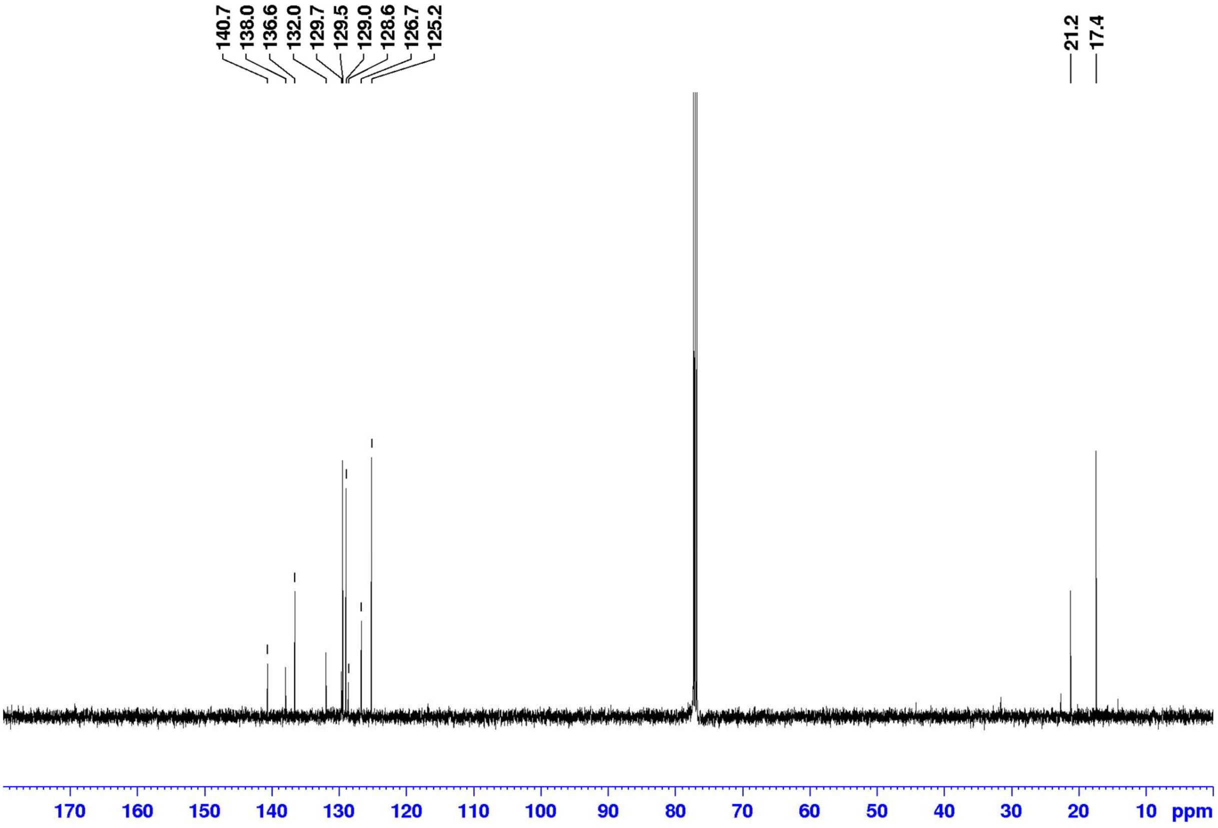


**Figure S12.** $^{13}$C{$^{1}$H}-NMR-spectrum of **2b** ($CDCl_3$, 126 MHz).

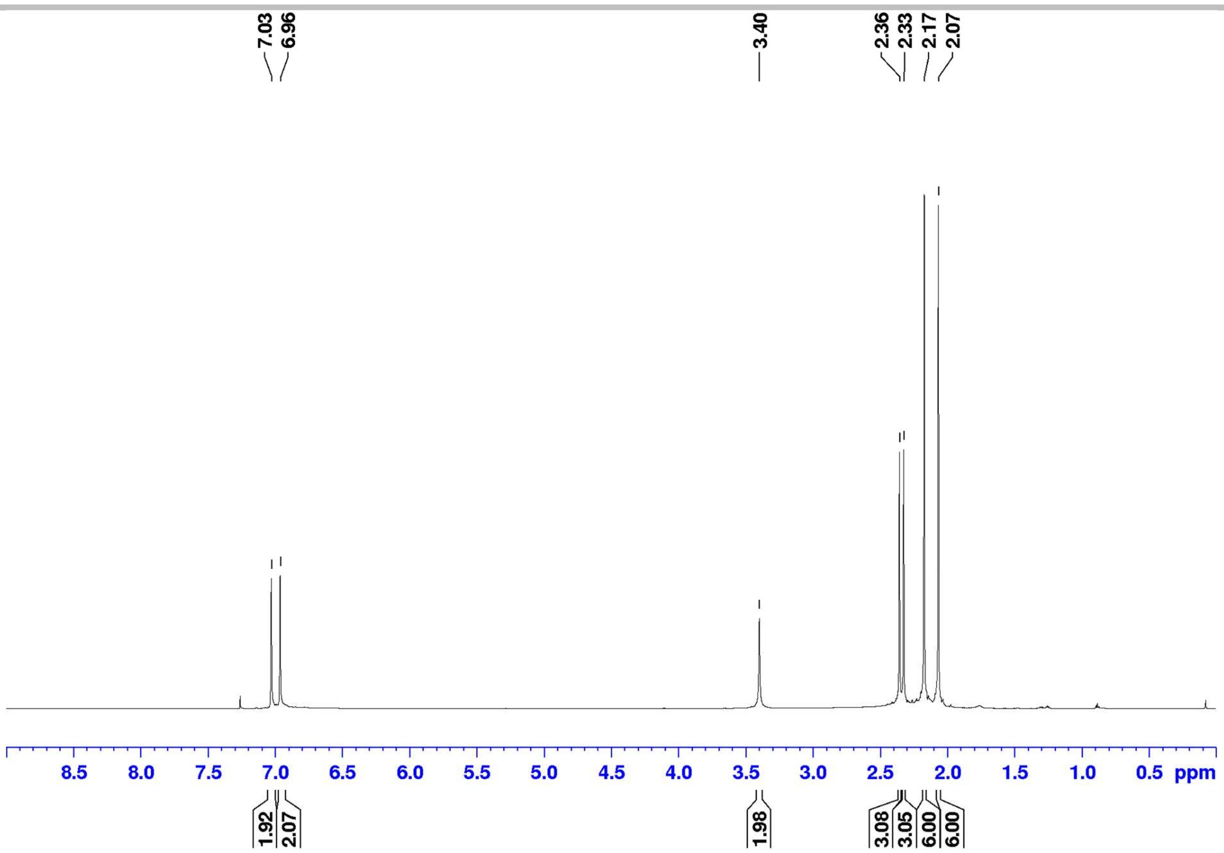


**Figure S13.** $^{1}H$-NMR-spectrum of **2c** ($CDCl_3$, 700 MHz).

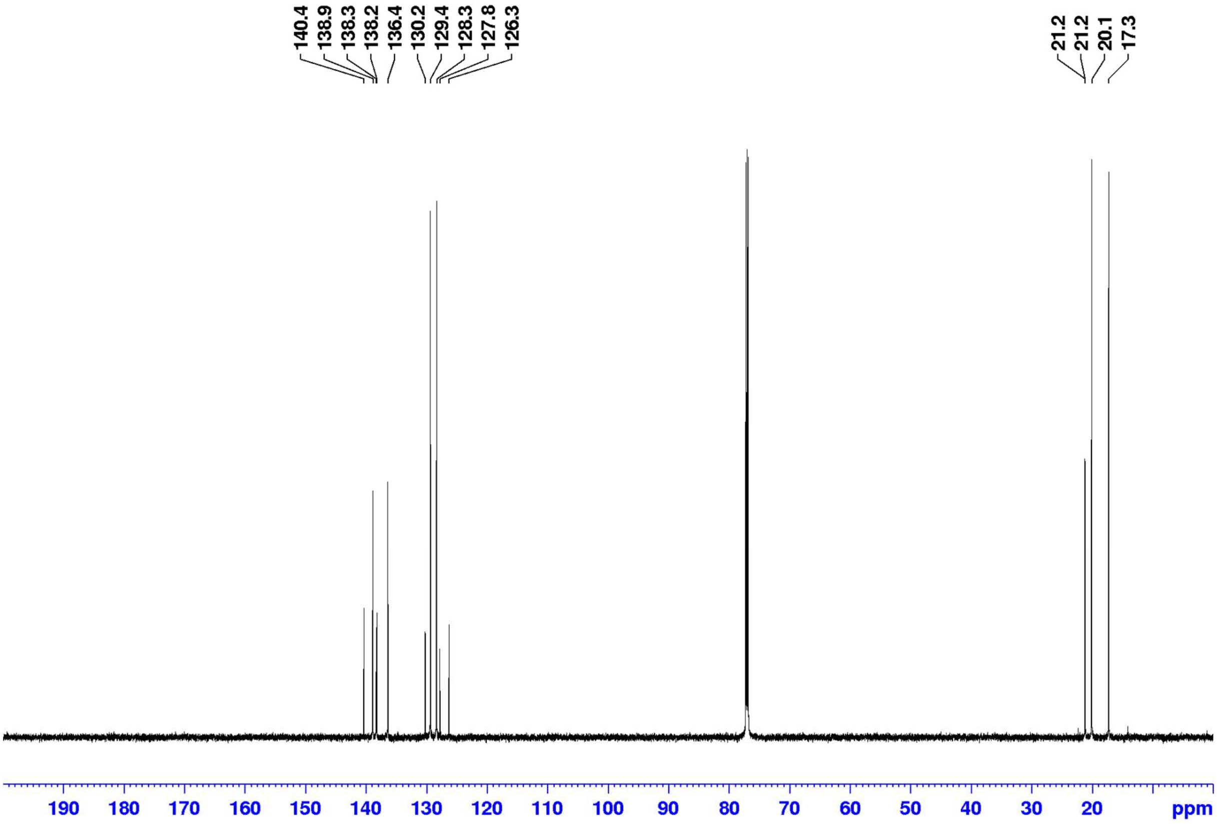


**Figure S14.** $^{13}C\{^{1}H\}$-NMR-spectrum of **2c** ($CDCl_3$, 176 MHz).

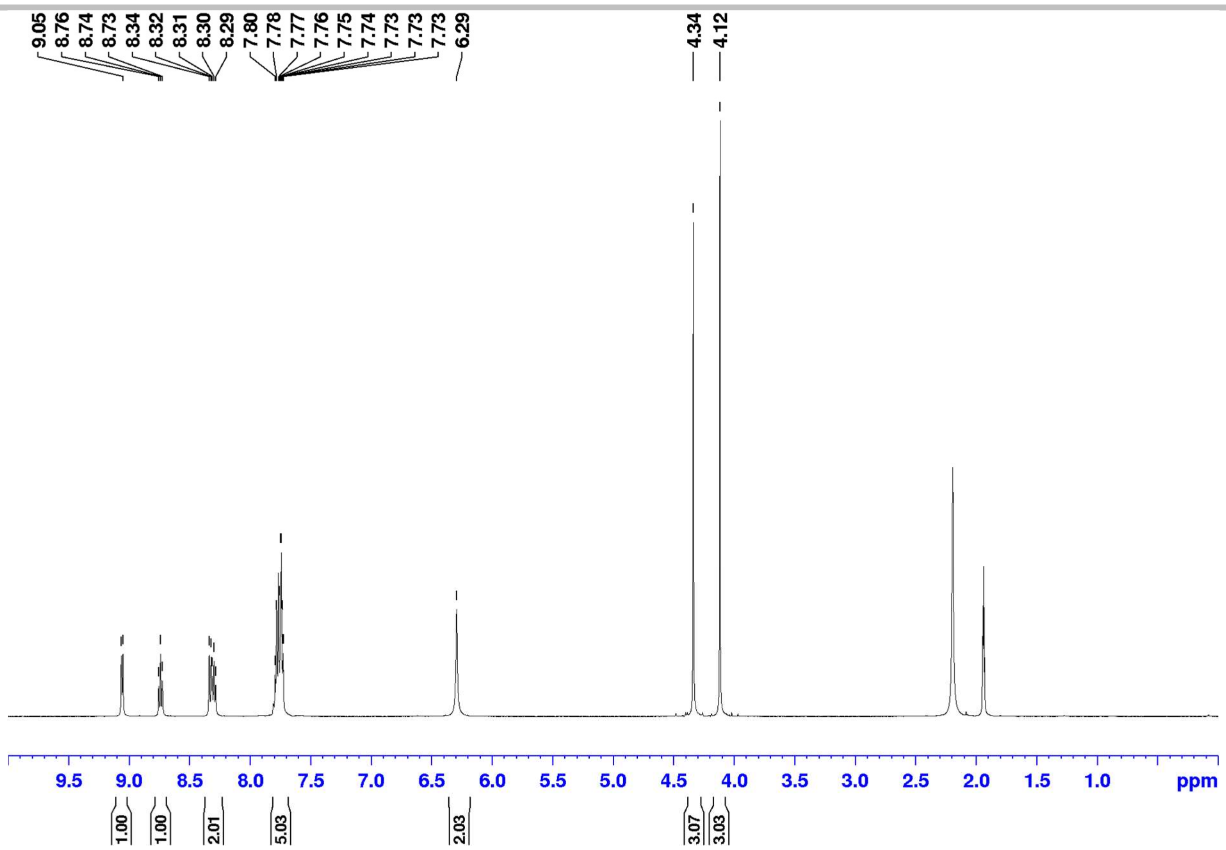


**Figure S15.** $^1$H-NMR-spectrum of **2d** ($CD_3CN$, 500 MHz).

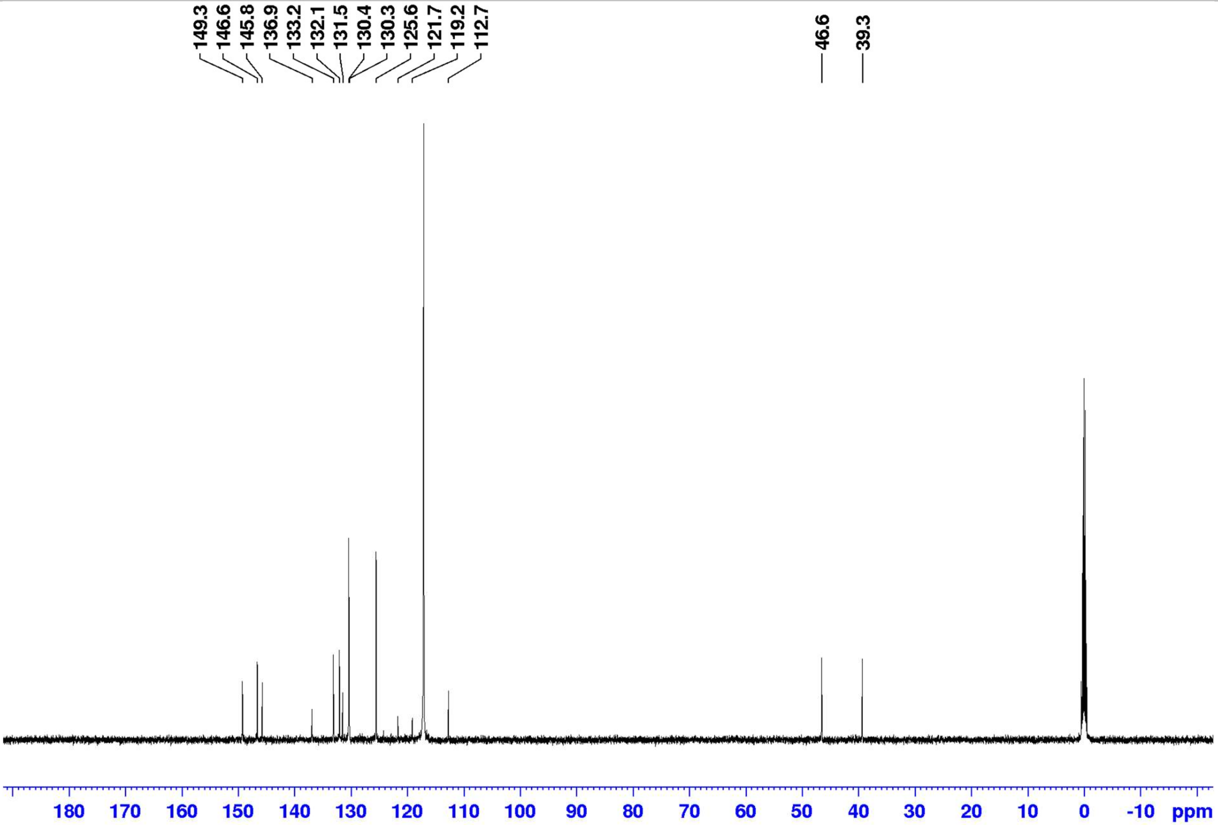


**Figure S16.** $^{13}C\{^{1}H\}$-NMR-spectrum of **2d** ($CD_3CN$, 126 MHz).

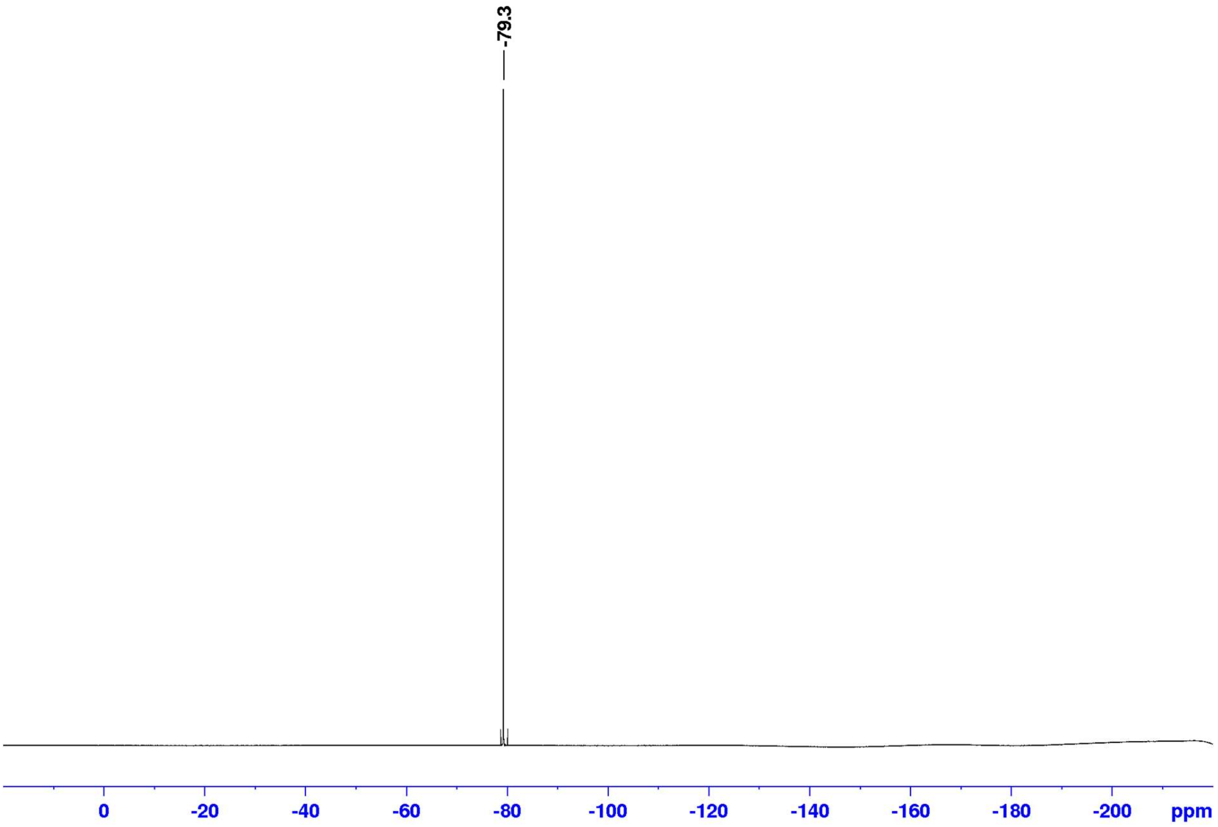


**Figure S17.** $^{19}F$-NMR-spectrum of **2d** ($CD_3CN$, 235 MHz).

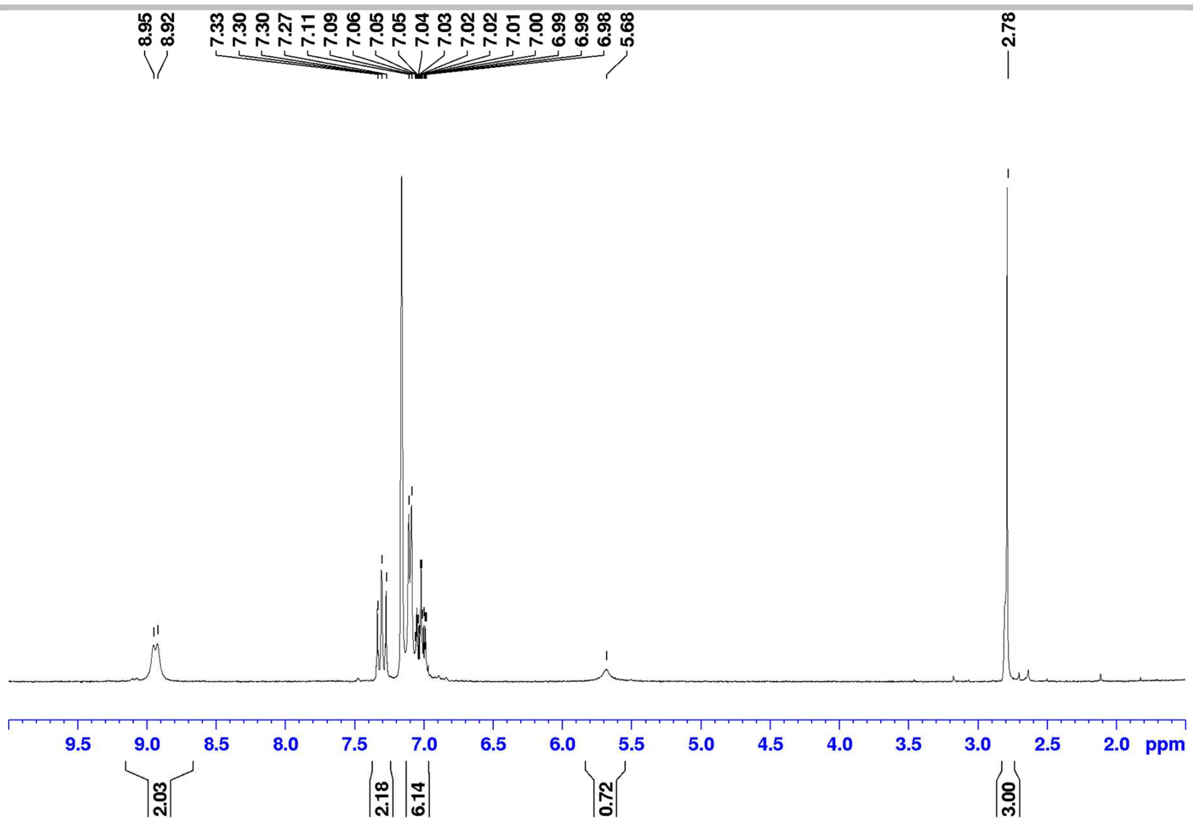

**Figure S18.** $^{1}$H-NMR-spectrum of **3a** ($C_6D_6$, 250 MHz).

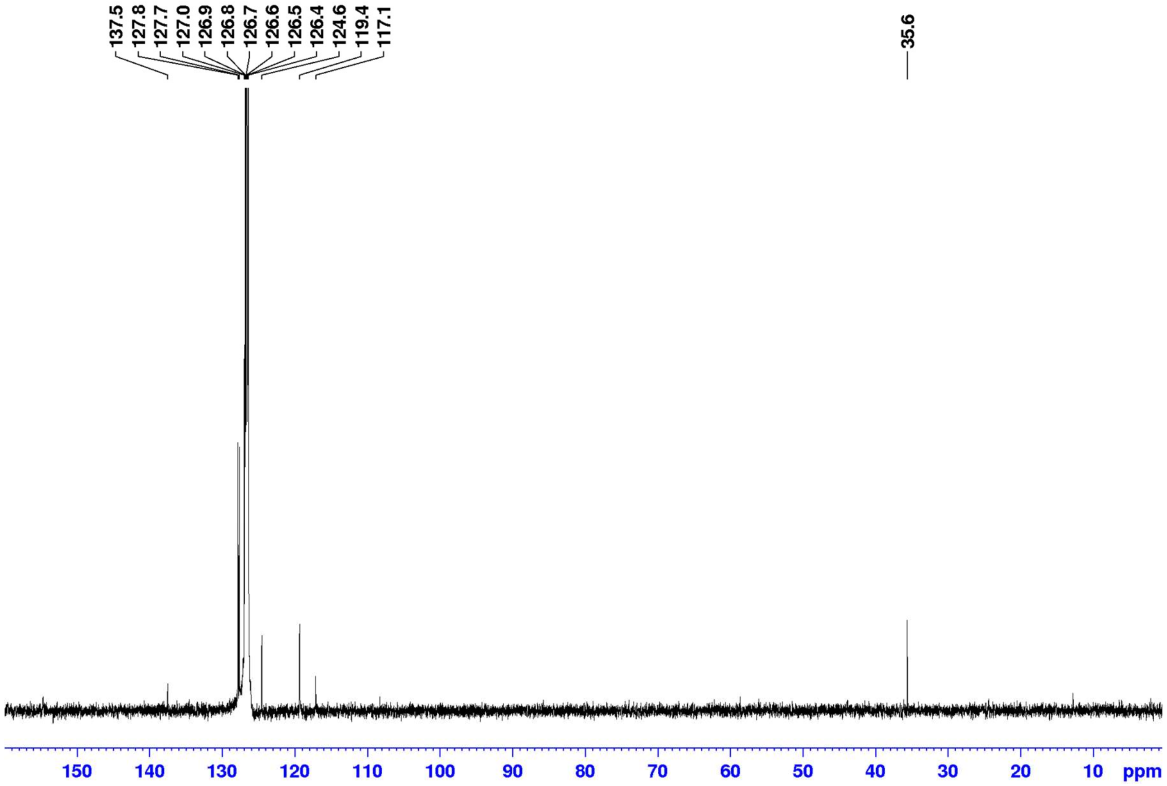

**Figure S19.** $^{13}C\{^{1}H\}$-NMR-spectrum of **3a** ($C_6D_6$, 126 MHz).

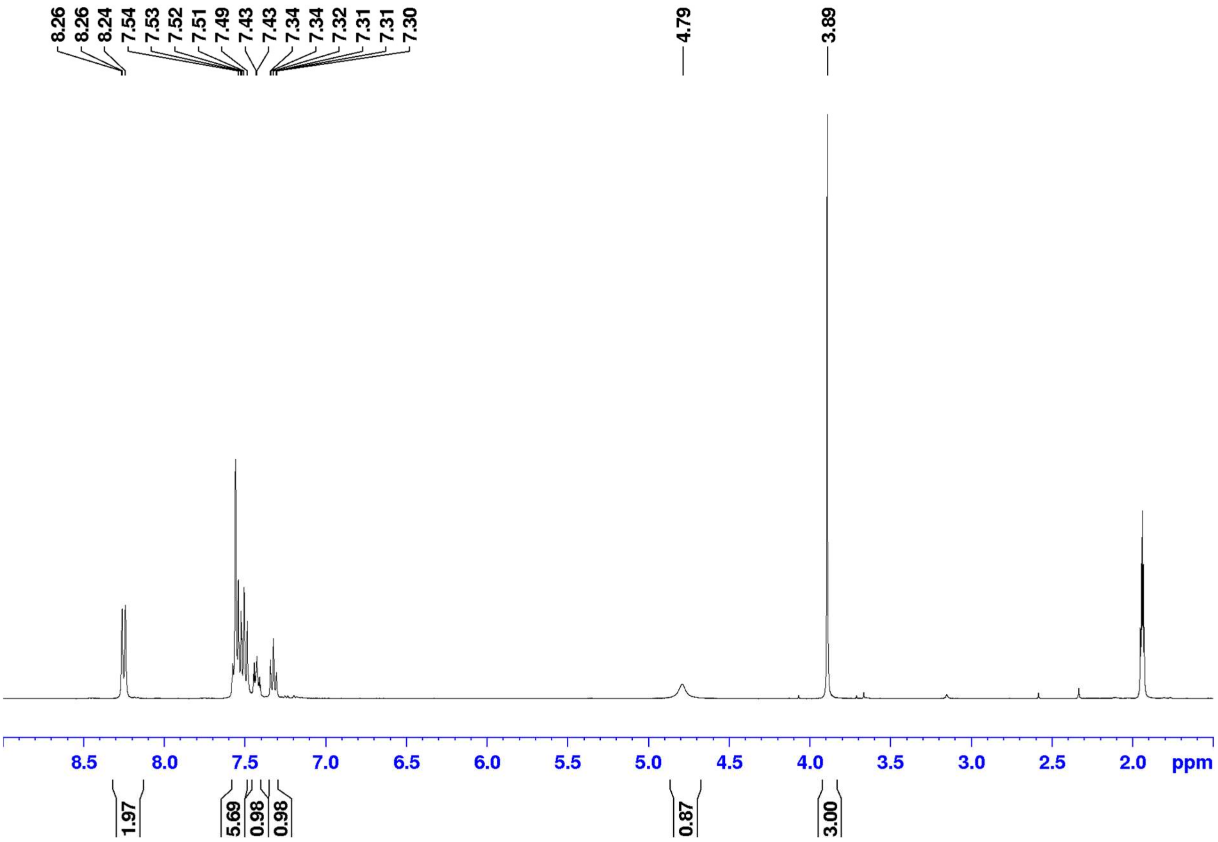


**Figure S20.** $^{1}H$-NMR-spectrum of **3a** ($CD_3CN$, 400 MHz).

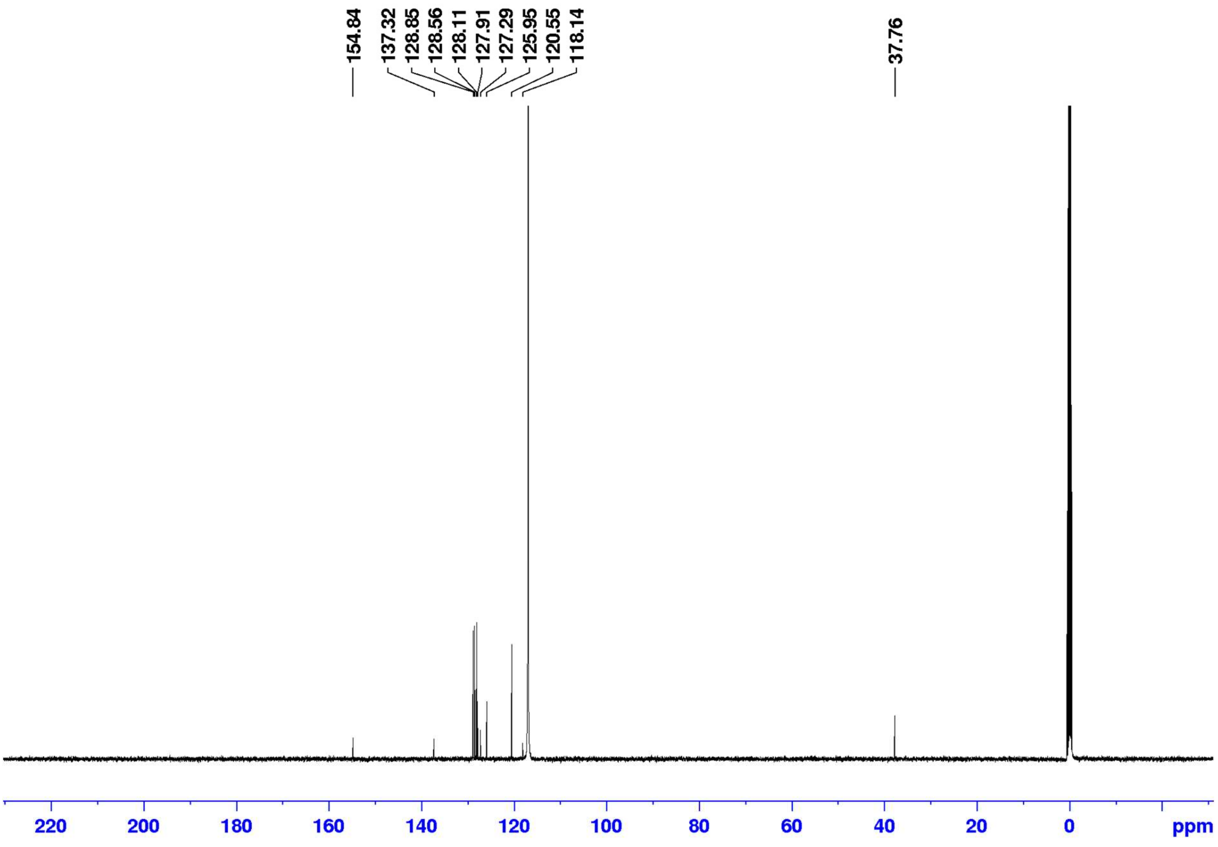


**Figure S21.** $^{13}C\{^{1}H\}$-NMR-spectrum of **3a** ($CD_3CN$, 126 MHz).

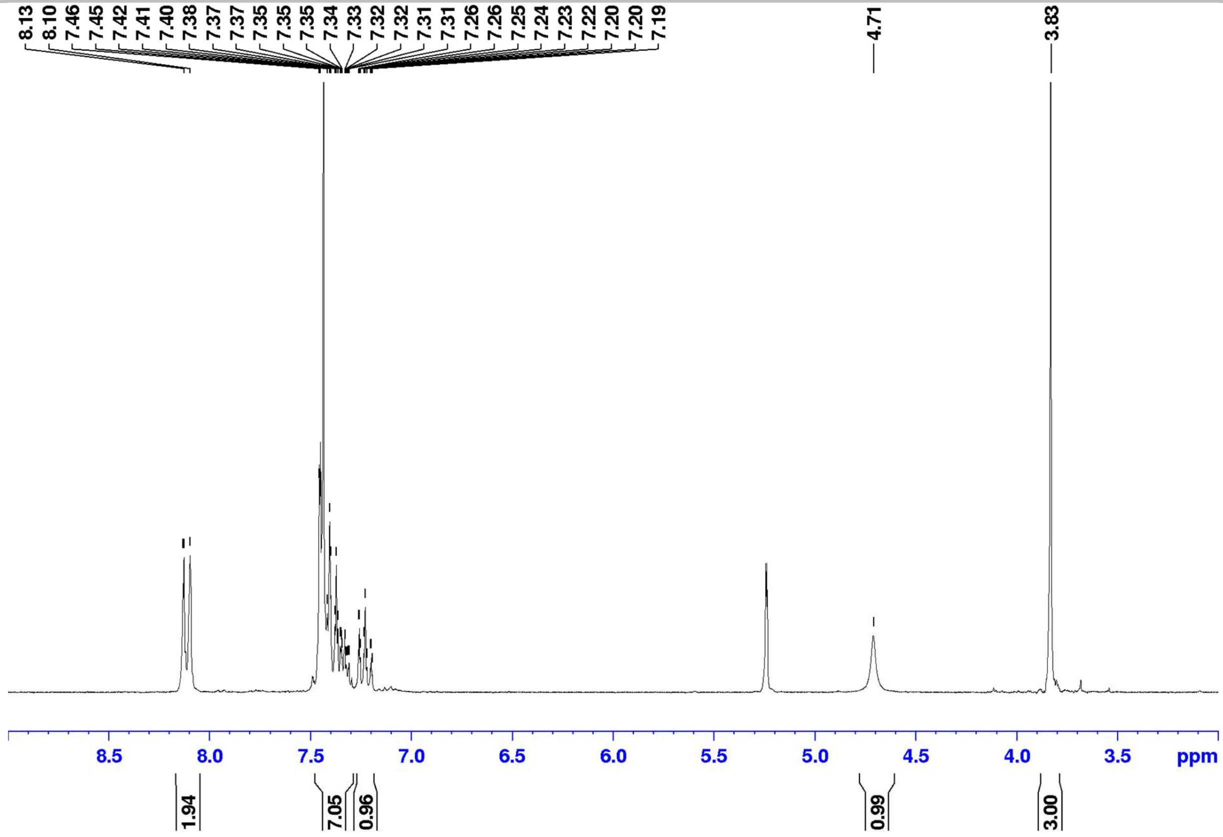


**Figure S22.** $^{1}$H-NMR-spectrum of **3a** ($CD_2Cl_2$, 250 MHz).

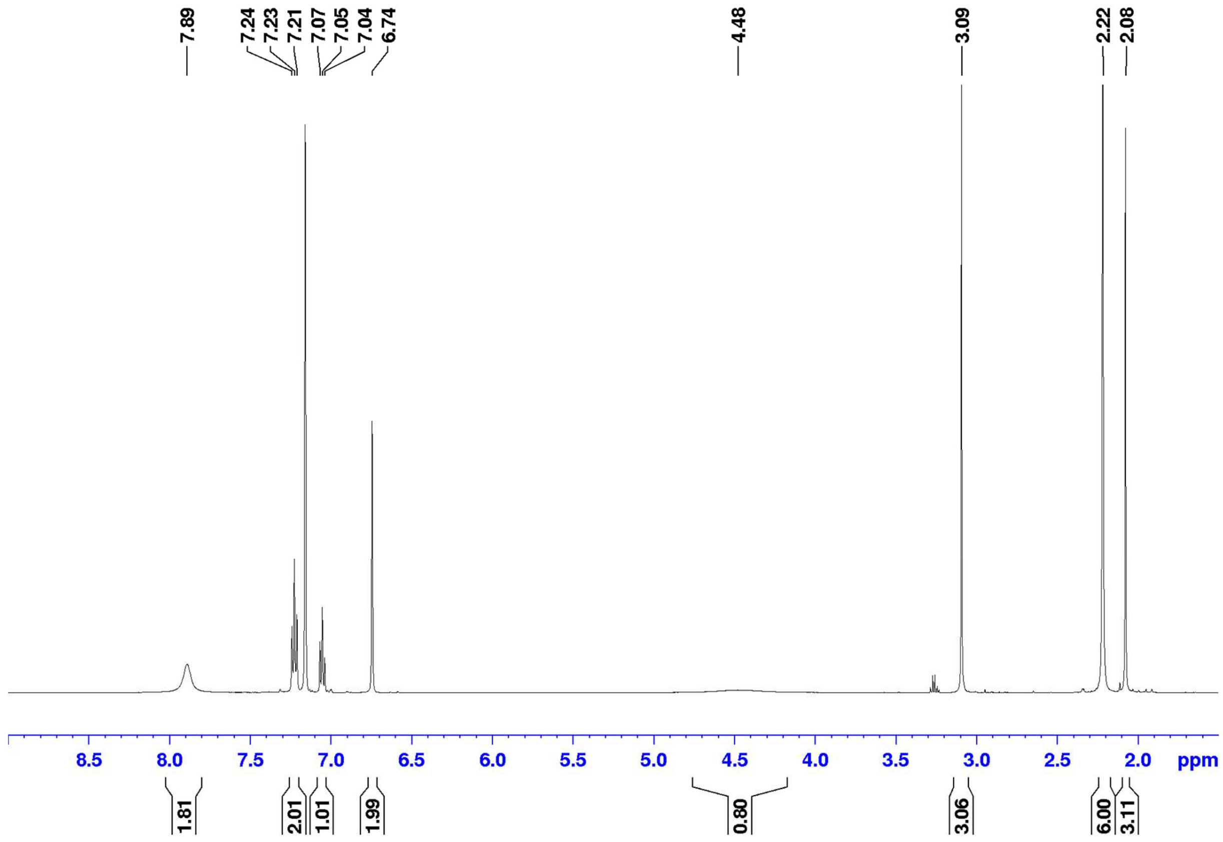


**Figure S23.** $^{1}$H-NMR-spectrum of **3b** ($C_6D_6$, 500 MHz).

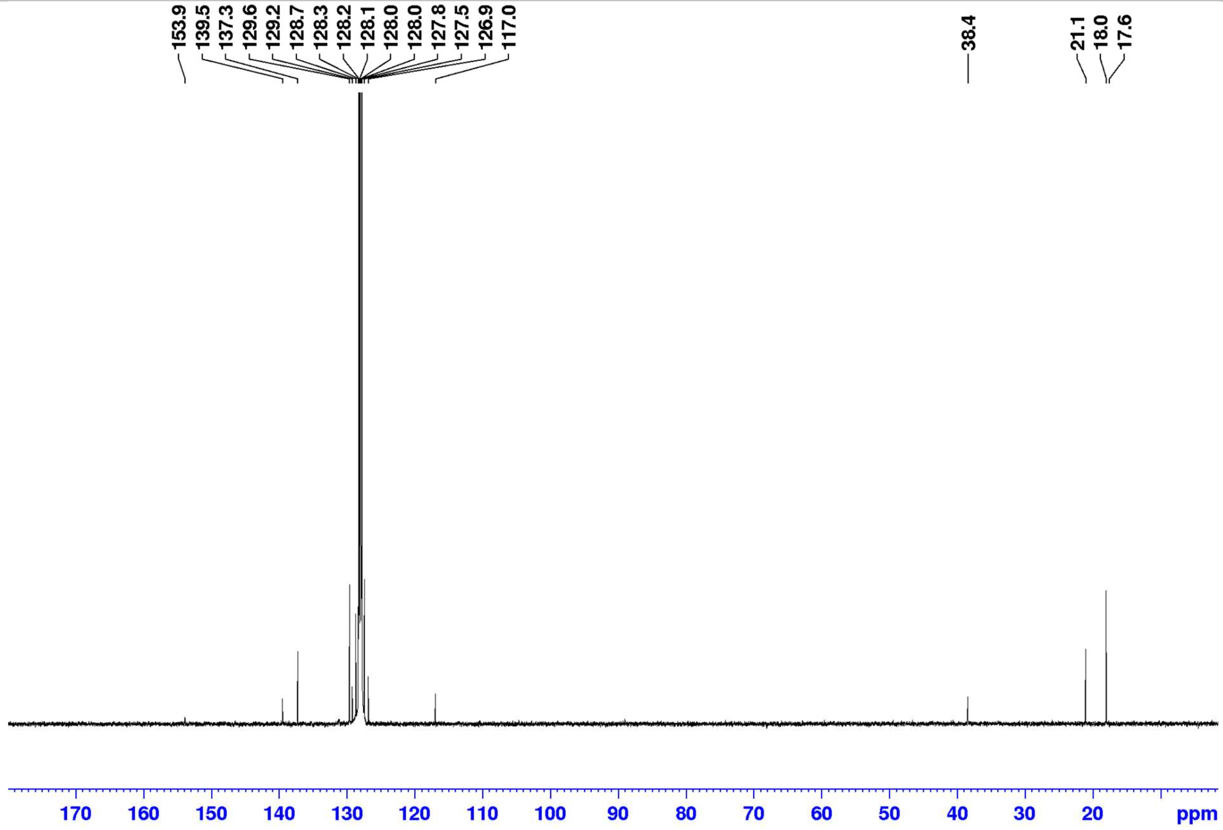


**Figure S24.** $^{13}C\{^1H\}$-NMR-spectrum of **3b** ($C_6D_6$, 126 MHz).

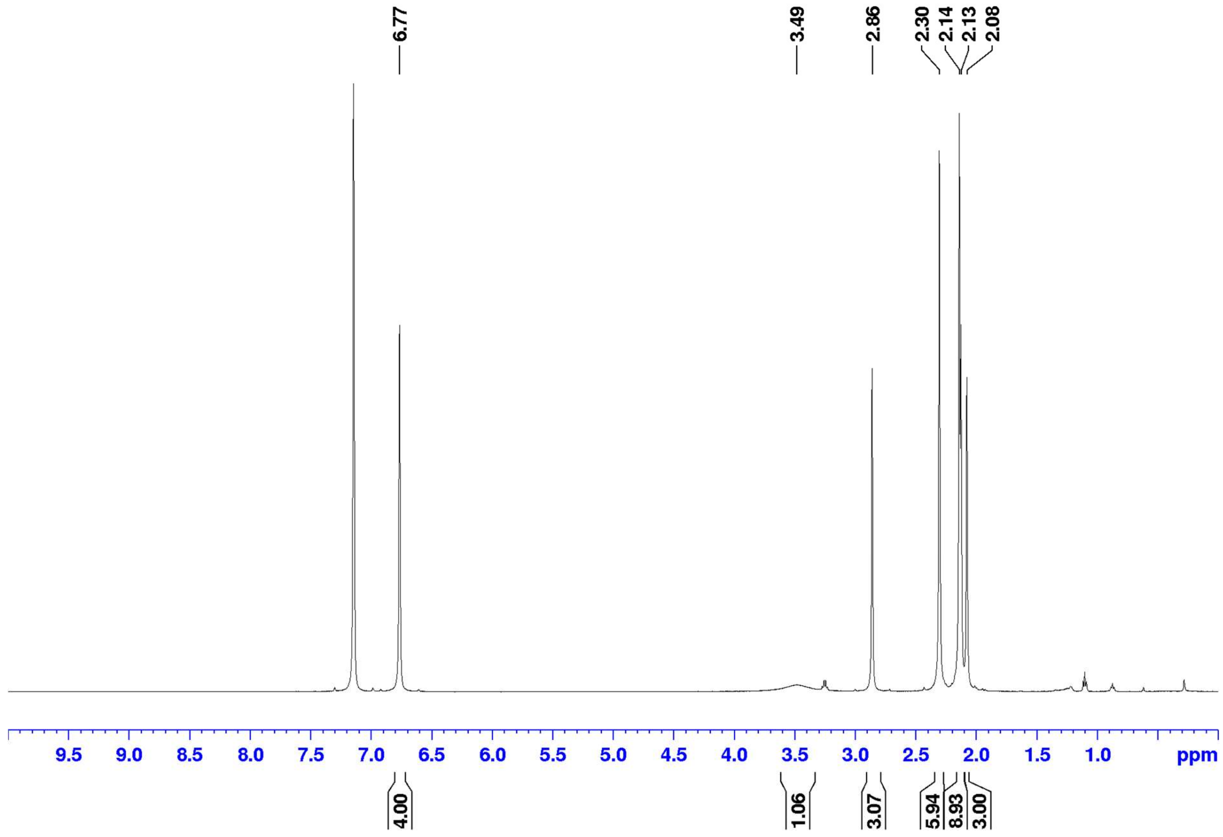


**Figure S25.** $^1H$-NMR-spectrum of **3c** ($C_6D_6$, 500 MHz).

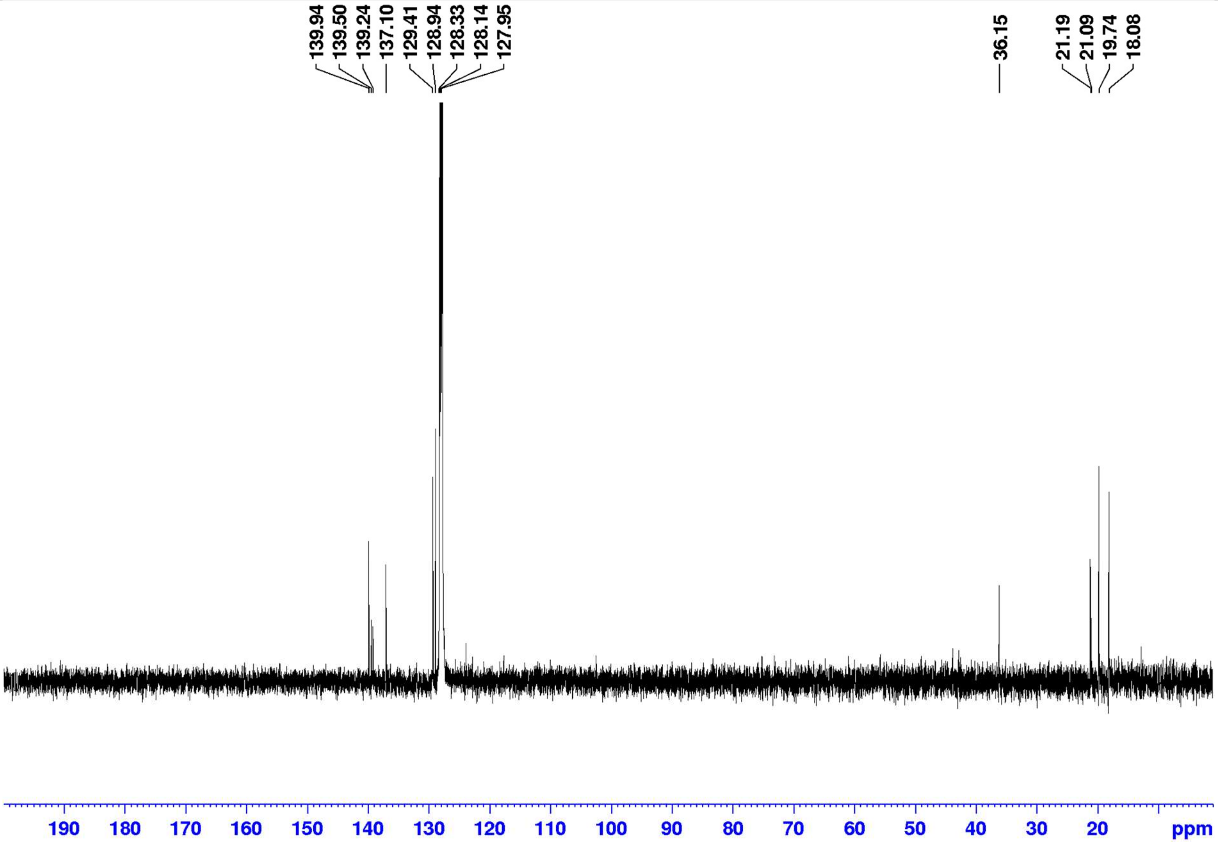


**Figure S26.** $^{13}C\{^{1}H\}$-NMR-spectrum of **3c** ($C_6D_6$, 126 MHz).

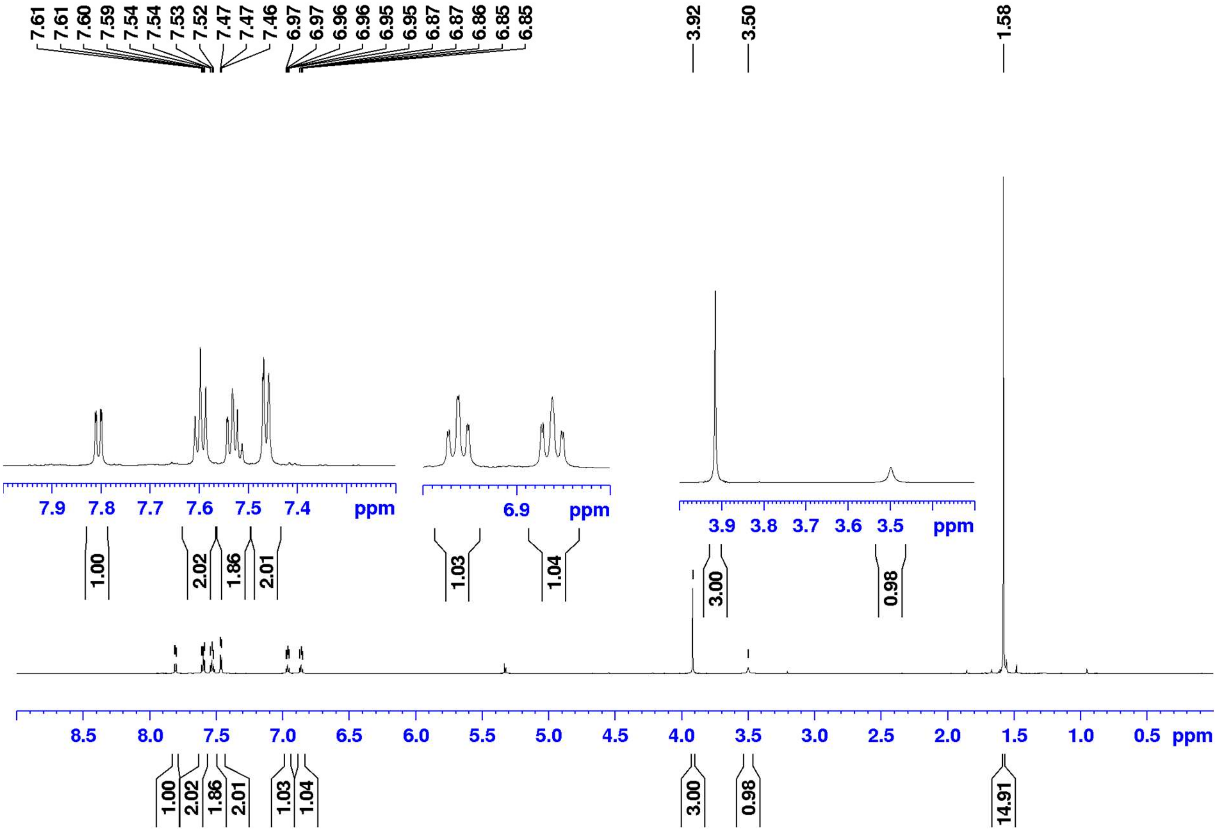


**Figure S27.** $^{1}H$-NMR-spectrum of **5a** ($CD_2Cl_2$, 700 MHz).

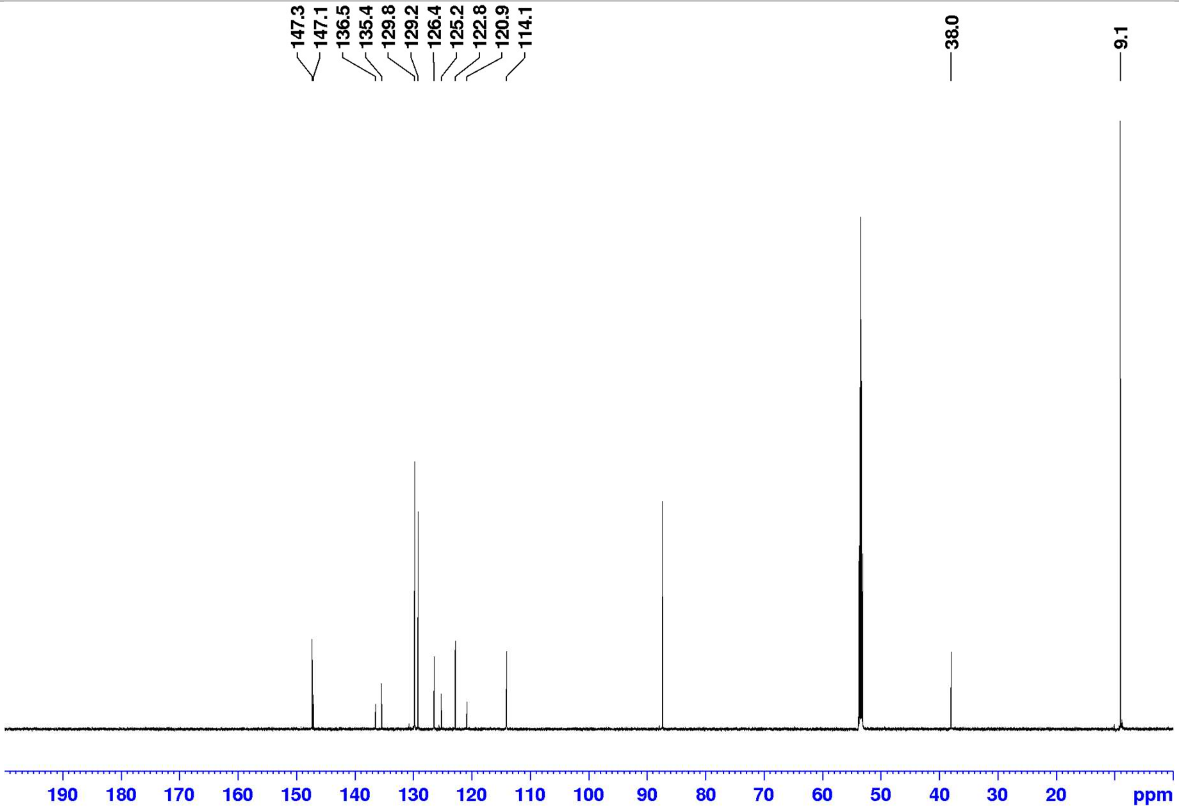


**Figure S28.** $^{13}C\{^{1}H\}$-NMR-spectrum of **5a** ($CD_2Cl_2$, 176 MHz).

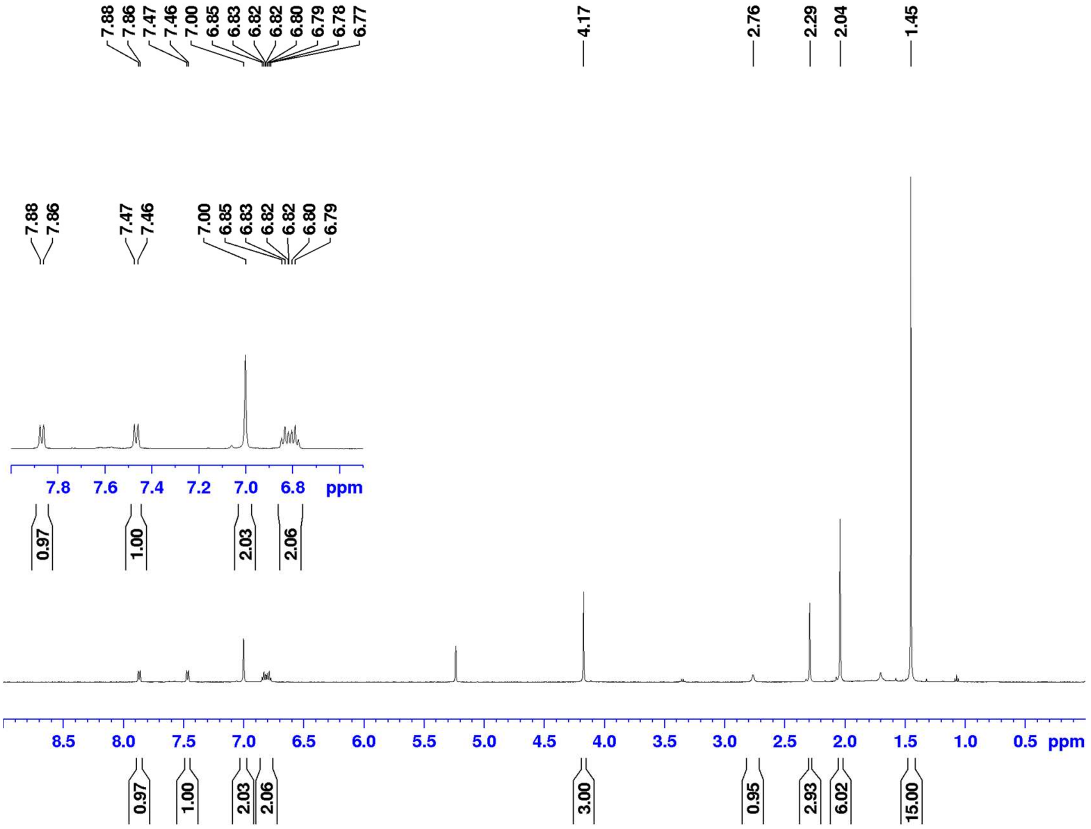


**Figure S29.** $^{1}H$-NMR-spectrum of **5b** ($CD_2Cl_2$, 500 MHz).

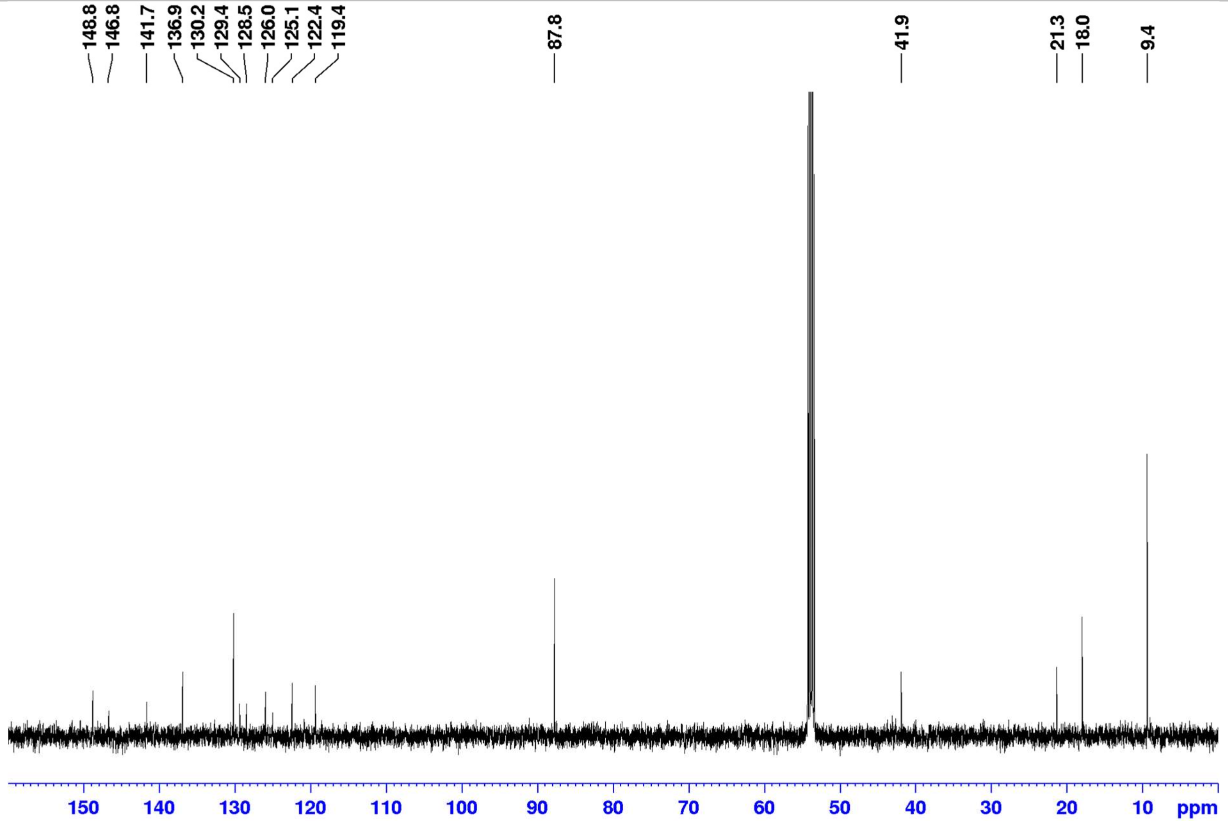


**Figure S30.** $^{13}C\{^{1}H\}$-NMR-spectrum of **5b** ($CD_2Cl_2$, 126 MHz).

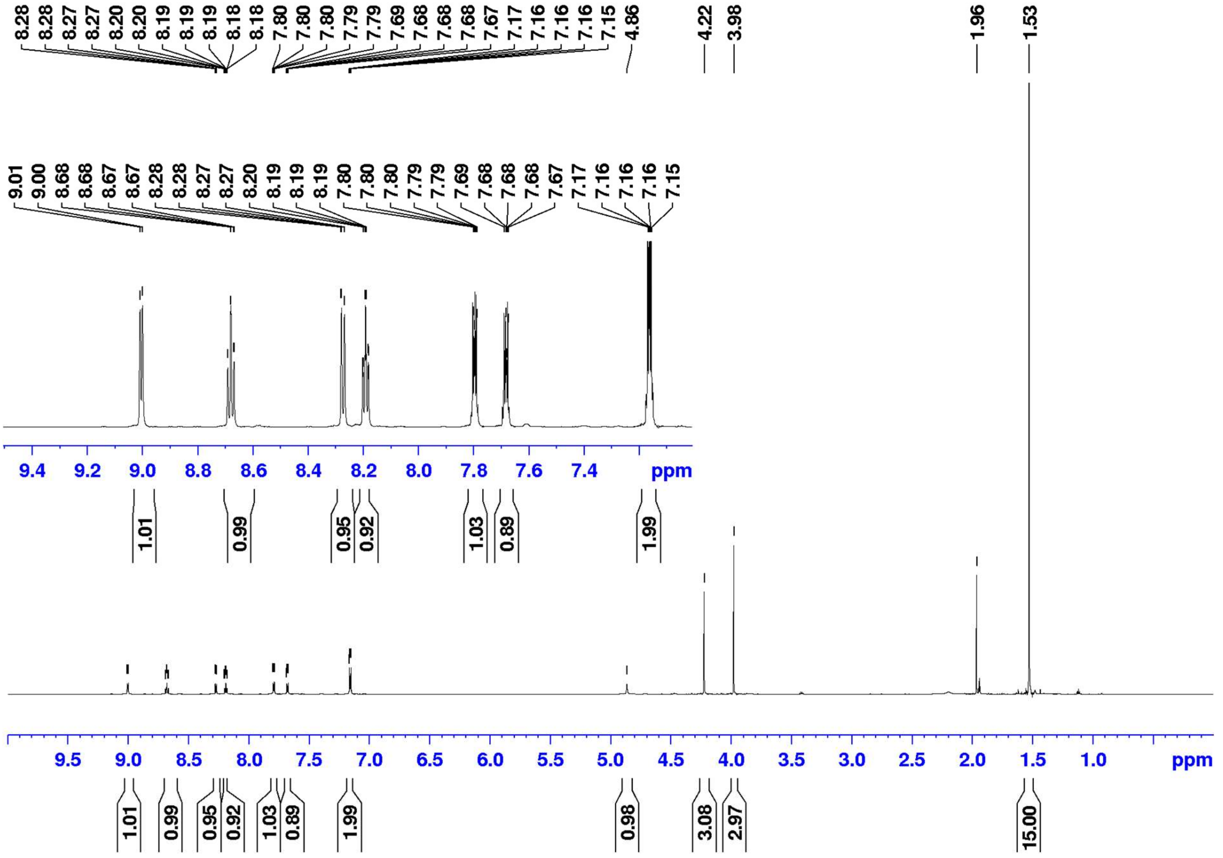


**Figure S31.** $^{1}H$-NMR-spectrum of **5d** ($CD_3CN$, 700 MHz).

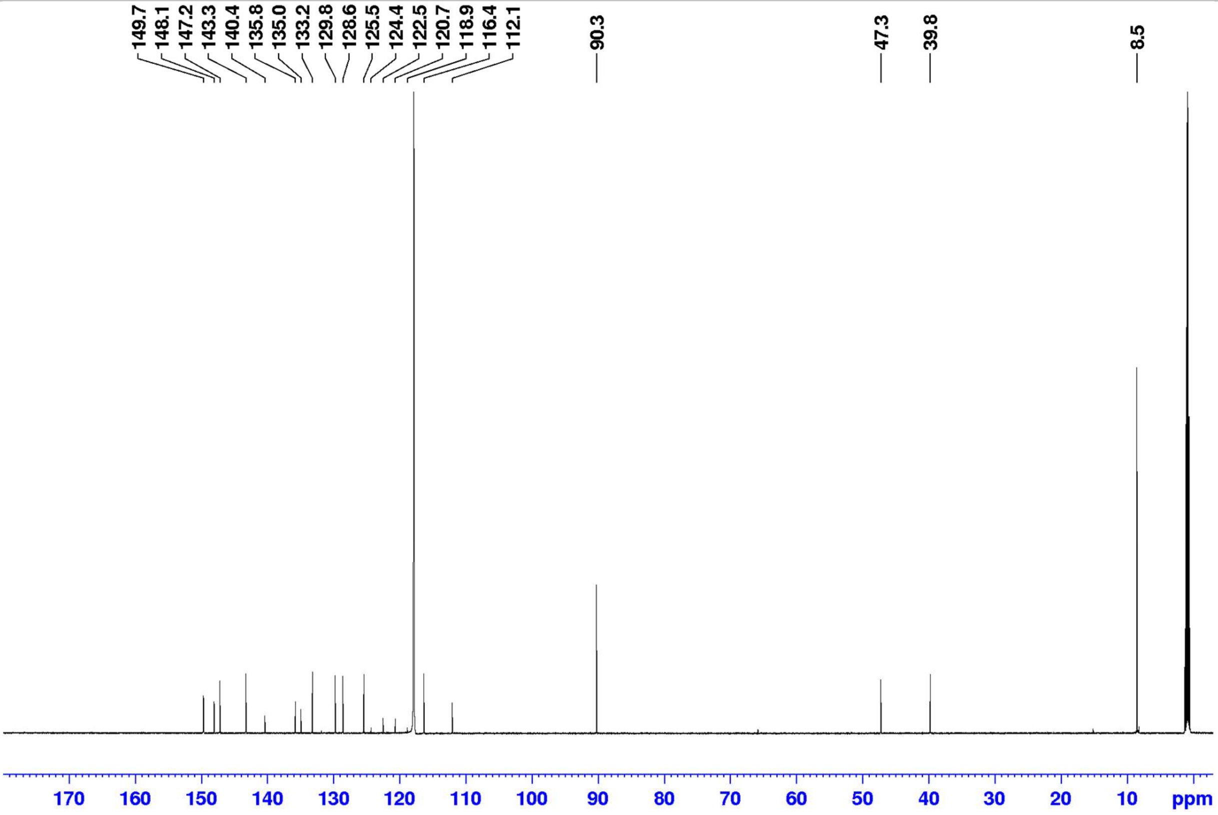


**Figure S32.** $^{13}C\{^1H\}$-NMR-spectrum of **5d** ($CD_3CN$, 176 MHz).

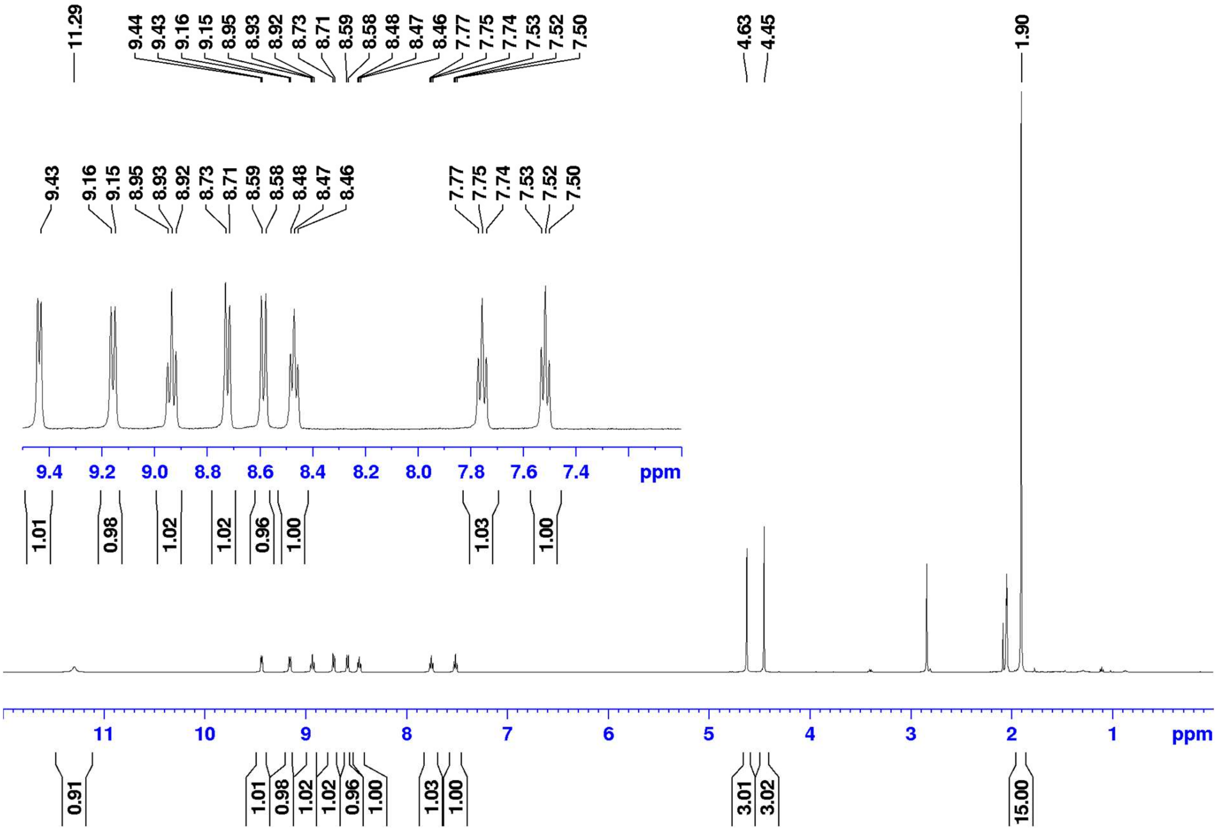


**Figure S33.** $^1H$-NMR-spectrum of **5d-acetone** (acetone-d6, 500 MHz).

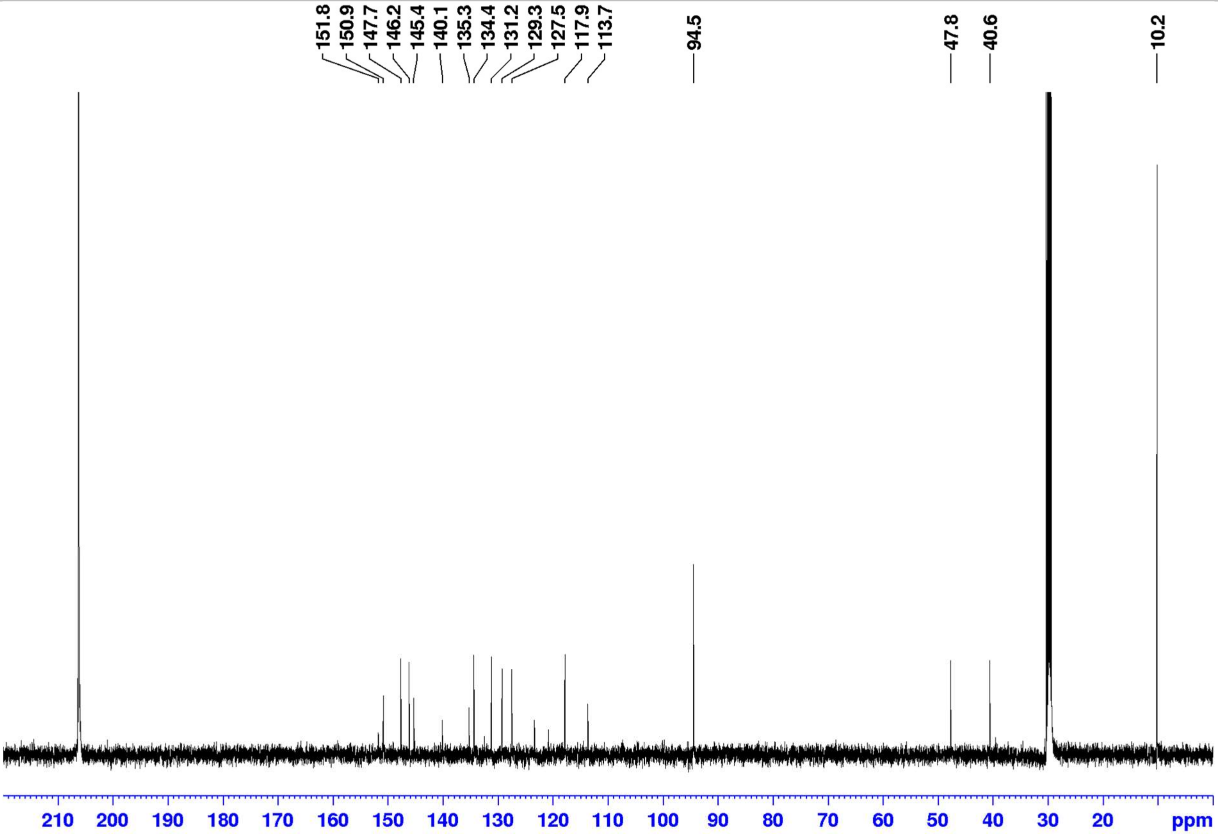


**Figure S34.** $^{13}C\{^{1}H\}$-NMR-spectrum of **5d-acetone** (acetone-d6, 126 MHz).

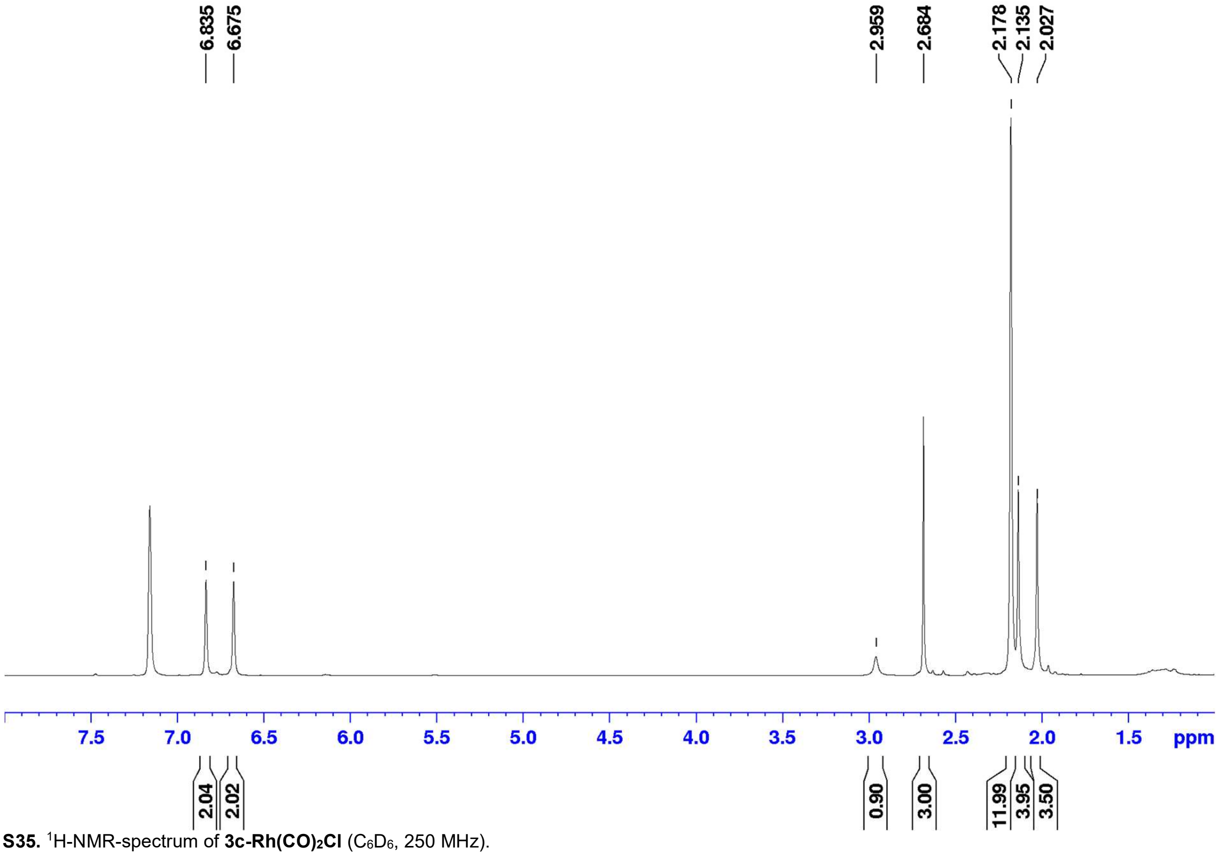


**Figure S35.** $^{1}H$-NMR-spectrum of **3c-Rh(CO)$_2$Cl** ($C_6D_6$, 250 MHz).

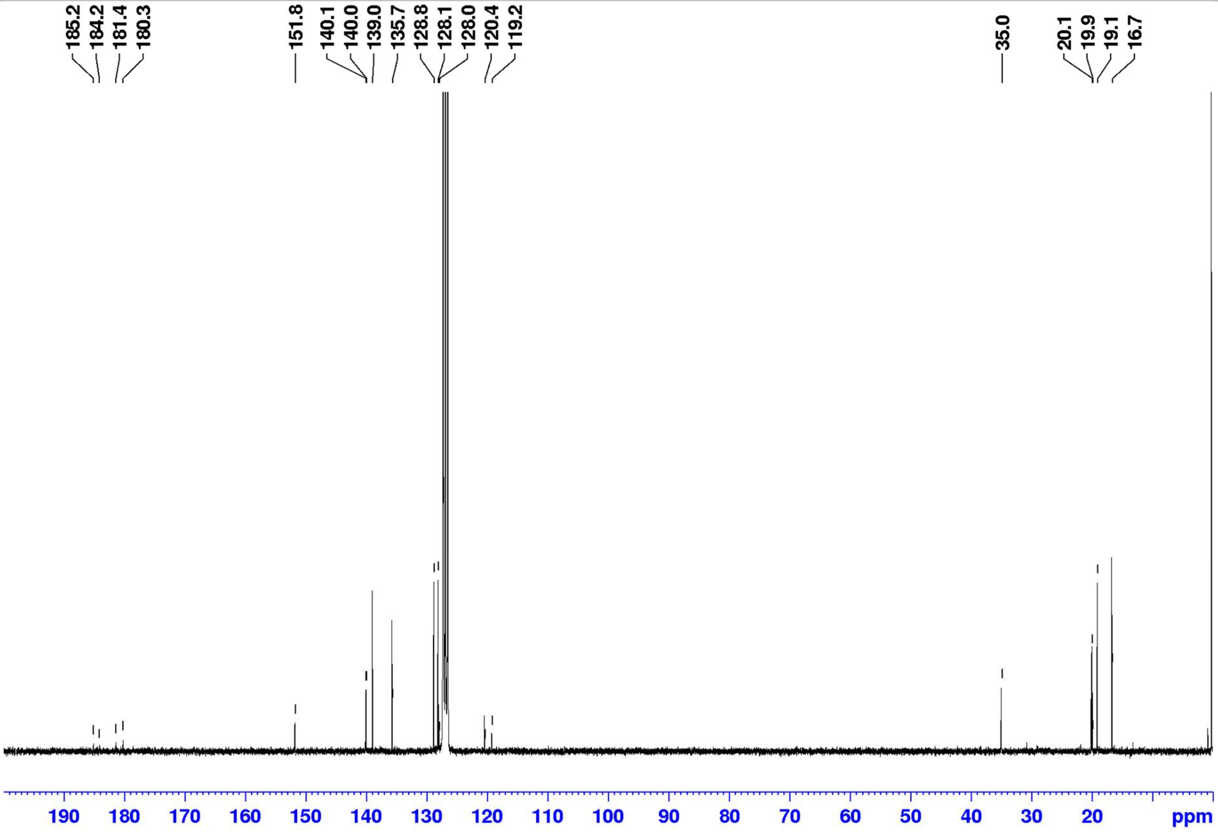


**Figure S36.** $^{13}C\{^{1}H\}$-NMR-spectrum of **3c-Rh(CO)$_2$Cl** ($C_6D_6$, 63 MHz).

4. IR-Spectra

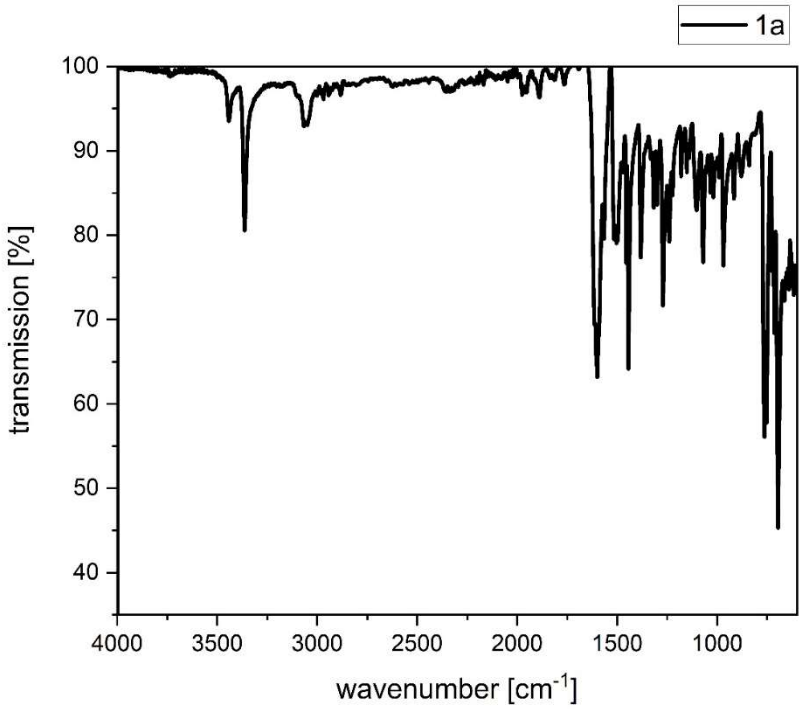


**Figure S37.**FTIR-spectrum of ***1a*** (powder ATR-unit).

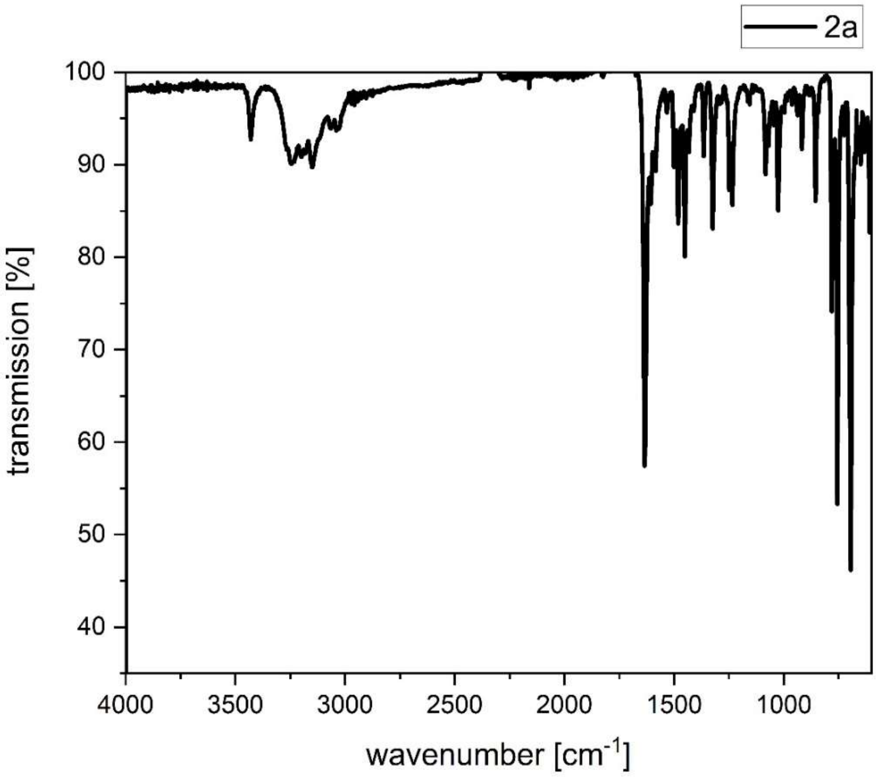


**Figure S38.** FTIR-spectrum of **2a** (powder ATR-unit).

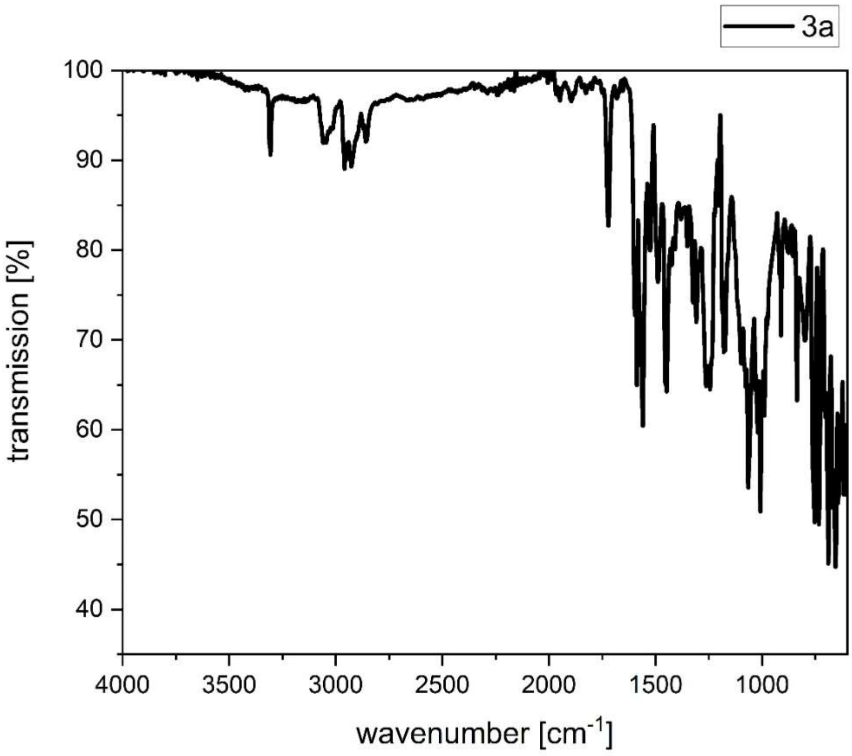


**Figure S39.**FTIR-spectrum of **3a** (powder ATR-unit).

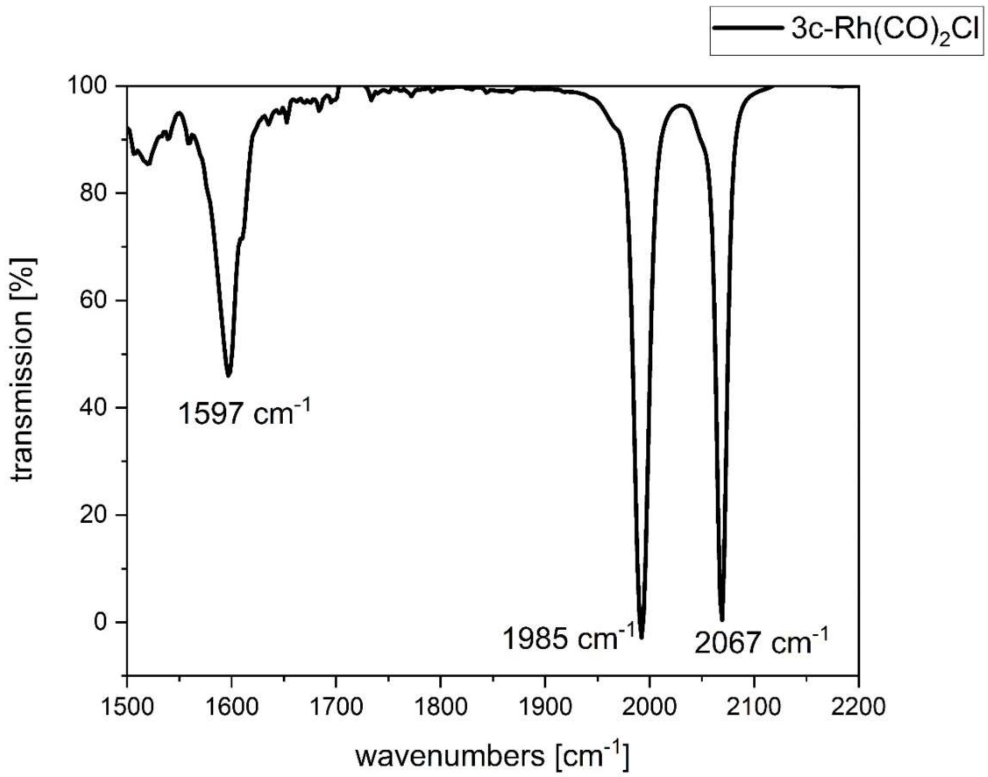


**Figure S40.** FTIR-spectrum of **3c-Rh(CO)2Cl** ($CH_2Cl_2$, $CaF_2$-windows).

$$\nu_{\text{av}} = \frac{1985\ \text{cm}^{-1} + 2067\ \text{cm}^{-1}}{2} = 2026\ \text{cm}^{-1}$$
$$TEP = 0.8001 \cdot \nu_{\text{av}} + 420.0\ \text{cm}^{-1} = 2041\ \text{cm}^{-1}$$

5. Detailed discussion of the crystallographic and spectroscopic data of 1-3

The crystallographic data shows (*Table 1*), that the relevant C1-N4 bond length is in the same range for the respective 1,4-diphenyl (**1a-3a**) and 1,4-dimesityl (**1c[31]-3c**) substituted triazole-compounds indicating that the in the manuscript aforementioned hydrogen bonding interactions have no influence on the C1-N4 bond order. A structural similarity is also observed between the precursors (**1a,c** and **2a,c[31]**) when comparing the relevant chemical shift of the N-*H* proton (*Table 1*). The imines **3a** and **3c** on the other hand show significant differences in the shift of the N-*H* proton in the $^1$H-NMR-spectra. Compared to the bisphenyl substituted imine **3a** (4.78/5.49 ppm ($CD_3CN/C_6D_6$)) the N-*H* proton in **3c** (2.33/3.48 ppm ($CD_3CN/C_6D_6$)) resonates at higher frequencies. This behaviour is attributed to the intermolecular N4-H4···N4'-H4'··· interactions found in **3a**.

Compared to the triazolium salt **2a**, the $^1$H-NMR-spectrum of **3a** shows one signal with an integral of 2 (relative to $\delta^1$H(N-C$H_3$)) shifted to significantly higher frequencies while two distinct signals with a relative integral of 1 in each case are shifted towards lower frequencies (Figure S41). This behaviour is explained by the previously illustrated intramolecular interactions of the respective ortho-hydrogen atoms in the phenyl moieties with the exocyclic N-fragment. Breaking of the $C_2$-symmetry in the phenyl substituents is a direct result of these interactions. Hence the downfield-shifted signals are assigned to the C10/C8-*H*8A/*H*10···N4 protons. The upfield-shifted signals are assigned accordingly to the remaining set of ortho-hydrogen atoms, which are not facing the exocyclic N-fragment.

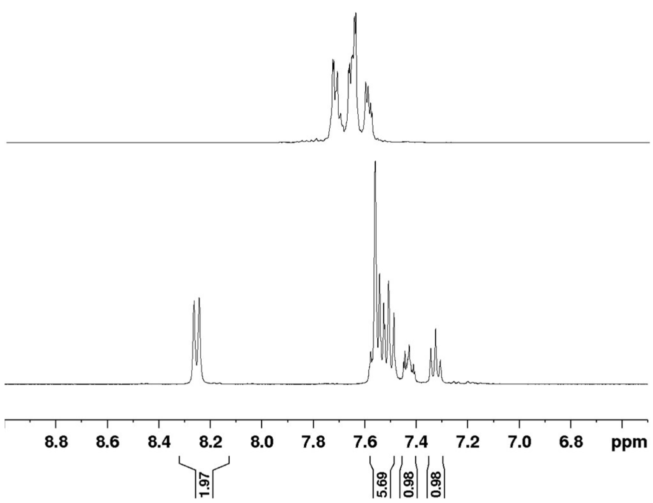


**Figure S41.** $^1$H-NMR-spectra ($CD_3CN$, 400 MHz). Top **2a**. Bottom: **3a**.

6. Complexation reactions

6.1 Preparation of **3a-CO₂** solutions

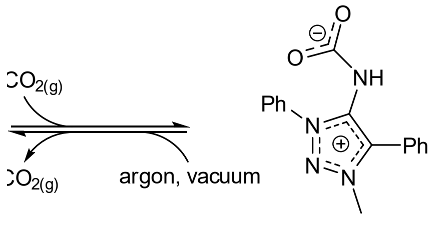


**3a-CO₂**

The ligand **3a** (10 mg, 0.04 mmol, 1 eq) was submitted into an oven-dried Schlenk-flask. Under a flow of argon the ligand was dissolved in $CD_3CN$ (1.5 mL) and then transferred into two NMR-tubes (0.5 mL respectively). $^1$H and $^{13}$C-NMR-measurements were conducted with the first sample. The second sample was gassed with a mild flow of $CO_2$-Gas for 10 minutes (0.5 bar) until the solution was colourless. $^1$H and $^{13}$C-NMR-spectra were recorded with this sample:

**$^1$H-NMR** (400 MHz, $CD_3CN$) δ: 7.76 (m, Aryl-*H*, 2H), 7.59-7.47 (m, Aryl-*H*, 8H), 3.96 (s, Trz-C*H*₃, 3H).

**$^{13}$C-NMR** (101 MHz, $CD_3CN$) δ: 134.8, 130.2, 129.5, 129.4, 129.0, 124.2, 123.53, 38.4 (N-*C*H₃).

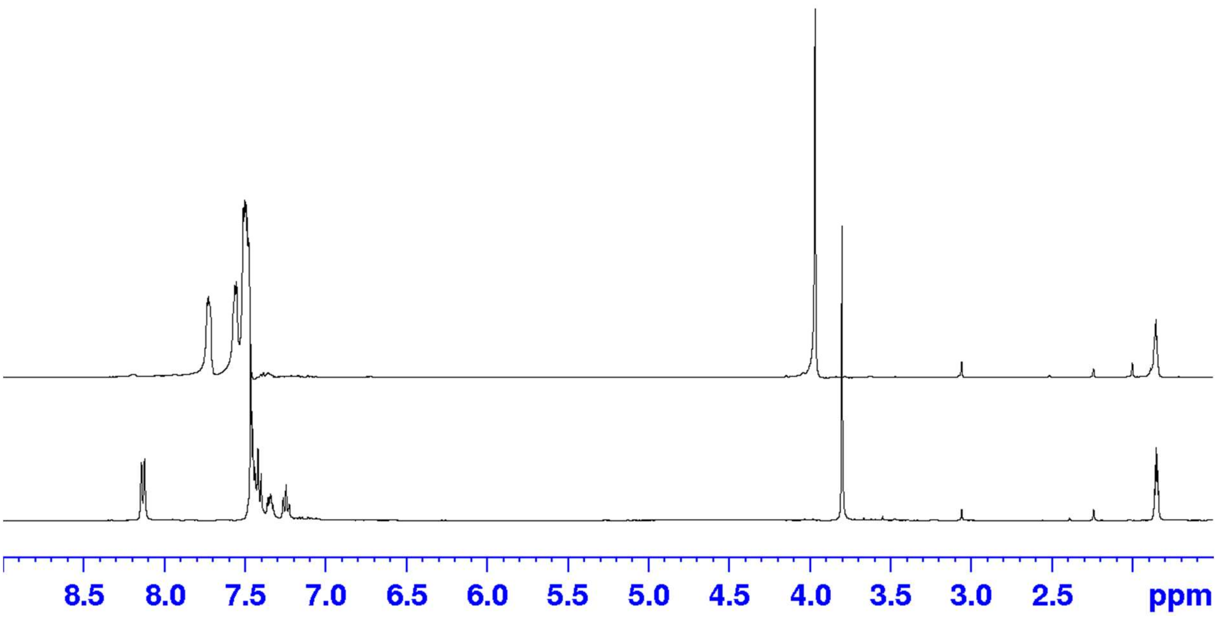


**Figure S42.** $^{1}$H-NMR-spectrum of ($CD_3CN$, 400 MHz). Bottom: **3a**. Top: **3a** after gassing with $CO_2$-gas.

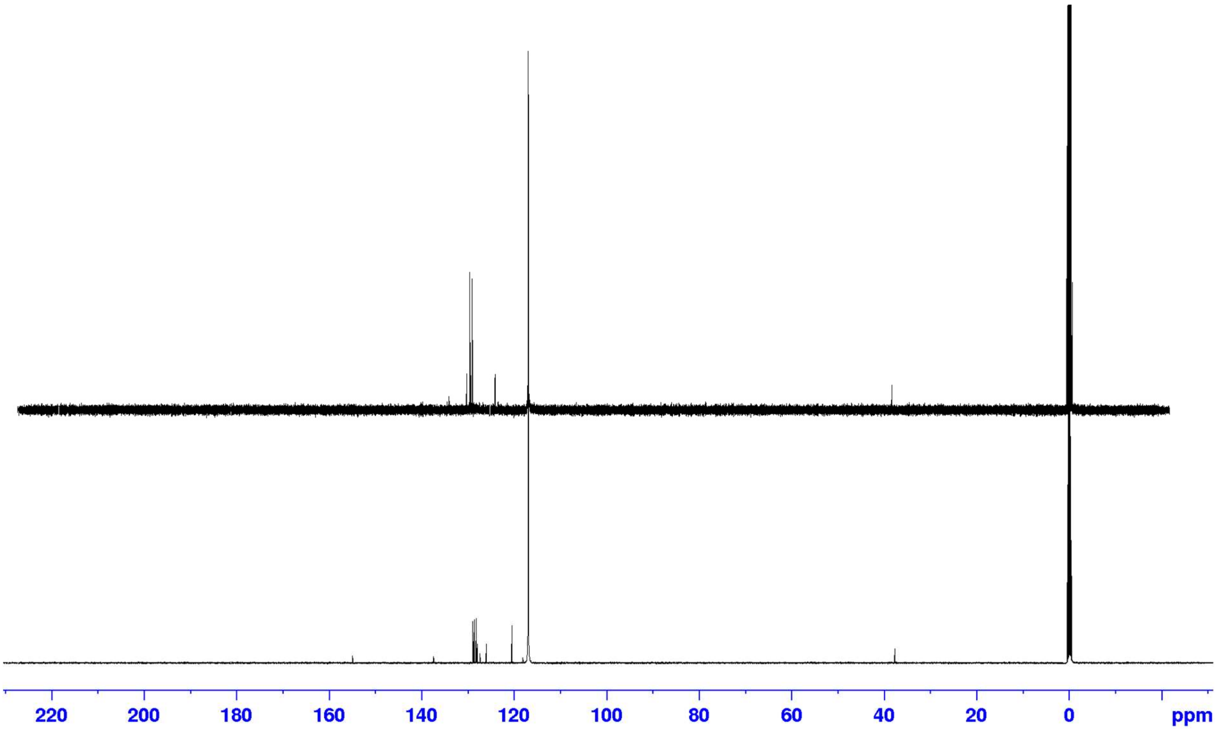


**Figure S43.** $^{13}C\{^1H\}$-NMR-spectrum ($CD_3CN$). Bottom: **3a** (126 MHz). Top: **3a** after gassing with $CO_2$-gas (101 MHz).

The sample was then flushed with argon-gas for 10 minutes resulting in a bright yellow coloured solution. $^1H$ and $^{13}C$-NMR-spectra were recorded again with this sample yielding the native spectrum of the first sample.

The ligand **3a** (20 mg, 0.08 mmol, 1 eq) was submitted into an oven-dried Schlenk-flask. Under a flow of argon the ligand was dissolved in a mixture of toluene (3 mL) and *n*-pentane (3 mL). $CO_2$-gas (1 bar) was bubbled through the solution for 1 hour. The formed precipitated was separated from the solution by transfer onto a frit via $CO_2$-pressure and Teflon tubing. The filter cake was washed *n*-pentane (2x 3 mL) and then dried by a mild flow of $CO_2$-Gas for 1 hour. IR-measurements of the solid with an ATR-unit were conducted immediately.

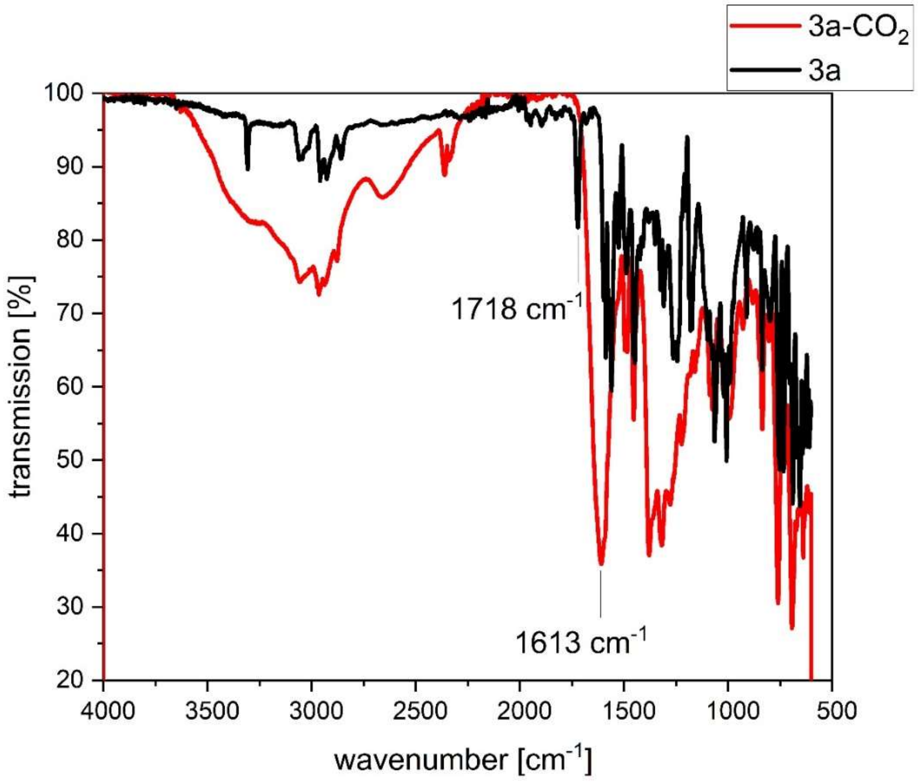


**Figure S44.** FTIR-spectrum of **3a** and **3a-CO₂** (powder ATR-unit).

### 6.2 Preparation of 3a-B($C_6F_5$)$_3$

Synthesis of **3a-B($C_6F_5$)$_3$**

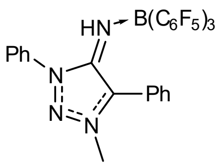


**3a-B($C_6F_5$)$_3$**

The ligand **3a** (16 mg, 0.06 mmol, 1 eq) and B($C_6F_5$)$_3$ (33 mg, 0.06 mmol, 1 eq) were submitted into an oven-dried Schlenk-flask and cooled to 0 °C. $C_6D_6$ (1 mL) was added under a flow of argon and the solution was stirred for 10 minutes. The solution was allowed to warm to room temperature and was stirred over night at room temperature. The resulting colourless solution was investigated.

Single crystals suitable for X-ray diffractometry were obtained by slow diffusion of hexane into a solution of **3a-B($C_6F_5$)$_3$** in toluene at room temperature of the course of several days.

**$^1$H-NMR** (400 MHz, $C_6D_6$) δ: 7.02-6.83 (m, Phenyl-*H*, 6H), 6.72-6.63 (m, Phenyl-*H*, 2H), 4.93 (s, Trz=N(*H*)-BAryl$_3$, 1H), 2.43 (s, N-C*H*$_3$, 3H).
One set of signals can not be assigned as they collapse with the residual solvent signal.

**$^{11}$B-NMR** (128 MHz, $C_6D_6$) δ: -9.1 (s).

**$^{13}$C-NMR** (101 MHz, $C_6D_6$) δ: 146.9, 132.1, 131.4, 130.3, 130.1, 129.0, 128.5, 125.2, 123.7, 122.2, 37.1.

**$^{13}$C-NMR** (176.1 MHz, $C_6D_6$) δ: 168.9, 147.5 (dm, $^1J_{CF}$ = 241 Hz), 138.6 (dm, $^1J_{CF}$ = 248 Hz), 136.2 (ddd, $^1J_{CF}$ = 248 Hz, $J$ = 20 Hz, $J$ = 12 Hz).

**$^{19}$F-NMR** (376 MHz, $C_6D_6$) δ: -134.2 (d, $^3J_{FF}$ = 20 Hz), -159.3 (t, $^3J_{FF}$ = 21 Hz), -164.5 (dt, $^3J_{FF}$ = 22 Hz, $^4J_{FF}$ = 8 Hz).

**MS (ESI):** m/z = 251.13 [**PhPh=NH**+H]$^+$, 761.10 [M-H]$^-$ 785.10 [M+Na]$^+$.

**HRMS (ESI):** calc. ($C_{33}H_{14}BF_{15}N_4$), m/z = 761.1005 [M-H]$^-$, 785.0970 [M+Na]$^+$, found m/z = 761.1003 [M-H]$^-$, 785.0964 [M+Na]$^+$.

IR-measurements were conducted of the resulting residue after removal of the solvent under reduced pressure.

**FTIR** (ATR, solid) $\tilde{\nu}$: 1644 ($C_{Trz}$-$N_{exo}$).

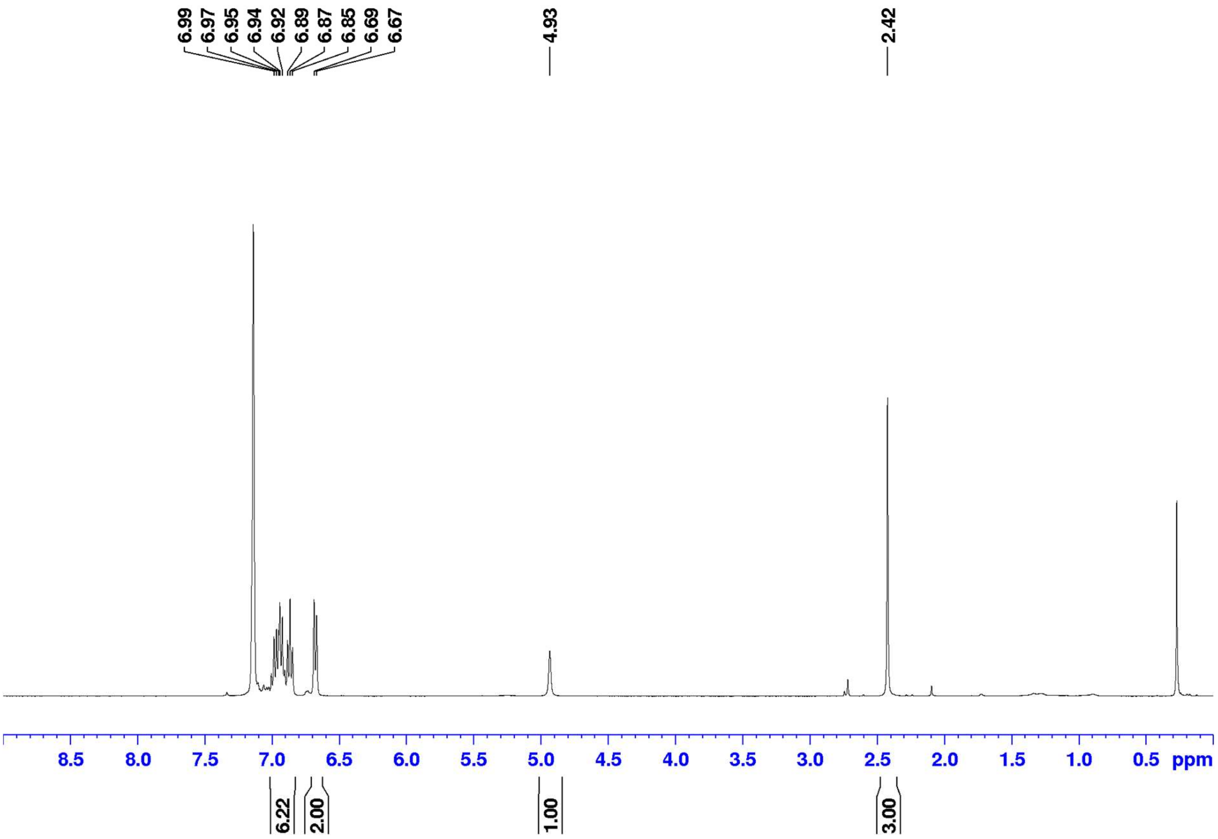


**Figure S45.** $^1$H-NMR-spectrum of **3a-B($C_6F_5$)$_3$** ($C_6D_6$, 400 MHz).

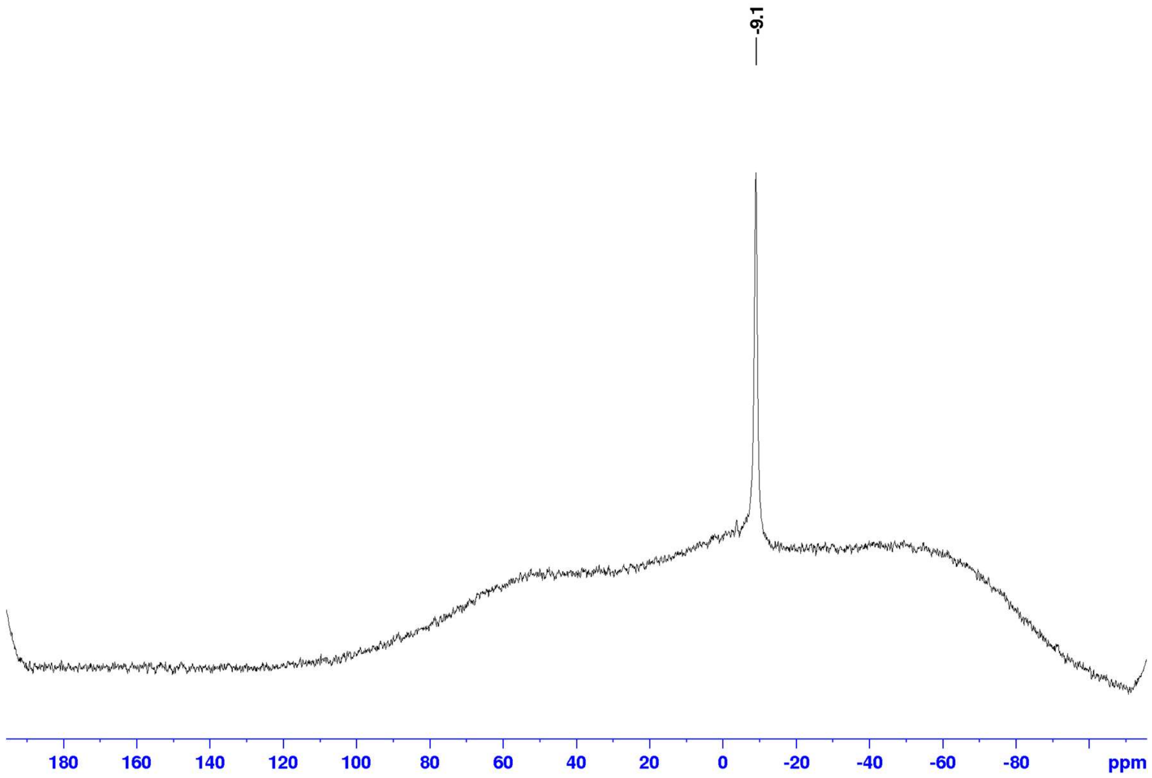


**Figure S46.** $^{11}B\{^1H\}$-NMR-spectrum of **3a-B(C$_6$F$_5$)$_3$** (C$_6$D$_6$, 80 MHz).

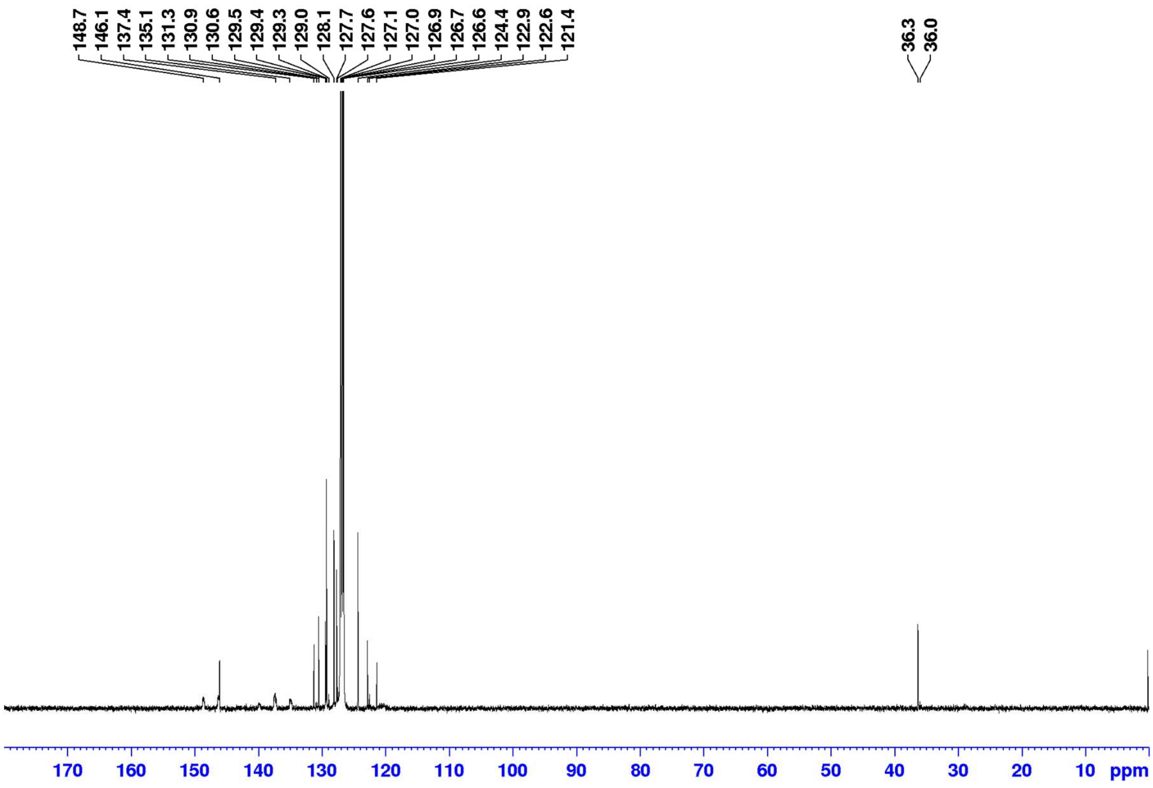


**Figure S47.** $^{13}C\{^1H\}$-NMR-spectrum of **3a-B(C$_6$F$_5$)$_3$** (C$_6$D$_6$, 101 MHz).

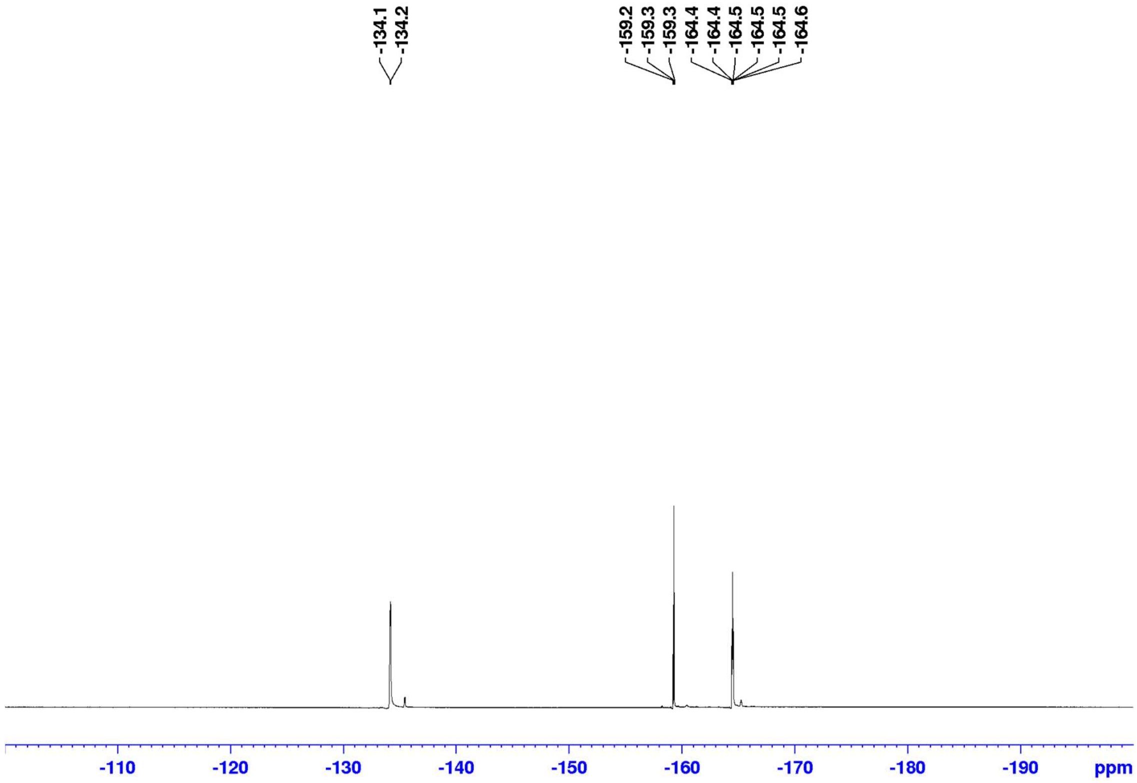


**Figure S48.** $^{19}F$-NMR-spectrum of **3a-B(C$_6$F$_5$)$_3$** ($C_6D_6$, 376 MHz).

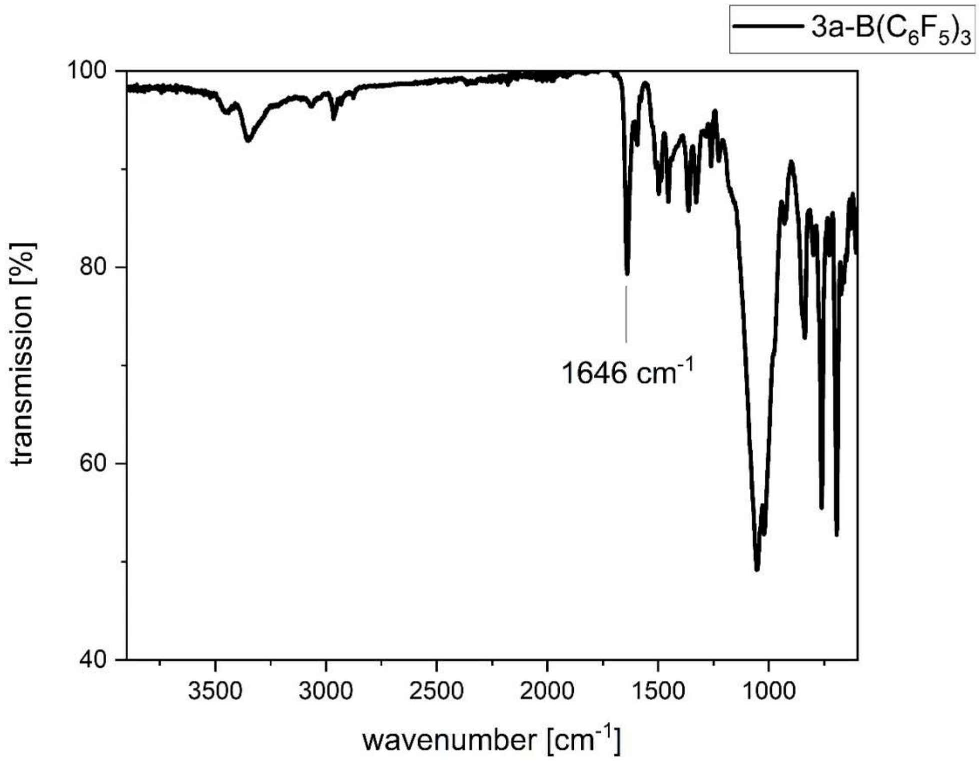


***Figure S49.*** FTIR-spectrum of **3a-B(C$_6$F$_5$)$_3$** (powder ATR-unit).

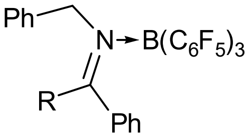


**6a** (R = Ph)
**6b** (R = Me)
**6c** (R = H)

**Figure S50.** Structure of reported acyclic imine-B($C_6F_5$)$_3$ adducts **6a-c**.[10]

## 6.3 Crystallographic data of Ir half sandwich complexes

**Table S1.** Selected crystallographic parameters for Ir-Cp* complexes, the ligand **3a**, the precursor **2a** and the adduct **3a-B($C_6F_5$)$_3$.**

| | **2a** | **3a** | **3a-B($C_6F_5$)$_3$** | **5a** | **5b** | **5d** |
|---|---|---|---|---|---|---|
| C1-N4 [Å] | 1.353(3) | 1.296(2) | 1.343(6) | 1.312(3) | 1.307(3) | 1.322(2) |
| N4-Ir1 [Å] | - | - | - | 2.068(2) | 2.055(2) | 2.096(2) |
| Cp-Ir1 [Å] | - | - | - | 1.820(1) | 1.820(1) | 1.808(1) |
| $C_{Phenyl}$-Ir1 [Å] | - | - | - | 2.053(2) | 2.062(3) | 2.062(3) |
| C1-N4-Ir1 [°] | - | - | - | 123.6(2) | 123.1(2) | 117.4(2) |

## 6.4 Preparation of **4**/**4'** solutions

The Ligand **3a** (11 mg, 0.04 mmol, 1 eq) was submitted into an oven-dried Schlenk-flask. Under a flow of argon, the ligand was dissolved in THF (7 mL). The solution was degassed by bubbling argon-gas through the solution for 10 minutes. [Ir(COD]Cl]$_2$ (16 mg, 0.02 mmol, 0.5 eq) was added as a solid and the solution was stirred over night at room temperature. The solvent was removed under reduced pressure and the residue was dissolved in $CD_2Cl_2$ (2 mL). The solution was transferred into three different NMR-tubes (0.5 mL respectively). Into the first two samples either a drop of $Et_3N$ or a drop ethereal HCl-solution were submitted. $^1$H-NMR-measurements were conducted immediately. Temperature-dependent $^1$H-NMR-measurments were conducted on an *Avance 400* from Bruker with the third sample.

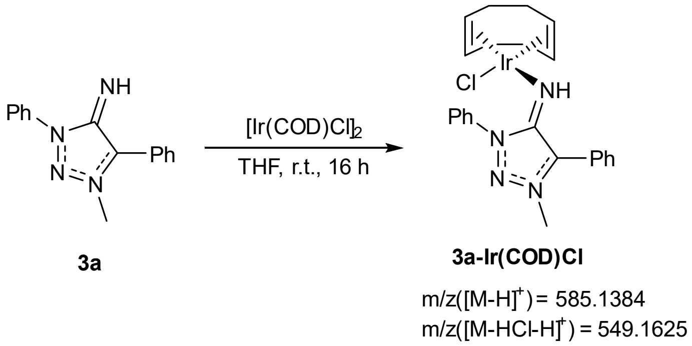


**Scheme S1.** Proposed reaction of **3a** with [Ir(COD)Cl]$_2$ according to found fragments by HRMS.

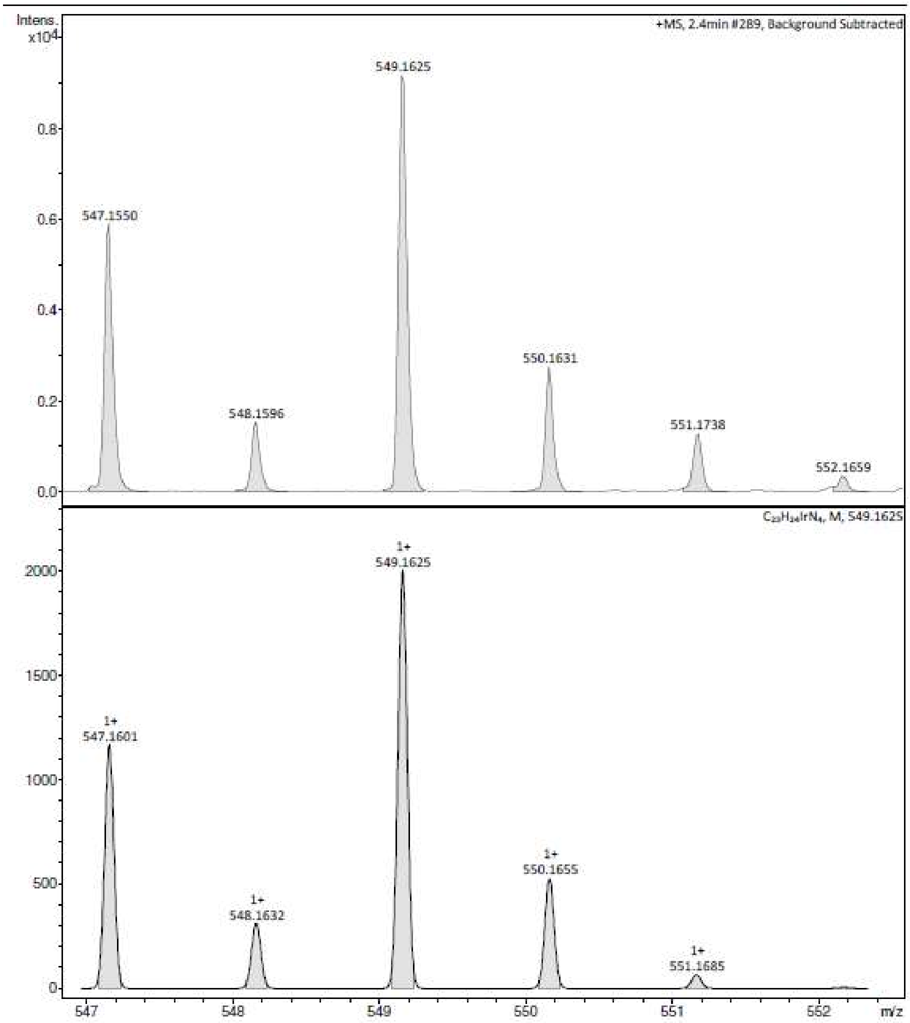


**Figure S51.** HRMS-spectrum of **3a-Ir(COD)Cl**.

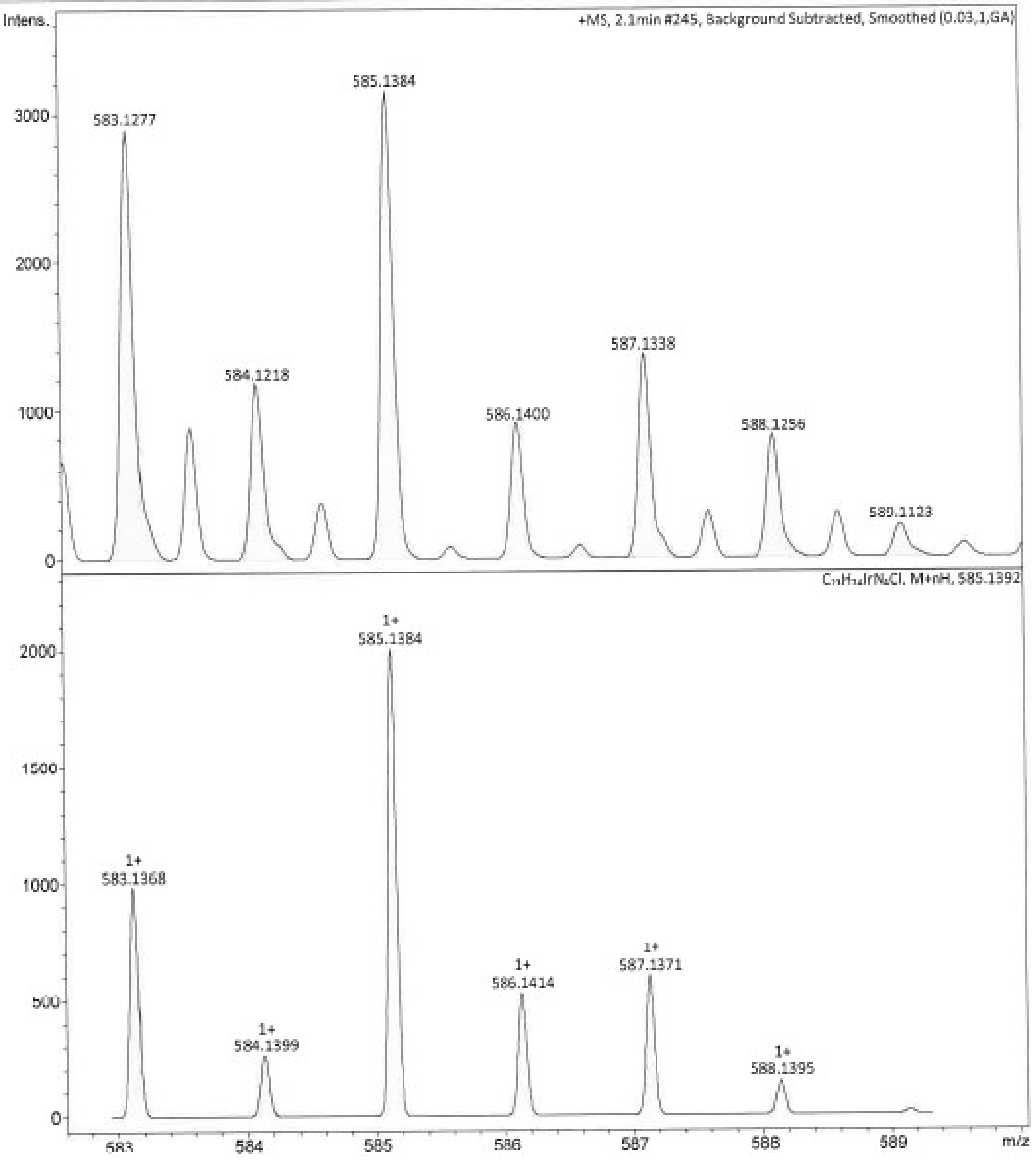


**Figure S52.** HRMS-spectrum of **3a-Ir(COD)Cl**.

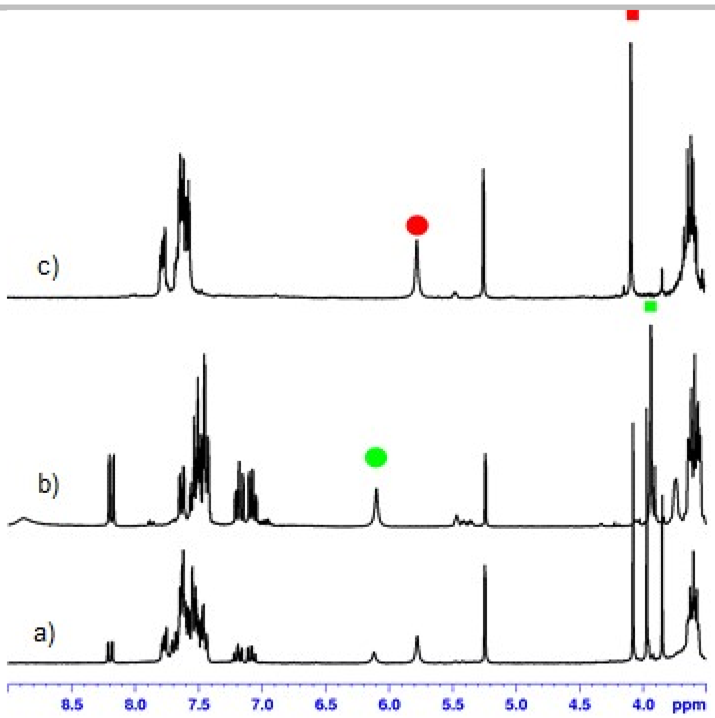


**Figure S53.** $^{1}$H-NMR-spectra of **3a** and $[Ir(COD)Cl]_2$ ($CD_2Cl_2$, 400 MHz). a) native. b) + $Et_3N$. c) + HCl in $Et_2O$.

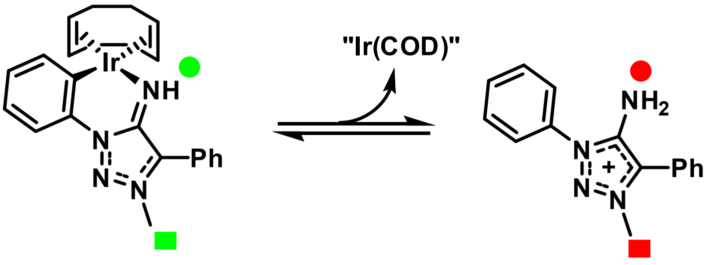


**Scheme S2.** Determined equilibrium between **4** and **4'**.

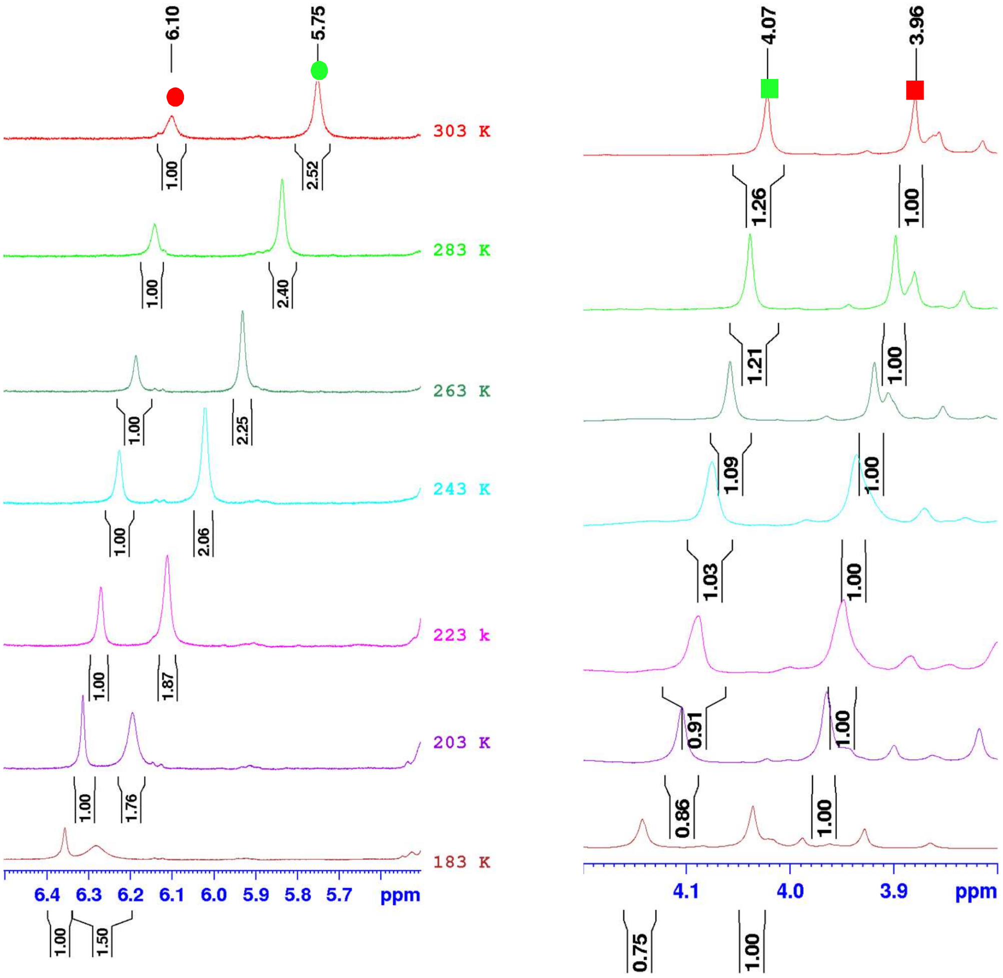

**Figure S54.** Selected parts of temperature-dependant $^1$H-NMR-measurements from the reaction mixture of **3a** with $[Ir(COD)Cl]_2$ in $CD_2Cl_2$ (400 MHz). Left: Region of the exocyclic protons. Right: Region of N-$CH_3$.

The equilibrium constant $K = \frac{[4']}{[4]}$ for the equilibrium according to scheme 2 can be calculated by the relative integrals of either the exocyclic protons ($K_{exo}$) and the $CH_3$-protons ($K_{Me}$). The values are summarised in the following table:

**Table S2.** Temperature-dependant equilibrium constants.

| $T$ $[K]$ | $\frac{1}{T}$ $[10^3 \cdot K^{-1}]$ | $K_{exo}$ | $ln\, K_{exo}$ | $K_{Me}$ | $ln\, K_{Me}$ |
|---|---|---|---|---|---|
| 303 | 3.30 | 1.26 | 0.23 | 1.26 | 0.23 |
| 283 | 3.53 | 1.20 | 0.18 | 1.21 | 0.19 |
| 263 | 3.80 | 1.13 | 0.12 | 1.09 | 0.09 |
| 243 | 4.12 | 1.03 | 0.03 | 1.03 | 0.03 |
| 223 | 4.48 | 0.94 | -0.06 | 0.91 | -0.09 |
| 203 | 4.93 | 0.88 | -0.13 | 0.86 | -0.15 |
| 183 | 5.46 | 0.75 | -0.29 | 0.75 | -0.29 |

According to *van't Hoff* the equilibrium constant and temperature can be set in a relation to

$$\ln K = -\frac{\Delta H}{R} \cdot \frac{1}{T} + \frac{\Delta S}{R}$$

with $\Delta H$: enthalpy and $\Delta S$: entropy.

Linear regression of the resulting *van’t Hoff* plots:

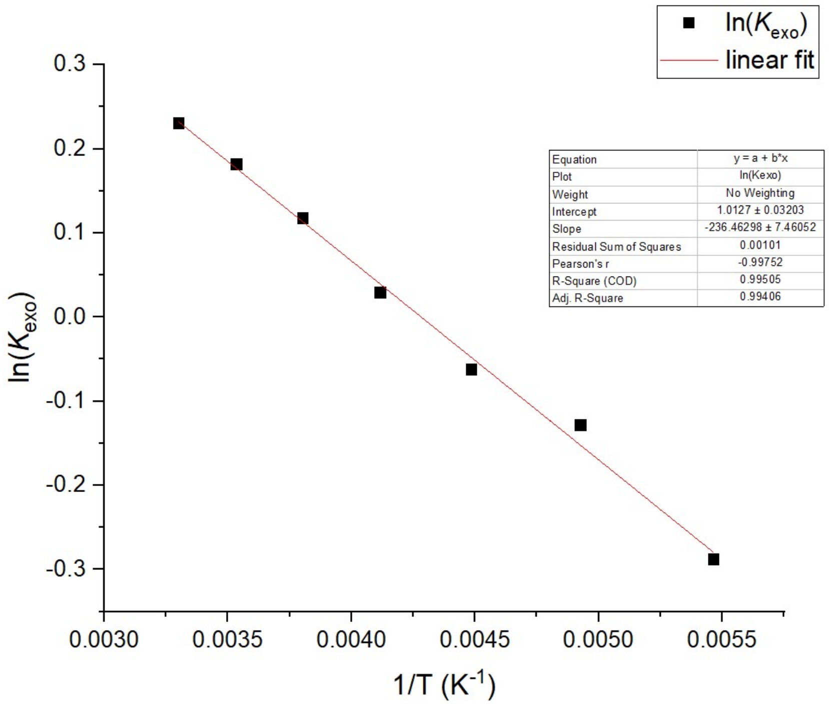


**Figure S55.** Van’t-Hoff plot of the equilibrium shown in scheme 2 according to the exocyclic protons

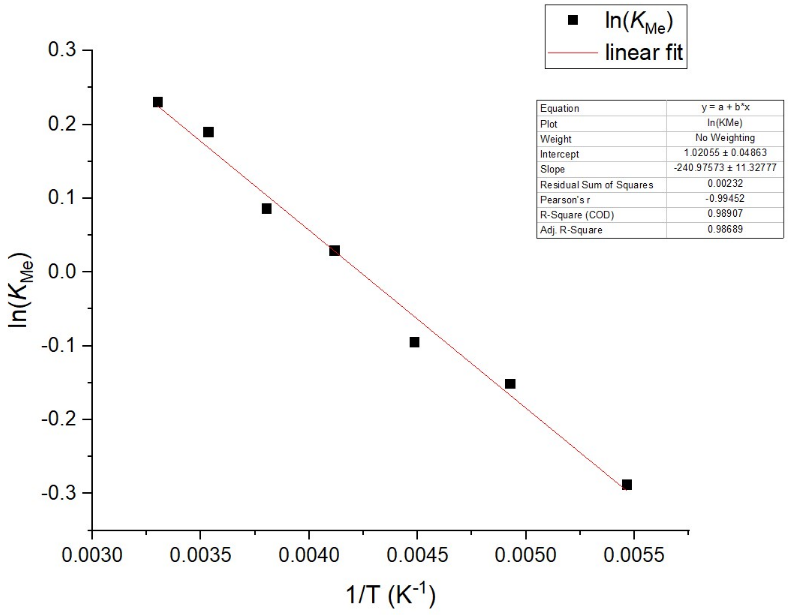


**Figure S56.** Van’t-Hoff plot of the equilibrium shown in scheme 2 according to the methyl protons

Examination of the *van't Hoff* plot leads to:

**Table S3**. Determined thermodynamic data for the equilibrium shown in scheme 2.

| | $\Delta H$ $\left[\frac{\mathrm{J}}{\mathrm{mol}}\right]$ | $\Delta S$ $\left[\frac{\mathrm{J}}{\mathrm{mol \cdot K}}\right]$ |
|---|---|---|
| **Fehler! Verweisquelle konnte nicht gefunden werden.** | $1966 \pm 0.90$ | $8.42 \pm 0.004$ |
| **Fehler! Verweisquelle konnte nicht gefunden werden.** | $2004 \pm 1.36$ | $8.48 \pm 0.006$ |

6.5 Preparation of **2a(Cl)**

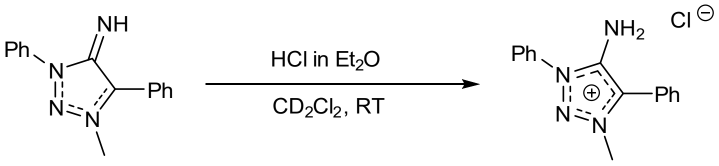


**3a** **2a(Cl)**

The triazolium chloride of **2a** was generated by the reaction of **3a** (5 mg, 0.02 mmol) with one drop of ethereal HCl-solution using $CD_2Cl_2$ (0.5 mL) as the solvent in an NMR-tube. $^1$H-NMR-measurements were conducted before and after addition of ethereal HCl.

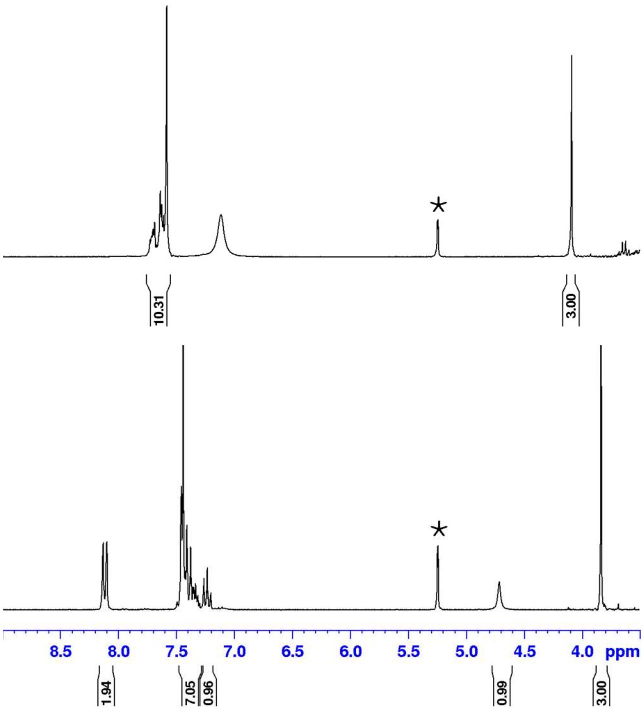


**Figure S57.** $^1$H-NMR-spectra ($CD_2Cl_2$ (*), 250 MHz). Bottom: **3a**. Top: Addition of ethereal HCl-solution.

7. Computational details

All calculations were performed using the ORCA 4.2.1 program.[11] Geometry optimizations were carried out using the PBE0 functional[12] with def2-SVP basis sets[13] on all atoms, starting from the X-ray determined structures, except for the **3a-CO₂** adduct. The optimized structures were used for single point and frequency calculations with the PBE0 functional and def2-TZVP basis sets. For complex **3a-B($C_6F_5$)$_3$**, the optimized structure was employed in single-point (but not frecuency) calculations, using the PBE0 functional and def2-TZVP basis sets, in order to calculate the electron density difference between the full complex and its **3a** and **B($C_6F_5$)$_3$** fragments, according to a methodology previously reported by our group.[14] Implicit solvation by $CH_2Cl_2$ was taken into account using the SMD method[14] together with the CPCM model.[15] The resolution-of-the-identity (RI) approximation[16] with matching basis sets (def2/J),[17] as well as the RIJCOSX approximation (combination of RI and chain-of-spheres algorithm for exchange integrals) were used to reduce the time of calculations. The optimized structures were confirmed to be minima by the absence of imaginary vibrational frequencies. Orbital and electron density isosurfaces were plot with Chemcraft[18].

### Frontier orbitals for the studied molecules

Figures S55 to S61 show the HOMO and LUMO orbitals of each of the calculated molecules.

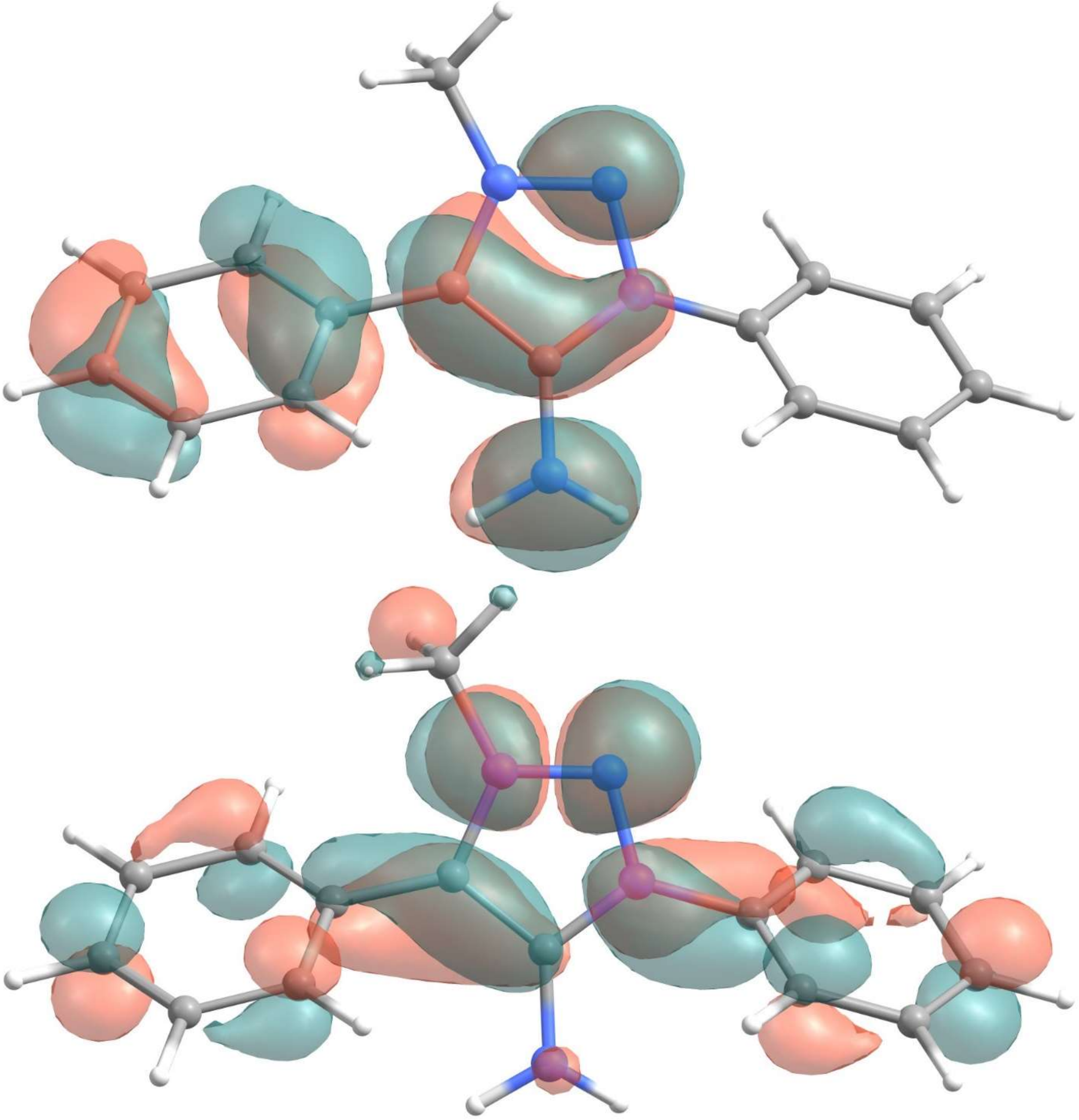

**Figure S58.** Isosurfaces corresponding to the HOMO (top) and LUMO (bottom) orbitals of the cation in compound **2a**. Isovalue = 0.03.

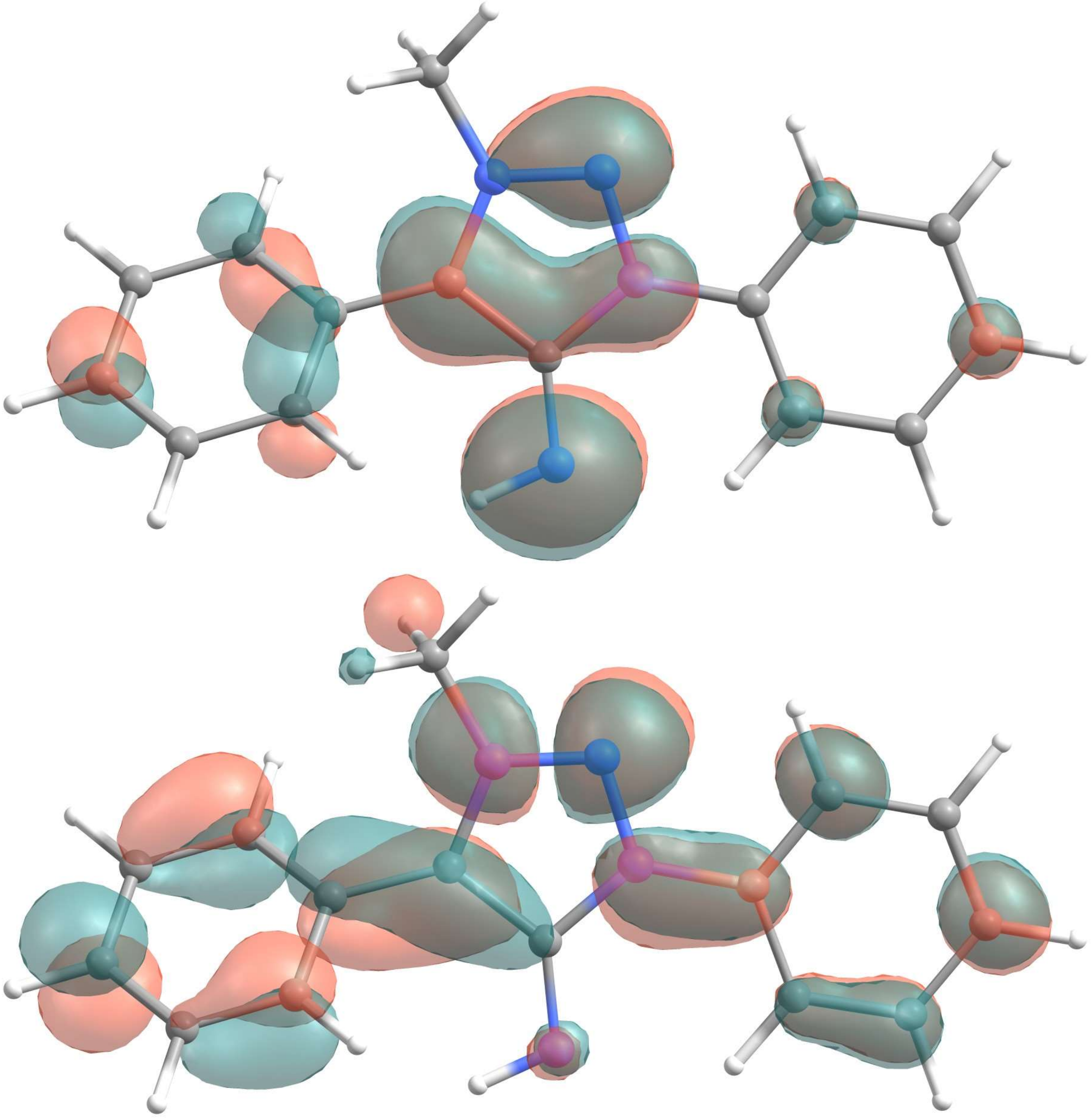

**Figure S59.** Isosurfaces corresponding to the HOMO (top) and LUMO (bottom) orbitals of **3a**. Isovalue = 0.03

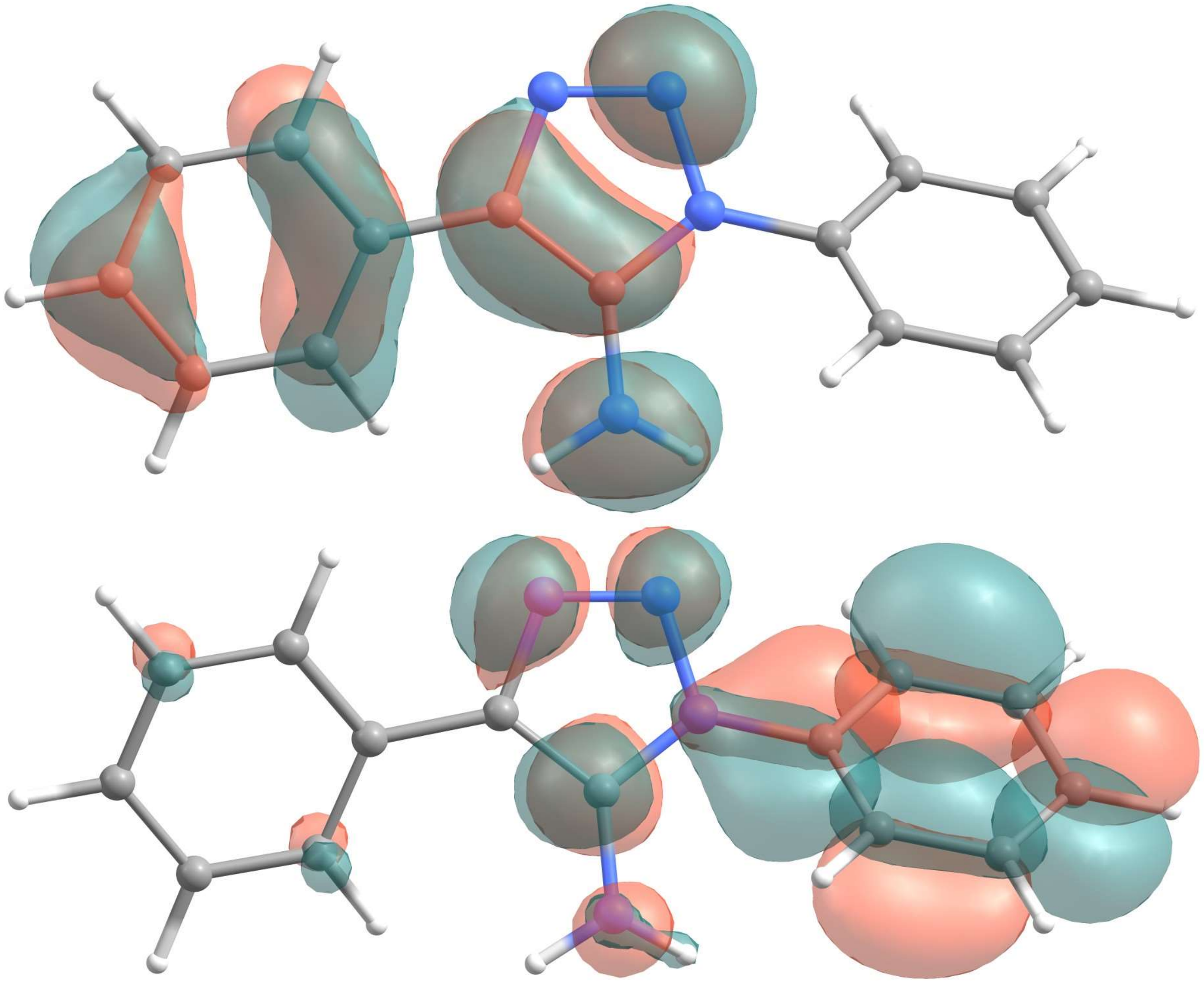

**Figure S60.** Isosurfaces corresponding to the HOMO (top) and LUMO (bottom) orbitals of **1a**. Isovalue = 0.03

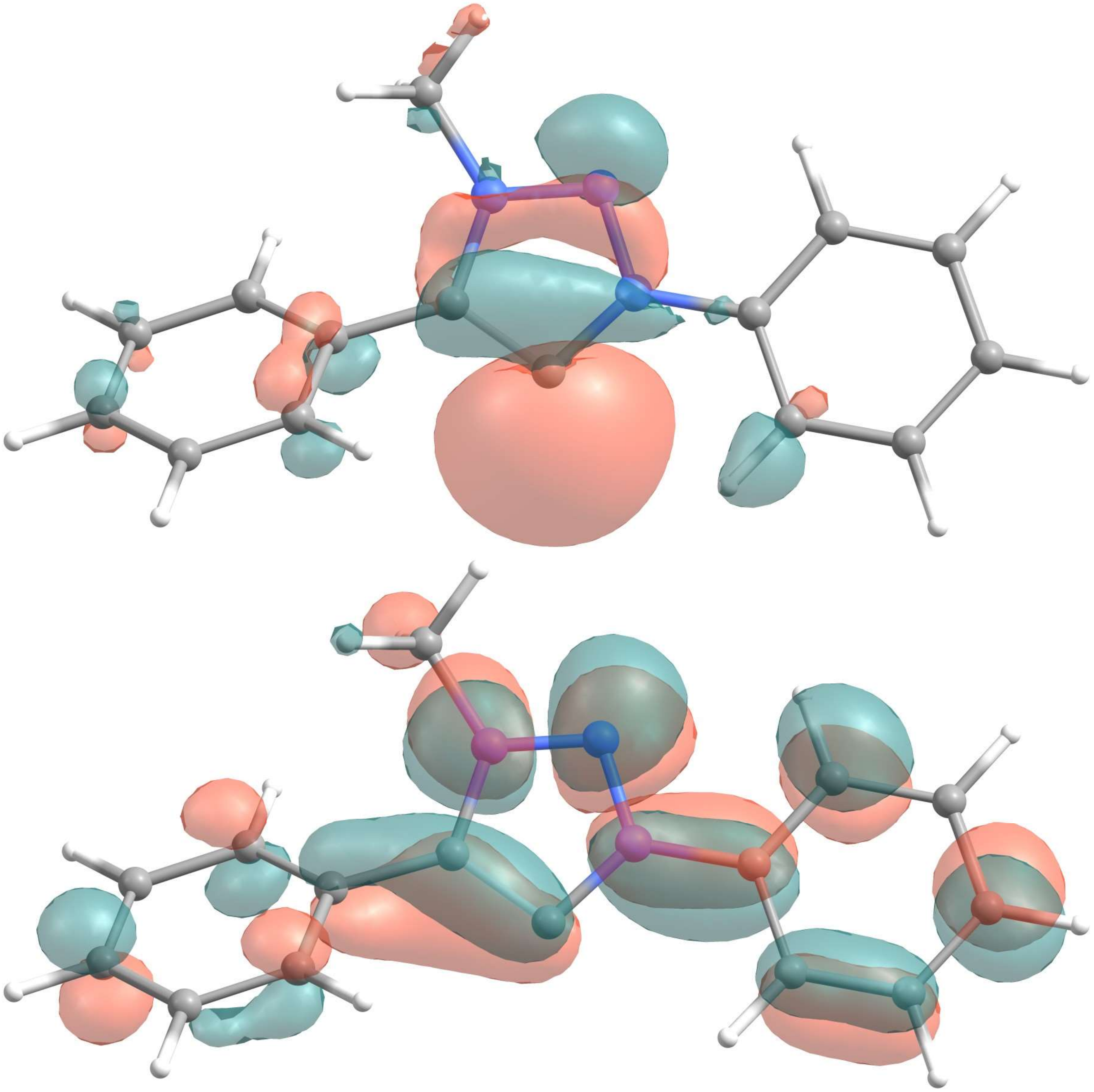

**Figure S61.** Isosurfaces corresponding to the HOMO (top) and LUMO (bottom) orbitals of **MIC-a**. Isovalue = 0.03.

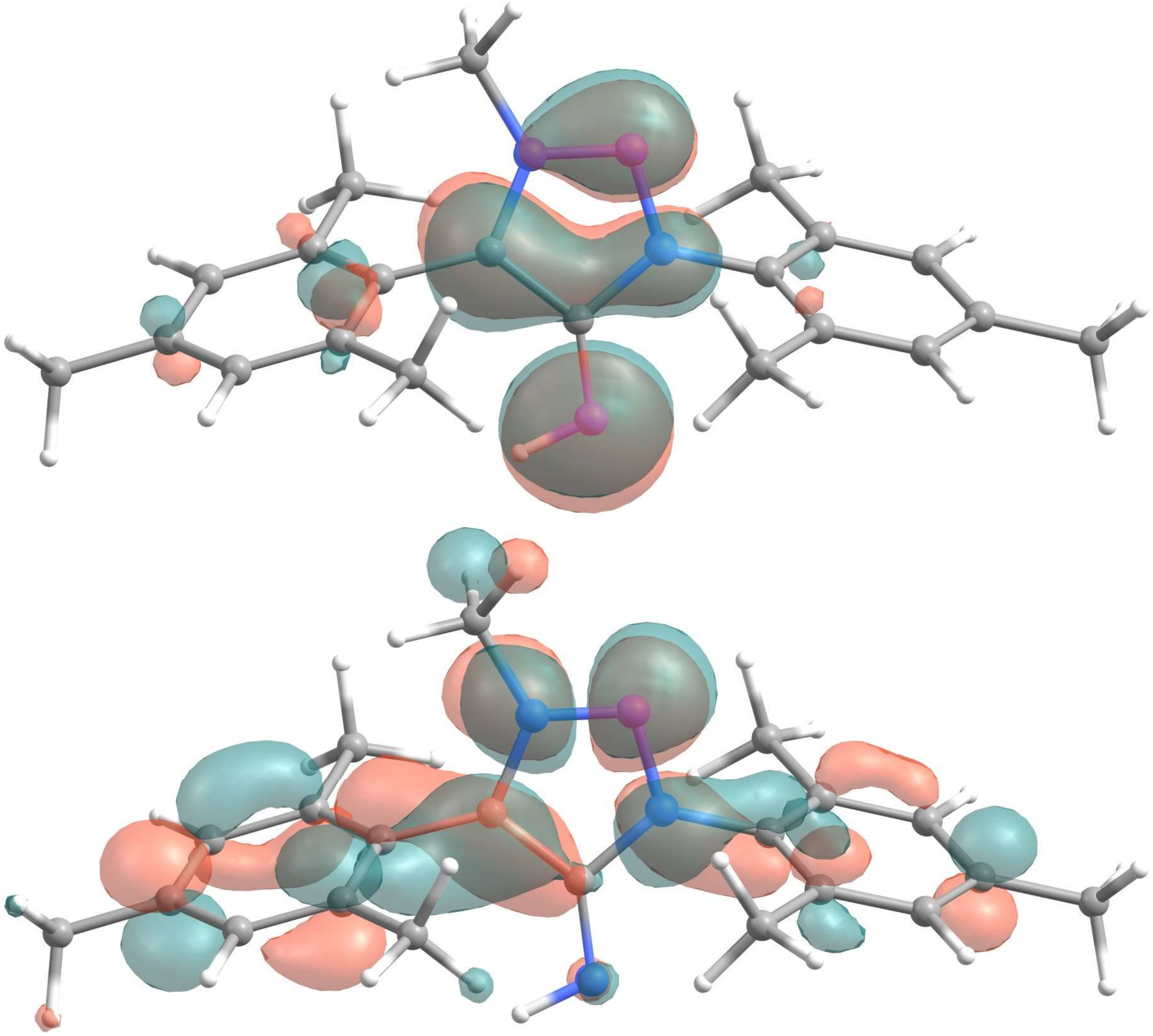

**Figure S62.** Isosurfaces corresponding to the HOMO (top) and LUMO (bottom) orbitals of **3c**. Isovalue = 0.03.

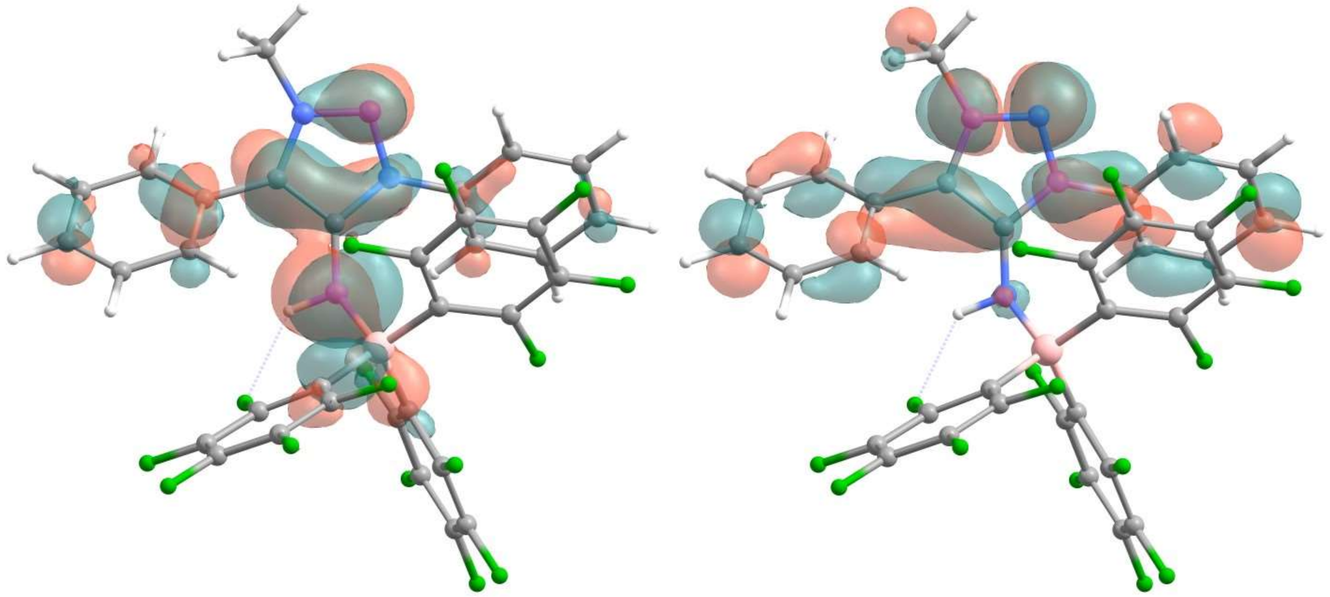

**Figure S63.** Isosurfaces corresponding to the HOMO (left) and LUMO (right) orbitals of **3a-B($C_6F_5$)$_3$**. Isovalue = 0.03.

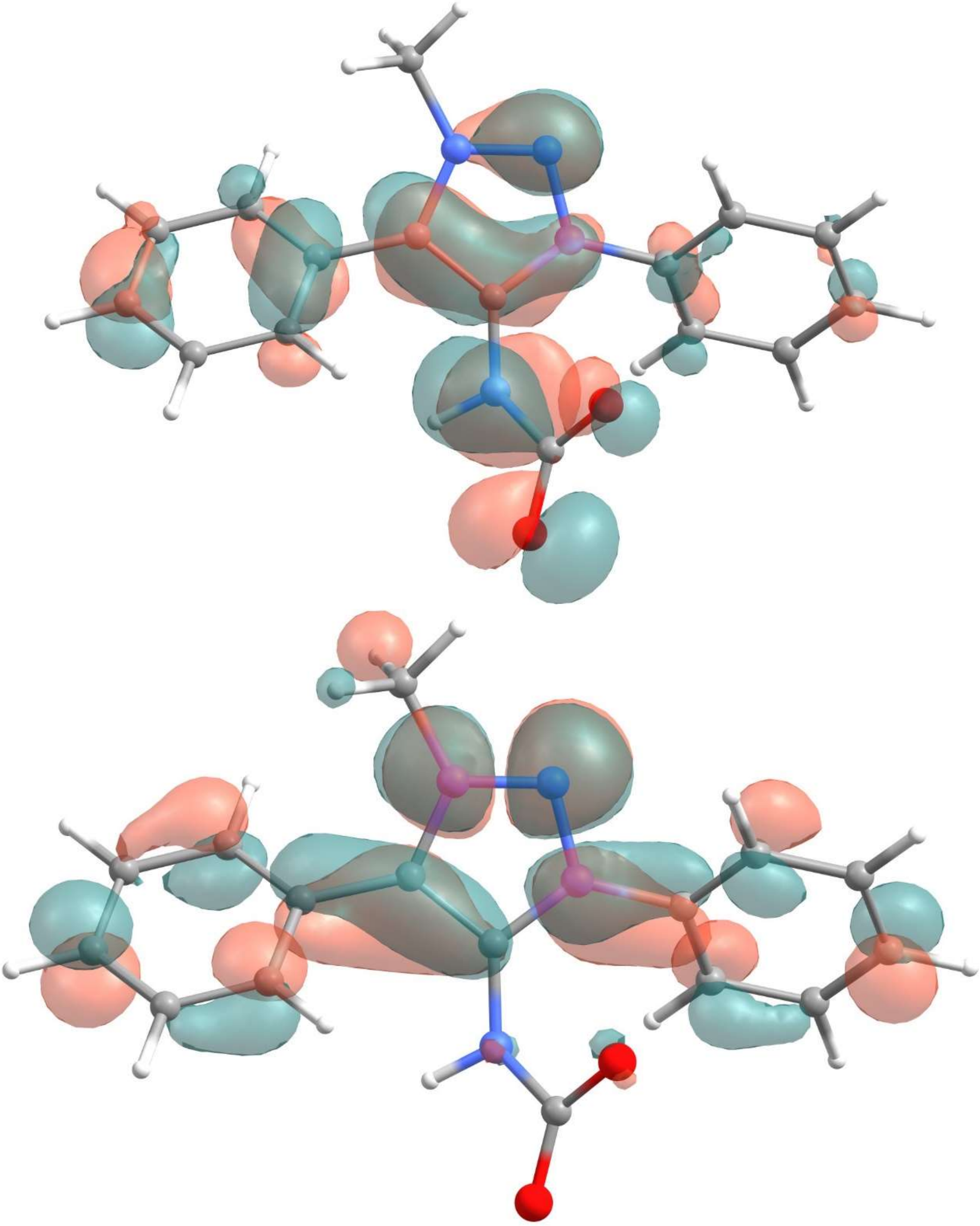

**Figure S64.** Isosurfaces corresponding to the HOMO (top) and LUMO (bottom) orbitals of **3a-CO₂**. Isovalue = 0.03.

**Table S4**. Single-point and Gibbs Free Energies (in Ha) and absolute HOMO and LUMO energies, as well as their difference $\Delta_{HL}$, in $cm^{-1}$, for selected compounds studied computationally.

| | PBE0/def2-TZVP/SMD($CH_2Cl_2$) | | | |
|---|---|---|---|---|
| | HOMO ($cm^{-1}$) | LUMO ($cm^{-1}$) | $\Delta_{HL}$ ($cm^{-1}$) | Gibbs (Ha) |
| (**2a**) | –57353 | –16750 | 40602 | -798.632 |
| (**3a**) | –42821 | –11458 | 31363 | -798.165 |
| (**1a**) | –48425 | –7238 | 41186 | -758.948 |
| (**MIC-a**) | –52296 | –11579 | 40717 | -742.829 |
| (**3a-B($C_6F_5$)$_3$**) | –51056 | –13634 | 37422 | -3001.746[a] |

| | | | | |
|---|---|---|---|---|
| (**3c**) | –41860 | –7113 | 34747 | -1033.719 |
| (**3a-CO₂**) | –52882 | –13427 | 39455 | -986.638 |

[a] Calculated at the PBE0/def2-SVP level.

**Optimized Geometries**

**2a – I⁻**

| | | | |
|---|---|---|---|
| N | -3.209478000000 | 2.880808000000 | -2.607647000000 |
| N | -3.208646000000 | 3.937176000000 | -1.859657000000 |
| C | -3.602668000000 | 1.749533000000 | -1.956927000000 |
| C | -3.871451000000 | 2.180166000000 | -0.653340000000 |
| N | -3.611861000000 | 3.518356000000 | -0.672256000000 |
| C | -3.677782000000 | 4.438761000000 | 0.414194000000 |
| C | -4.825487000000 | 4.486338000000 | 1.205093000000 |
| C | -2.587174000000 | 5.275232000000 | 0.649928000000 |
| C | -4.870056000000 | 5.389340000000 | 2.265340000000 |
| C | -2.653984000000 | 6.181524000000 | 1.704286000000 |
| C | -3.789704000000 | 6.235630000000 | 2.513810000000 |
| H | -5.677370000000 | 3.838580000000 | 0.989750000000 |
| H | -1.703016000000 | 5.209540000000 | 0.013371000000 |
| H | -5.762777000000 | 5.437164000000 | 2.892551000000 |
| H | -1.807605000000 | 6.843801000000 | 1.898787000000 |
| H | -3.833299000000 | 6.944080000000 | 3.344212000000 |
| C | -3.699497000000 | 0.404446000000 | -2.522567000000 |
| C | -2.689257000000 | -0.124541000000 | -3.341131000000 |
| C | -4.817710000000 | -0.386602000000 | -2.211714000000 |
| C | -2.806934000000 | -1.414974000000 | -3.849517000000 |
| C | -4.923546000000 | -1.679476000000 | -2.716990000000 |
| C | -3.921612000000 | -2.194728000000 | -3.539391000000 |
| H | -1.797361000000 | 0.462819000000 | -3.565996000000 |
| H | -5.617448000000 | 0.018092000000 | -1.586722000000 |
| H | -2.014799000000 | -1.817974000000 | -4.484721000000 |
| H | -5.799510000000 | -2.284588000000 | -2.472453000000 |
| H | -4.007855000000 | -3.208302000000 | -3.937725000000 |
| N | -4.319454000000 | 1.502520000000 | 0.418191000000 |
| H | -4.155524000000 | 1.904284000000 | 1.336725000000 |
| H | -4.228622000000 | 0.492166000000 | 0.382519000000 |
| C | -2.826701000000 | 3.017285000000 | -4.000004000000 |
| H | -1.752835000000 | 2.819846000000 | -4.113198000000 |
| H | -3.408387000000 | 2.309200000000 | -4.600429000000 |
| H | -3.050272000000 | 4.044580000000 | -4.306905000000 |

**3a**

| | | | |
|---|---|---|---|
| N | -2.909383000000 | 2.751155000000 | -2.443013000000 |
| N | -2.895989000000 | 3.805171000000 | -1.692850000000 |
| C | -3.549694000000 | 1.687117000000 | -1.906695000000 |
| C | -4.015514000000 | 2.124344000000 | -0.616124000000 |
| N | -3.550993000000 | 3.454278000000 | -0.578924000000 |
| C | -3.694421000000 | 4.421621000000 | 0.437829000000 |
| C | -4.399519000000 | 4.131618000000 | 1.613113000000 |
| C | -3.119588000000 | 5.689933000000 | 0.259929000000 |
| C | -4.522272000000 | 5.112445000000 | 2.596917000000 |
| C | -3.254701000000 | 6.653741000000 | 1.253057000000 |
| C | -3.955826000000 | 6.374010000000 | 2.427950000000 |
| H | -4.833685000000 | 3.138900000000 | 1.727426000000 |
| H | -2.570973000000 | 5.911807000000 | -0.654822000000 |
| H | -5.074167000000 | 4.877991000000 | 3.510834000000 |
| H | -2.803840000000 | 7.638041000000 | 1.103720000000 |
| H | -4.058149000000 | 7.135754000000 | 3.204520000000 |
| C | -3.735759000000 | 0.382783000000 | -2.528685000000 |
| C | -2.723907000000 | -0.255232000000 | -3.269803000000 |
| C | -4.963798000000 | -0.286347000000 | -2.361637000000 |
| C | -2.943349000000 | -1.506346000000 | -3.839933000000 |
| C | -5.172521000000 | -1.542273000000 | -2.924079000000 |
| C | -4.165809000000 | -2.157050000000 | -3.669454000000 |
| H | -1.746522000000 | 0.216614000000 | -3.381742000000 |
| H | -5.769667000000 | 0.195633000000 | -1.803717000000 |
| H | -2.143547000000 | -1.983786000000 | -4.411532000000 |

| | | | |
|---|---|---|---|
| H | -6.135516000000 | -2.040384000000 | -2.786604000000 |
| H | -4.331531000000 | -3.141947000000 | -4.112369000000 |
| N | -4.673483000000 | 1.548349000000 | 0.337732000000 |
| H | -4.858158000000 | 0.580584000000 | 0.077579000000 |
| C | -2.278221000000 | 2.850571000000 | -3.742766000000 |
| H | -1.250339000000 | 2.466882000000 | -3.697558000000 |
| H | -2.859330000000 | 2.276922000000 | -4.474204000000 |
| H | -2.263072000000 | 3.909659000000 | -4.022377000000 |

**1a**

| | | | |
|---|---|---|---|
| N | -3.923095000000 | 2.841426000000 | -2.563545000000 |
| N | -3.899632000000 | 3.886725000000 | -1.826215000000 |
| C | -3.764473000000 | 1.715352000000 | -1.814207000000 |
| C | -3.648965000000 | 2.121743000000 | -0.478839000000 |
| N | -3.743287000000 | 3.478011000000 | -0.542305000000 |
| C | -3.670986000000 | 4.432655000000 | 0.499800000000 |
| C | -4.345829000000 | 4.221002000000 | 1.704998000000 |
| C | -2.934434000000 | 5.601498000000 | 0.289532000000 |
| C | -4.263434000000 | 5.183281000000 | 2.710234000000 |
| C | -2.875188000000 | 6.561840000000 | 1.295518000000 |
| C | -3.532409000000 | 6.353612000000 | 2.509292000000 |
| H | -4.939184000000 | 3.317436000000 | 1.853777000000 |
| H | -2.418812000000 | 5.745728000000 | -0.661072000000 |
| H | -4.789455000000 | 5.018612000000 | 3.653453000000 |
| H | -2.302410000000 | 7.477504000000 | 1.130881000000 |
| H | -3.476457000000 | 7.106724000000 | 3.298849000000 |
| C | -3.755322000000 | 0.381681000000 | -2.415861000000 |
| C | -3.154378000000 | -0.722983000000 | -1.789918000000 |
| C | -4.349622000000 | 0.190651000000 | -3.675928000000 |
| C | -3.167307000000 | -1.979413000000 | -2.393058000000 |
| C | -4.354012000000 | -1.063326000000 | -4.278531000000 |
| C | -3.769389000000 | -2.157970000000 | -3.637933000000 |
| H | -2.634205000000 | -0.605832000000 | -0.836909000000 |
| H | -4.811076000000 | 1.045185000000 | -4.175018000000 |
| H | -2.691505000000 | -2.823251000000 | -1.886999000000 |
| H | -4.823975000000 | -1.189422000000 | -5.257265000000 |
| H | -3.778886000000 | -3.143592000000 | -4.109784000000 |
| N | -3.551998000000 | 1.433795000000 | 0.688667000000 |
| H | -3.076357000000 | 1.888678000000 | 1.461787000000 |
| H | -3.343996000000 | 0.447041000000 | 0.613544000000 |

**MIC-a**

| | | | |
|---|---|---|---|
| N | -2.928387000000 | 2.795120000000 | -2.503191000000 |
| N | -2.915703000000 | 3.842249000000 | -1.735631000000 |
| C | -3.614348000000 | 1.753964000000 | -1.930138000000 |
| C | -4.090845000000 | 2.183590000000 | -0.685937000000 |
| N | -3.601711000000 | 3.462688000000 | -0.670436000000 |
| C | -3.770185000000 | 4.412080000000 | 0.381019000000 |
| C | -4.559666000000 | 4.069643000000 | 1.479320000000 |
| C | -3.154199000000 | 5.663722000000 | 0.308946000000 |
| C | -4.732653000000 | 4.989145000000 | 2.510710000000 |
| C | -3.336767000000 | 6.574081000000 | 1.347465000000 |
| C | -4.124238000000 | 6.243183000000 | 2.450458000000 |
| H | -5.025350000000 | 3.084162000000 | 1.505700000000 |
| H | -2.537600000000 | 5.919324000000 | -0.552820000000 |
| H | -5.351790000000 | 4.719625000000 | 3.369768000000 |
| H | -2.855041000000 | 7.553065000000 | 1.290216000000 |
| H | -4.263229000000 | 6.961479000000 | 3.261915000000 |
| C | -3.804677000000 | 0.441615000000 | -2.559892000000 |
| C | -2.812791000000 | -0.192284000000 | -3.325096000000 |
| C | -5.021191000000 | -0.228358000000 | -2.347571000000 |
| C | -3.042530000000 | -1.450631000000 | -3.879154000000 |
| C | -5.245184000000 | -1.486260000000 | -2.898533000000 |
| C | -4.258445000000 | -2.100649000000 | -3.671922000000 |
| H | -1.839858000000 | 0.279670000000 | -3.470004000000 |
| H | -5.790592000000 | 0.256698000000 | -1.743055000000 |
| H | -2.257313000000 | -1.930133000000 | -4.468859000000 |
| H | -6.199610000000 | -1.989967000000 | -2.726179000000 |
| H | -4.435089000000 | -3.087389000000 | -4.106797000000 |
| C | -2.284014000000 | 2.890128000000 | -3.796657000000 |
| H | -2.854831000000 | 2.307576000000 | -4.529105000000 |

| | | | |
|---|---|---|---|
| H | -2.270034000000 | 3.945293000000 | -4.091455000000 |
| H | -1.254044000000 | 2.512291000000 | -3.741461000000 |

**3c**

| | | | |
|---|---|---|---|
| N | -3.882438000000 | 2.945859000000 | -2.652887000000 |
| N | -3.894864000000 | 4.000326000000 | -1.900681000000 |
| C | -3.809771000000 | 1.771872000000 | -1.977392000000 |
| C | -3.757282000000 | 2.125722000000 | -0.589047000000 |
| N | -3.816506000000 | 3.522520000000 | -0.653852000000 |
| C | -3.746405000000 | 4.430081000000 | 0.435517000000 |
| C | -4.818042000000 | 4.491284000000 | 1.342376000000 |
| C | -2.608146000000 | 5.239387000000 | 0.571826000000 |
| C | -4.721261000000 | 5.387670000000 | 2.408041000000 |
| C | -2.564155000000 | 6.128426000000 | 1.650784000000 |
| C | -3.605613000000 | 6.216072000000 | 2.578709000000 |
| H | -5.547807000000 | 5.445401000000 | 3.122675000000 |
| H | -1.682045000000 | 6.763630000000 | 1.772505000000 |
| C | -3.784717000000 | 0.432734000000 | -2.576785000000 |
| C | -2.684980000000 | -0.005633000000 | -3.344286000000 |
| C | -4.867902000000 | -0.445959000000 | -2.327141000000 |
| C | -2.697313000000 | -1.305340000000 | -3.864936000000 |
| C | -4.830936000000 | -1.734826000000 | -2.857136000000 |
| C | -3.756012000000 | -2.184568000000 | -3.634765000000 |
| H | -1.840895000000 | -1.643678000000 | -4.456007000000 |
| H | -5.670898000000 | -2.409163000000 | -2.663789000000 |
| N | -3.680556000000 | 1.467566000000 | 0.527251000000 |
| H | -3.609953000000 | 0.472056000000 | 0.314142000000 |
| C | -3.964636000000 | 3.123502000000 | -4.086589000000 |
| H | -2.983921000000 | 3.413440000000 | -4.486223000000 |
| H | -4.289855000000 | 2.181716000000 | -4.542017000000 |
| H | -4.695330000000 | 3.912620000000 | -4.299114000000 |
| C | -6.018251000000 | 3.615752000000 | 1.164586000000 |
| H | -6.429954000000 | 3.701321000000 | 0.146519000000 |
| H | -5.728275000000 | 2.560972000000 | 1.305799000000 |
| H | -6.808796000000 | 3.873691000000 | 1.882288000000 |
| C | -3.539401000000 | 7.171863000000 | 3.733280000000 |
| H | -4.374130000000 | 7.890689000000 | 3.702114000000 |
| H | -3.613759000000 | 6.639350000000 | 4.695162000000 |
| H | -2.600371000000 | 7.743099000000 | 3.733010000000 |
| C | -1.471275000000 | 5.153108000000 | -0.401671000000 |
| H | -1.151623000000 | 4.110285000000 | -0.555942000000 |
| H | -1.759019000000 | 5.544296000000 | -1.390002000000 |
| H | -0.605980000000 | 5.727765000000 | -0.044322000000 |
| C | -6.045702000000 | 0.006147000000 | -1.516124000000 |
| H | -6.817214000000 | -0.774574000000 | -1.466788000000 |
| H | -5.744797000000 | 0.260627000000 | -0.486759000000 |
| H | -6.501133000000 | 0.914291000000 | -1.942479000000 |
| C | -1.501821000000 | 0.879539000000 | -3.607656000000 |
| H | -1.359116000000 | 1.624071000000 | -2.810785000000 |
| H | -0.580447000000 | 0.285323000000 | -3.692153000000 |
| H | -1.611892000000 | 1.429630000000 | -4.556992000000 |
| C | -3.750499000000 | -3.577005000000 | -4.192319000000 |
| H | -2.839239000000 | -3.777448000000 | -4.773374000000 |
| H | -3.813483000000 | -4.327501000000 | -3.387800000000 |
| H | -4.617744000000 | -3.744449000000 | -4.851475000000 |

**3a-B($C_6F_5$)$_3$**

| | | | |
|---|---|---|---|
| F | 11.073570000000 | 4.168614000000 | 10.263553000000 |
| F | 7.086866000000 | 5.070043000000 | 11.862261000000 |
| F | 8.305268000000 | 0.779643000000 | 8.449576000000 |
| F | 9.813045000000 | 5.289750000000 | 7.997728000000 |
| F | 9.193618000000 | 2.863628000000 | 6.613640000000 |
| F | 10.208090000000 | 7.893740000000 | 8.445363000000 |
| F | 9.051403000000 | 9.100727000000 | 10.593680000000 |
| F | 7.489815000000 | 7.642065000000 | 12.274377000000 |
| F | 5.475790000000 | 4.399016000000 | 9.120990000000 |
| F | 7.763968000000 | 3.050970000000 | 4.413092000000 |
| F | 10.411868000000 | -0.832150000000 | 8.300750000000 |
| F | 13.140427000000 | 2.554032000000 | 10.085819000000 |
| F | 12.865488000000 | 0.032627000000 | 9.113050000000 |
| F | 4.057575000000 | 4.549899000000 | 6.886575000000 |

| | | | |
|---|---|---|---|
| F | 5.175393000000 | 3.885141000000 | 4.492246000000 |
| C | 8.856326000000 | 7.809619000000 | 10.370323000000 |
| C | 7.872667000000 | 5.699496000000 | 10.980618000000 |
| C | 8.447799000000 | 5.031858000000 | 9.903148000000 |
| C | 9.480361000000 | 1.263237000000 | 8.873625000000 |
| C | 8.065514000000 | 7.059422000000 | 11.228933000000 |
| C | 6.088771000000 | 4.036812000000 | 7.981269000000 |
| C | 9.220434000000 | 5.835178000000 | 9.063538000000 |
| C | 7.404376000000 | 3.574250000000 | 8.012577000000 |
| C | 7.934273000000 | 3.273907000000 | 6.758149000000 |
| C | 9.445936000000 | 7.189253000000 | 9.273157000000 |
| C | 9.561018000000 | 2.566518000000 | 9.364123000000 |
| C | 10.562881000000 | 0.397777000000 | 8.775490000000 |
| C | 10.840248000000 | 2.962522000000 | 9.750739000000 |
| C | 5.317778000000 | 4.139228000000 | 6.828995000000 |
| C | 7.204231000000 | 3.367070000000 | 5.573740000000 |
| C | 5.884877000000 | 3.799650000000 | 5.606863000000 |
| C | 11.818410000000 | 0.840488000000 | 9.176399000000 |
| C | 11.955364000000 | 2.131708000000 | 9.665629000000 |
| B | 8.195699000000 | 3.463469000000 | 9.476996000000 |
| N | 6.729512000000 | -0.503084000000 | 11.674516000000 |
| N | 8.210158000000 | 0.957071000000 | 11.780213000000 |
| N | 7.215576000000 | 2.749157000000 | 10.466805000000 |
| H | 6.257016000000 | 3.057940000000 | 10.341881000000 |
| N | 7.909890000000 | -0.274957000000 | 12.160490000000 |
| C | 6.231915000000 | 0.533019000000 | 10.954746000000 |
| C | 7.225370000000 | 1.525097000000 | 11.003955000000 |
| C | 9.420358000000 | 1.529010000000 | 12.268298000000 |
| C | 4.952446000000 | 0.571032000000 | 10.245076000000 |
| C | 4.930324000000 | 1.005448000000 | 8.909571000000 |
| H | 5.867118000000 | 1.256940000000 | 8.409899000000 |
| C | 3.748839000000 | 0.222275000000 | 10.875454000000 |
| H | 3.747313000000 | -0.091350000000 | 11.921342000000 |
| C | 3.723320000000 | 1.095352000000 | 8.222718000000 |
| H | 3.713981000000 | 1.434907000000 | 7.184536000000 |
| C | 2.546360000000 | 0.301884000000 | 10.177288000000 |
| H | 1.613668000000 | 0.028965000000 | 10.676291000000 |
| C | 2.530026000000 | 0.742808000000 | 8.853879000000 |
| H | 1.583656000000 | 0.812005000000 | 8.312244000000 |
| C | 9.426088000000 | 2.851845000000 | 12.708332000000 |
| H | 8.517496000000 | 3.451027000000 | 12.658846000000 |
| C | 10.615521000000 | 3.391249000000 | 13.188848000000 |
| H | 10.635594000000 | 4.430909000000 | 13.522249000000 |
| C | 10.562683000000 | 0.732793000000 | 12.327845000000 |
| H | 10.525218000000 | -0.302164000000 | 11.984691000000 |
| C | 6.111683000000 | -1.790322000000 | 11.921409000000 |
| H | 6.911779000000 | -2.509276000000 | 12.127644000000 |
| H | 5.550630000000 | -2.094926000000 | 11.030540000000 |
| H | 5.439476000000 | -1.723255000000 | 12.786797000000 |
| C | 11.774184000000 | 2.615884000000 | 13.238293000000 |
| H | 12.706769000000 | 3.049936000000 | 13.605412000000 |
| C | 11.742720000000 | 1.286271000000 | 12.818076000000 |
| H | 12.647845000000 | 0.675959000000 | 12.854749000000 |

**3a-$CO_2$**

| | | | |
|---|---|---|---|
| N | -3.724742000000 | 2.936328000000 | -2.598506000000 |
| N | -3.795793000000 | 3.987345000000 | -1.841123000000 |
| C | -3.635950000000 | 1.773302000000 | -1.897524000000 |
| C | -3.655191000000 | 2.167024000000 | -0.555137000000 |
| N | -3.734805000000 | 3.534604000000 | -0.605368000000 |
| C | -3.713917000000 | 4.481434000000 | 0.461438000000 |
| C | -4.553142000000 | 5.589617000000 | 0.389870000000 |
| C | -2.823086000000 | 4.305763000000 | 1.517669000000 |
| C | -4.497934000000 | 6.543009000000 | 1.403568000000 |
| C | -2.782982000000 | 5.263657000000 | 2.526412000000 |
| C | -3.617334000000 | 6.381281000000 | 2.473135000000 |
| H | -5.244693000000 | 5.694924000000 | -0.447531000000 |
| H | -2.178864000000 | 3.426729000000 | 1.554891000000 |
| H | -5.154657000000 | 7.414833000000 | 1.358395000000 |
| H | -2.089755000000 | 5.134701000000 | 3.360839000000 |
| H | -3.581335000000 | 7.128710000000 | 3.269280000000 |

| | | | |
|---|---|---|---|
| C | -3.585912000000 | 0.422428000000 | -2.458325000000 |
| C | -2.762584000000 | 0.105968000000 | -3.549668000000 |
| C | -4.367568000000 | -0.587187000000 | -1.872390000000 |
| C | -2.736989000000 | -1.191811000000 | -4.054162000000 |
| C | -4.331487000000 | -1.883224000000 | -2.378117000000 |
| C | -3.519981000000 | -2.188064000000 | -3.471464000000 |
| H | -2.121604000000 | 0.868448000000 | -3.995459000000 |
| H | -5.017189000000 | -0.353841000000 | -1.025978000000 |
| H | -2.091700000000 | -1.426751000000 | -4.903851000000 |
| H | -4.948256000000 | -2.658487000000 | -1.917751000000 |
| H | -3.495434000000 | -3.205860000000 | -3.868109000000 |
| N | -3.595372000000 | 1.435231000000 | 0.573443000000 |
| H | -3.199546000000 | 0.506954000000 | 0.464548000000 |
| C | -3.771711000000 | 3.114479000000 | -4.035825000000 |
| H | -2.754129000000 | 3.123528000000 | -4.447316000000 |
| H | -4.352132000000 | 2.298277000000 | -4.480706000000 |
| H | -4.259801000000 | 4.074550000000 | -4.234308000000 |
| C | -4.634280000000 | 1.523782000000 | 1.648864000000 |
| O | -5.399611000000 | 2.482920000000 | 1.530913000000 |
| O | -4.546282000000 | 0.607396000000 | 2.463624000000 |

8. Crystallographic data

Crystal data and structure refinement for **1a**

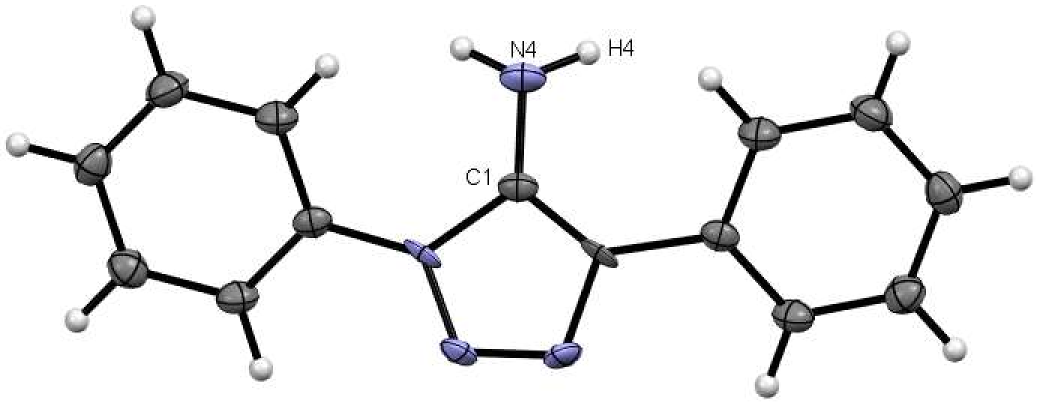


**Figure S65.** *X-ray solid-state structure of **1a**. Ellipsoids are all set at 50 % probability. Selected bond parameters in [Å] and [°]. C1-N4 1.362(2).*

| | | |
|---|---|---|
| Empirical formula | $C_{14}H_{12}N_4$ | |
| Formula weight | 236.28 | |
| CCDC Deposit Number | 2082293 | |
| Temperature | 140.0 K | |
| Wavelength | 0.71073 Å | |
| Crystal system | Orthorhombic | |
| Space group | Pnma | |
| Unit cell dimensions | a = 6.3776(5) Å | α = 90°. |
| | b = 25.2845(19) Å | β = 90°. |
| | c = 7.1474(5) Å | γ = 90°. |
| Volume | 1152.55(15) $Å^3$ | |
| Z | 4 | |
| Density (calculated) | 1.362 $g/m^3$ | |
| Absorption coefficient | 0.086 $mm^{-1}$ | |

| | |
|---|---|
| F(000) | 496 |
| Crystal size | 0.364 x 0.31 x 0.18 $mm^3$ |
| Theta range for data collection | 1.611 to 28.338°. |
| Index ranges | -8 ≤ h ≤ 8, -33 ≤ k ≤ 19, -9 ≤ l ≤ 9 |
| Reflections collected | 10625 |
| Independent reflections | 1475 [R(int) = 0.0273] |
| Completeness to theta = 25.242° | 99.9 % |
| Refinement method | Full-matrix least-squares on $F^2$ |
| Data / restraints / parameters | 1475 / 52 / 96 |
| Goodness-of-fit on $F^2$ | 1.087 |
| Final R indices [I>2σ(I)] | $R_1$ = 0.0385, $wR_2$ = 0.0987 |
| R indices (all data) | $R_1$ = 0.0457, $wR_2$ = 0.1039 |
| Extinction coefficient | 0.012(2) |
| Largest diff. peak and hole | 0.310 and -0.166 e.$Å^{-3}$ |

Crystal data and structure refinement for **1c**

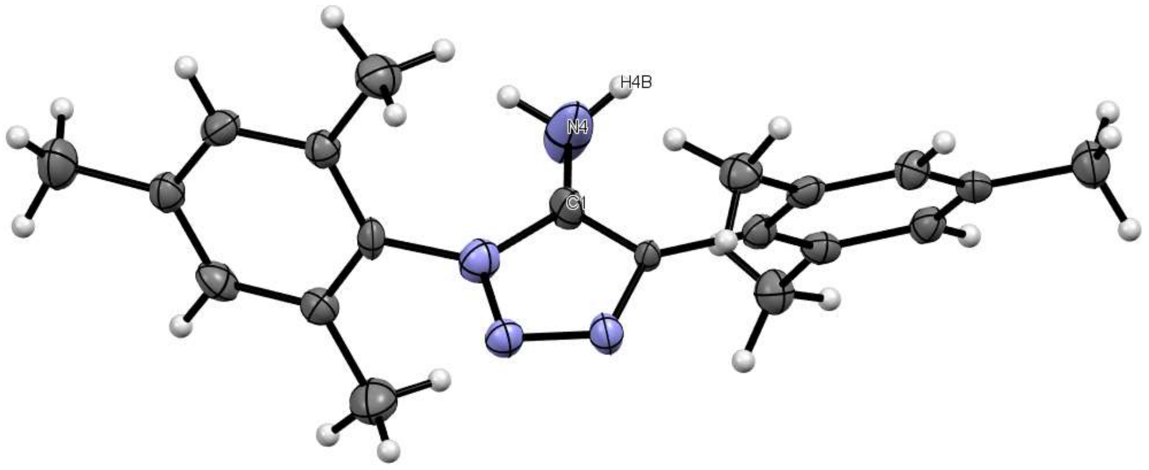


**Figure S66.** *X-ray solid-state structure of* ***1c****. Ellipsoids are all set at 50 % probability. Selected bond parameters in [Å] and [°]. C1-N4 1.359(4).*

| | | |
|---|---|---|
| Empirical formula | $C_{20}H_4N_4$ | |
| Formula weight | 320.43 | |
| CCDC Deposit Number | 2120117 | |
| Temperature | 139.99 K | |
| Wavelength | 0.71073 Å | |
| Crystal system | Monoclinic | |
| Space group | P21/c | |
| Unit cell dimensions | a = 16.110(2) Å | α = 90°. |
| | b = 7.5688(9) Å | β = 116.821(9)°. |
| | c = 17.043(2) Å | γ = 90°. |
| Volume | 1854.5(5) $Å^3$ | |
| Z | 4 | |
| Density (calculated) | 1.148 $g/m^3$ | |

| | |
|---|---|
| Absorption coefficient | 0.070 $mm^{-1}$ |
| F(000) | 688.0 |
| Crystal size | 0.14 × 0.13 × 0.051 $mm^3$ |
| Theta range for data collection | 2.832 to 50.744°. |
| Index ranges | -19 ≤ h ≤ 19, -8 ≤ k ≤ 8, -20 ≤ l ≤ 20 |
| Reflections collected | 18697 |
| Independent reflections | 3401 [$R_{int}$ = 0.1037, $R_{sigma}$ = 0.1057] |
| Data / restraints / parameters | 3401 / 144 / 224 |
| Goodness-of-fit on $F^2$ | 1.011 |
| Final R indices [I≥2σ(I)] | $R_1$ = 0.0649, $wR_2$ = 0.1364 |
| R indices (all data) | $R_1$ = 0.1507, $wR_2$ = 0.1680 |
| Largest diff. peak and hole | 0.38 and -0.36 e.$Å^{-3}$ |

Crystal data and structure refinement for **1d**

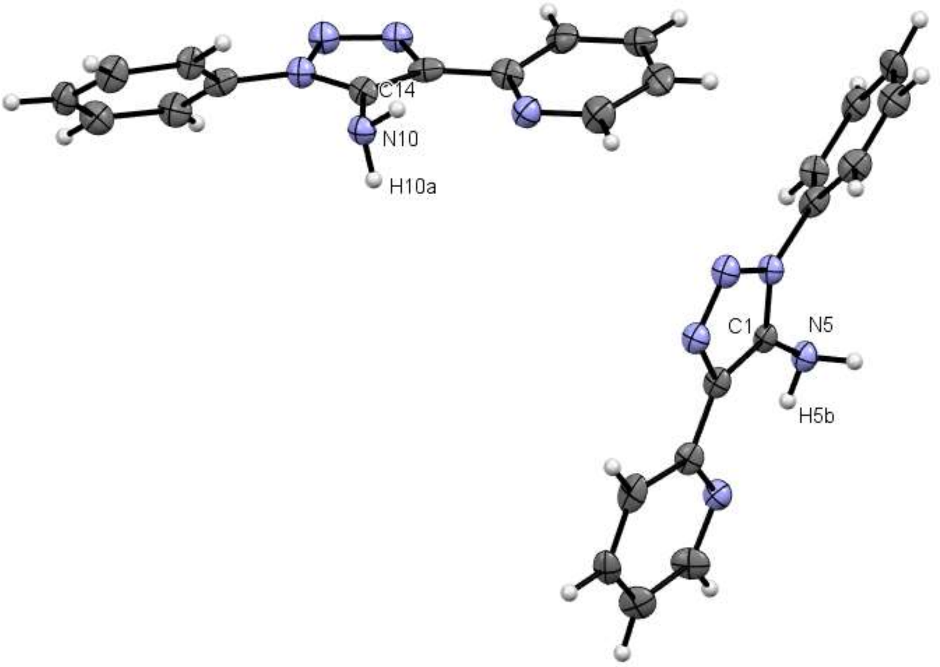


**Figure S67.** *X-ray solid-state structure of **1d**. Ellipsoids are all set at 50 % probability.*

| | | |
|---|---|---|
| Empirical formula | $C_{13}H_{11}N_5$ | |
| Formula weight | 237.266 | |
| CCDC Deposit Number | 2082294 | |
| Temperature | 140.01 K | |
| Wavelength | 0.71073 Å | |
| Crystal system | Monoclinic | |
| Space group | $P2_1$ | |
| Unit cell dimensions | a = 12.7186(13) Å | α = 90°. |
| | b = 6.2494(6) Å | β = 113.393(6)°. |
| | c = 15.5100(16) Å | γ = 90°. |
| Volume | 1131.5(2) $Å^3$ | |

| | |
|---|---|
| Z | 4 |
| Density (calculated) | 1.393 g/m$^3$ |
| Absorption coefficient | 0.090 mm$^{-1}$ |
| F(000) | 496 |
| Crystal size | 0.419 × 0.303 × 0.188 mm$^3$ |
| Theta range for data collection | 12.86 to 52.68°. |
| Index ranges | -15 ≤ h ≤ 14, -6 ≤ k ≤ 7, -19 ≤ l ≤ 19 |
| Reflections collected | 12808 |
| Independent reflections | 4147 [$R_{int}$ = 0.0467, $R_{sigma}$ = 0.0476] |
| Refinement method | Full-matrix least-squares on F$^2$ |
| Data / restraints / parameters | 4147 / 109 / 328 |
| Goodness-of-fit on F$^2$ | 1.042 |
| Final R indices [I≥2σ(I)] | $R_1$ = 0.1017, w$R_2$ = 0.2520 |
| R indices (all data) | $R_1$ = 0.1075, w$R_2$ = 0.2563 |
| Extinction coefficient | 0.012(2) |
| Largest diff. peak and hole | 0.45 and -0.67 e.Å$^{-3}$ |

Crystal data and structure refinement for **1d-O**

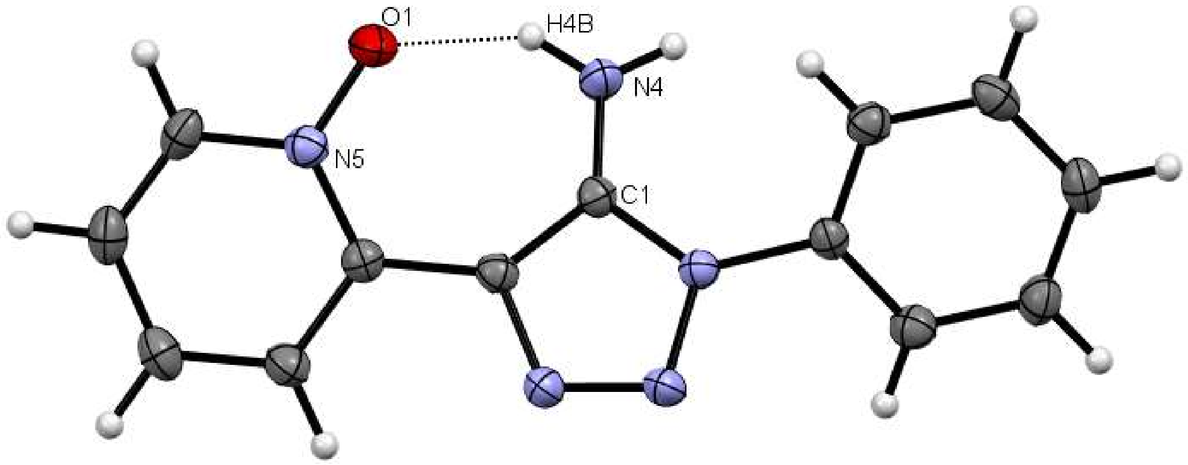


**Figure S68.** X-ray solid-state structure of **1d-O**. Ellipsoids are all set at 50 % probability. Selected bond parameters in [Å] and [°]. C1-N4 1.345(3), N5-O1 1.342(2), O1-H4B 2.087(2), N5-O1-H4B 110.0(1).

| | | |
|---|---|---|
| Empirical formula | $C_{13}H_{11}N_5O$ | |
| Formula weight | 253.27 | |
| CCDC Deposit Number | 2082105 | |
| Temperature | 140.01 K | |
| Wavelength | 0.71073 Å | |
| Crystal system | Orthorhombic | |
| Space group | $P2_12_12_1$ | |
| Unit cell dimensions | a = 8.5740(3) Å | α = 90°. |
| | b = 10.3820(3) Å | β = 90°. |
| | c = 12.9176(4) Å | γ = 90°. |
| Volume | 1149.86(6) Å$^3$ | |

| | |
|---|---|
| Z | 4 |
| Density (calculated) | 1.463 g/m$^3$ |
| Absorption coefficient | 0.100 mm$^{-1}$ |
| F(000) | 528 |
| Crystal size | 0.283 × 0.232 × 0.082 mm$^3$ |
| Theta range for data collection | 5.034 to 56.714°. |
| Index ranges | -11 ≤ h ≤ 11, -13 ≤ k ≤ 13, -16 ≤ l ≤ 17 |
| Reflections collected | 13138 |
| Independent reflections | 2866 [$R_{int}$ = 0.0332, $R_{sigma}$ = 0.0341] |
| Refinement method | Full-matrix least-squares on F$^2$ |
| Data / restraints / parameters | 2866 / 0 / 172 |
| Goodness-of-fit on F$^2$ | 1.046 |
| Final R indices [I≥2σ(I)] | $R_1$ = 0.0392, w$R_2$ = 0.0843 |
| R indices (all data) | $R_1$ = 0.0535, w$R_2$ = 0.0905 |
| Largest diff. peak and hole | 0.23 and -0.23 e.Å$^{-3}$ |

Crystal data and structure refinement for **2a**

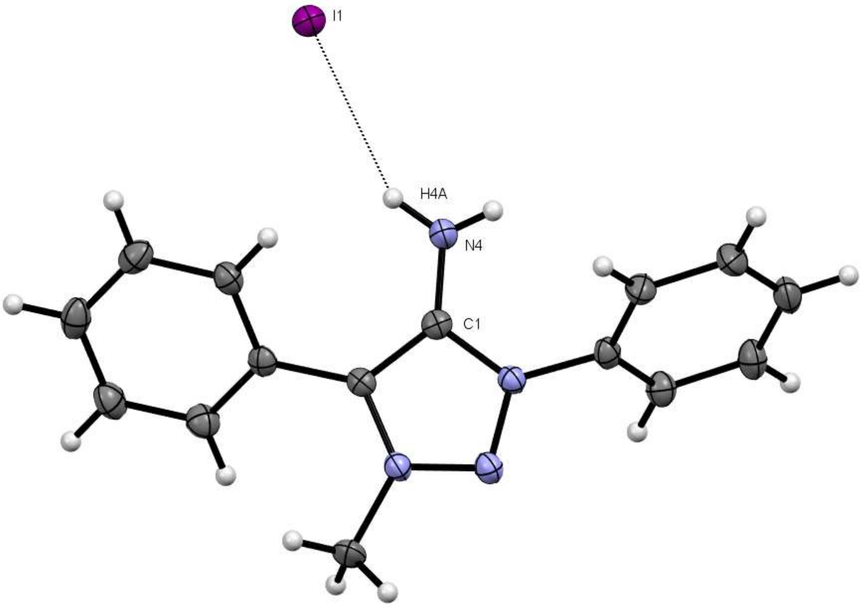


**Figure 69.** X-ray solid-state structure of **1d-O**. Ellipsoids are all set at 50 % probability. Selected bond parameters in [Å] and [°]. C1-N4 1.345(3), N5-O1 1.342(2), O1-H4B 2.087(2), N5-O1-H4B 110.0(1).

| | | |
|---|---|---|
| Empirical formula | $C_{15}H_{15}IN_4$ | |
| Formula weight | 378.21 | |
| CCDC Deposit Number | 2082308 | |
| Temperature | 293(2) K | |
| Wavelength | 0.71073 Å | |
| Crystal system | Monoclinic | |
| Space group | P2$_1$/c | |
| Unit cell dimensions | a = 10.6295(4) Å | α = 90°. |
| | b = 13.3791(4) Å | β = 109.305(3)°. |

| | | |
|---|---|---|
| | c = 11.4882(4) Å | γ = 90°. |
| Volume | 1541.91(10) $Å^3$ | |
| Z | 4 | |
| Density (calculated) | 1.629 $Mg/m^3$ | |
| Absorption coefficient | 2.073 $mm^{-1}$ | |
| F(000) | 744 | |
| Crystal size | n/a | |
| Theta range for data collection | 2.030 to 32.216°. | |
| Index ranges | -15 ≤ h ≤ 15, -19 ≤ k ≤ 19, -17 ≤ l ≤ 17 | |
| Reflections collected | 53467 | |
| Independent reflections | 5174 [R(int) = 0.0838] | |
| Completeness to theta = 25.242° | 100.0 % | |
| Refinement method | Full-matrix least-squares on $F^2$ | |
| Data / restraints / parameters | 5174 / 0 / 190 | |
| Goodness-of-fit on $F^2$ | 1.019 | |
| Final R indices [I>2σ(I)] | $R_1$ = 0.0435, $wR_2$ = 0.1023 | |
| R indices (all data) | $R_1$ = 0.0648, $wR_2$ = 0.1086 | |
| Extinction coefficient | n/a | |
| Largest diff. peak and hole | 1.305 and -1.307 e.$Å^{-3}$ | |

Crystal data and structure refinement for **2c**

| | | |
|---|---|---|
| Empirical formula | $C_{21}H_{27}IN_4$ | |
| Formula weight | 462.36 | |
| CCDC Deposit Number | 2085140 | |
| Temperature | 140.00 K | |
| Wavelength | 0.71073 Å | |
| Crystal system | Orthorhombic | |
| Space group | Pbca | |
| Unit cell dimensions | a = 16.1756(7) Å | α = 90°. |
| | b = 14.5456(5) Å | β = 90°. |
| | c = 18.0224(8) Å | γ = 90°. |
| Volume | 4240.4(3) $Å^3$ | |
| Z | 8 | |
| Density (calculated) | 1.449 $g/m^3$ | |
| Absorption coefficient | 1.522 $mm^{-1}$ | |
| F(000) | 1872.0 | |
| Crystal size | 0.328 × 0.189 × 0.074 $mm^3$ | |
| Theta range for data collection | 4.392 to 52.854°. | |
| Index ranges | -20 ≤ h ≤ 20, -17 ≤ k ≤ 18, -19 ≤ l ≤ 22 | |
| Reflections collected | 41190 | |
| Independent reflections | 4351 [$R_{int}$ = 0.0346, $R_{sigma}$ = 0.0252] | |

| | |
|---|---|
| Data / restraints / parameters | 4351 / 12 / 238 |
| Goodness-of-fit on $F^2$ | 1.138 |
| Final R indices [I≥2σ(I)] | $R_1$ = 0.0567, $wR_2$ = 0.1290 |
| R indices (all data) | $R_1$ = 0.0830, $wR_2$ = 0.1427 |
| Largest diff. peak and hole | 1.75 and -1.48 e.$Å^{-3}$ |

Crystal data and structure refinement for **3a**

| | | |
|---|---|---|
| Empirical formula | $C_{15}H_{14}N_4$ | |
| Formula weight | 250.30 | |
| CCDC Deposit Number | 2082090 | |
| Temperature | 140.0 K | |
| Wavelength | 0.71073 Å | |
| Crystal system | Triclinic | |
| Space group | P-1 | |
| Unit cell dimensions | a = 9.7738(6) Å | α = 102.721(3)°. |
| | b = 10.2222(7) Å | β = 99.497(3)°. |
| | c = 13.3134(10) Å | γ = 91.146(3)°. |
| Volume | 1277.51(15) $Å^3$ | |
| Z | 4 | |
| Density (calculated) | 1.301 $Mg/m^3$ | |
| Absorption coefficient | 0.081 $mm^{-1}$ | |
| F(000) | 528 | |
| Crystal size | 0.266 x 0.24 x 0.11 $mm^3$ | |
| Theta range for data collection | 1.592 to 28.349°. | |
| Index ranges | -12 ≤ h ≤ 13, -13 ≤ k ≤ 13, -17 ≤ l ≤ 17 | |
| Reflections collected | 27649 | |
| Independent reflections | 6300 [R(int) = 0.0202] | |
| Completeness to theta = 25.242° | 99.5 % | |
| Absorption correction | Semi-empirical from equivalents | |
| Max. and min. transmission | 0.7457 and 0.7090 | |
| Refinement method | Full-matrix least-squares on $F^2$ | |
| Data / restraints / parameters | 6300 / 0 / 455 | |
| Goodness-of-fit on $F^2$ | 1.031 | |
| Final R indices [I>2σ(I)] | $R^1$ = 0.0402, $wR^2$ = 0.0955 | |
| R indices (all data) | $R^1$ = 0.0512, $wR^2$ = 0.1017 | |
| Extinction coefficient | n/a | |
| Largest diff. peak and hole | 0.292 and -0.229 e.$Å^{-3}$ | |

Crystal data and structure refinement for the co-crystallised system **3b**-**2b**

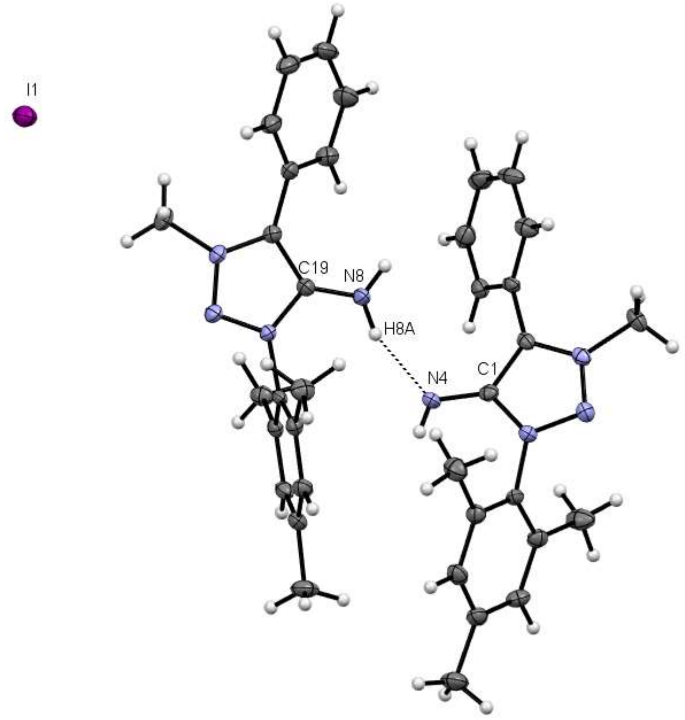


**Figure S70.** X-ray solid-state structure of the co-crystallised system **3b**-**2b**. Ellipsoids are all set at 50 % probability. Selected bond parameters in [Å] and [°]. C1-N4 1.310(3), N4-H8A 1.918(2), C1-N4-H8B 123.0(2), C19-N8 1.332(3).

| | | |
|---|---|---|
| Empirical formula | $C_{18}H_{20.5}I_{0.5}N_4$ | |
| Formula weight | 356.33 | |
| CCDC Deposit Number | 2082106 | |
| Temperature | 140.0 K | |
| Wavelength | 0.71073 Å | |
| Crystal system | Triclinic | |
| Space group | P-1 | |
| Unit cell dimensions | a = 11.3554(6) Å | α = 110.781(3)°. |
| | b = 11.9738(6) Å | β = 100.372(2)°. |
| | c = 13.5185(7) Å | γ = 95.304(2)°. |
| Volume | 1665.82(15) $Å^3$ | |
| Z | 4 | |
| Density (calculated) | 1.421 $g/m^3$ | |
| Absorption coefficient | 1.00 $mm^{-1}$ | |
| F(000) | 732.0 | |
| Crystal size | 0.286 × 0.263 × 0.12 $mm^3$ | |
| Theta range for data collection | 3.31 to 56.726°. | |
| Index ranges | -15 ≤ h ≤ 15, -15 ≤ k ≤ 15, -17 ≤ l ≤ 18 | |
| Reflections collected | 31570 | |
| Independent reflections | 8210 [$R_{int}$ = 0.0362, $R_{sigma}$ = 0.0492] | |
| Refinement method | Full-matrix least-squares on $F^2$ | |
| Data / restraints / parameters | 8210 / 0 / 417 | |
| Goodness-of-fit on $F^2$ | 1.041 | |

| | | |
|---|---|---|
| Final R indices [I≥2σ(I)] | $R_1$ = 0.0344, $wR_2$ = 0.0619 | |
| R indices (all data) | $R_1$ = 0.0561, $wR_2$ = 0.0685 | |
| Largest diff. peak and hole | 0.59 and -0.53 e.Å$^{-3}$ | |

Crystal data and structure refinement **3c**

| | | |
|---|---|---|
| Empirical formula | $C_{21}H_{26}N_4$ | |
| Formula weight | 334.46 | |
| CCDC Deposit Number | 2093977 | |
| Temperature | 139.99 K | |
| Wavelength | 0.71073 Å | |
| Crystal system | Triclinic | |
| Space group | P-1 | |
| Unit cell dimensions | a = 7.727(1) Å | α = 79.150(8)°. |
| | b = 8.394(2) Å | β = 87.771(8)°. |
| | c = 14.744(2) Å | γ = 82.346(9)°. |
| Volume | 930.7(3) Å$^3$ | |
| Z | 2 | |
| Density (calculated) | 1.193 g/m$^3$ | |
| Absorption coefficient | 0.072 mm$^{-1}$ | |
| F(000) | 360.0 | |
| Crystal size | 0.488 × 0.482 × 0.148 mm$^3$ | |
| Theta range for data collection | 2.812 to 52.774°. | |
| Index ranges | -8 ≤ h ≤ 9, -10 ≤ k ≤ 10, -12 ≤ l ≤ 18 | |
| Reflections collected | 14652 | |
| Independent reflections | 3802 [$R_{int}$ = 0.0345, $R_{sigma}$ = 0.0358] | |
| Data / restraints / parameters | 3802 / 0 / 237 | |
| Goodness-of-fit on $F^2$ | 1.076 | |
| Final R indices [I≥2σ(I)] | $R_1$ = 0.0631, $wR_2$ = 0.1754 | |
| R indices (all data) | $R_1$ = 0.0828, $wR_2$ = 0.1875 | |
| Largest diff. peak and hole | 0.34 and -0.35 e.Å$^{-3}$ | |

Crystal data and structure refinement for **3a-B($C_6F_5$)$_3$**

| | | |
|---|---|---|
| Empirical formula | $C_{33}H_{14}BF_{15}N_4$ | |
| Formula weight | 762.29 | |
| CCDC Deposit Number | 2082091 | |
| Temperature | 139.99 K | |
| Wavelength | 0.71073 Å | |
| Crystal system | Monoclinic | |
| Space group | $P2_1/c$ | |
| Unit cell dimensions | a = 17.4558(7) Å | α = 90°. |
| | b = 11.8190(5) Å | β = 115.868(3)°. |
| | c = 15.7702(6) Å | γ = 90°. |
| Volume | 2927.5(2) Å$^3$ | |
| Z | 4 | |
| Density (calculated) | 1.730 g/m$^3$ | |

| | |
|---|---|
| Absorption coefficient | 1.527 $mm^{-1}$ |
| F(000) | 1520.0 |
| Crystal size | 0.103 × 0.103 × 0.097$mm^3$ |
| Theta range for data collection | 5.626 to 125.99°. |
| Index ranges | -19 ≤ h ≤ 20, -13 ≤ k ≤ 13, -15 ≤ l ≤ 18 |
| Reflections collected | 15156 |
| Independent reflections | 4589 [$R_{int}$ = 0.0447, $R_{sigma}$ = 0.0435] |
| Refinement method | Full-matrix least-squares on $F^2$ |
| Data / restraints / parameters | 4589 / 0 / 479 |
| Goodness-of-fit on $F^2$ | 1.097 |
| Final R indices [I≥2σ(I)] | $R_1$ = 0.0669, $wR_2$ = 0.1679 |
| R indices (all data) | $R_1$ = 0.0922, $wR_2$ = 0.2031 |
| Largest diff. peak and hole | 0.98 and -0.38 e.$Å^{-3}$ |

Crystal data and structure refinement for **4**

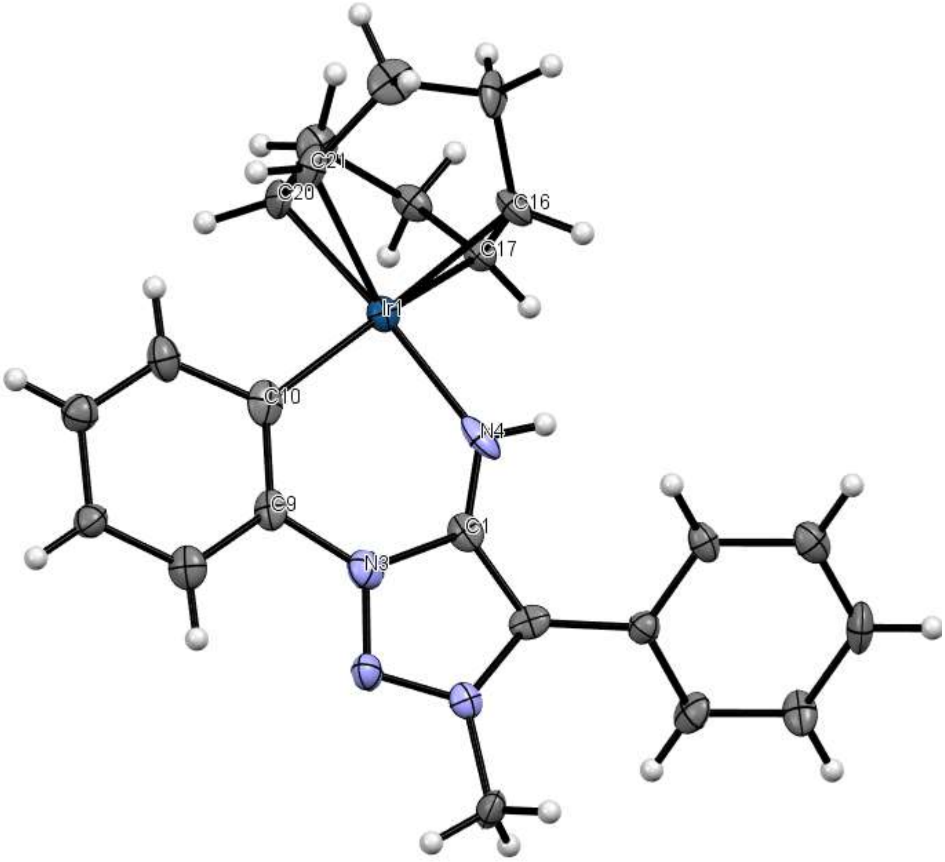


**Figure S71.** X-ray solid-state structure of **4**. Ellipsoids are all set at 50 % probability. Selected bond parameters in [Å] and [°]. C1-N4 1.29(2), N4-Ir1 2.02(2), C1-N4-Ir1 132(2), C10-N4-Ir1 87(1), C10-Ir1 2.08(2), C16/17-Ir1 2.06(1), C20/21-Ir1 2.01(1), C1-N3-C9-C10 4(2).

| | |
|---|---|
| Empirical formula | $C_{23}H_{25}IrN_4$ |
| Formula weight | 549.67 |
| CCDC Deposit Number | 208378 |
| Temperature | 293(3) K |
| Wavelength | 0.71073 Å |
| Crystal system | orthorhombic |
| Space group | $Pna2_1$ |

| | | |
|---|---|---|
| Unit cell dimensions | a = 7.3070(2) Å | α = 90°. |
| | b = 26.2885(10) Å | β = 90°. |
| | c = 10.1595(4) Å | γ = 90°. |
| Volume | 1951.54(12) $Å^3$ | |
| Z | 4 | |
| Density (calculated) | 1.871 $g/m^3$ | |
| Absorption coefficient | 6.858 $mm^{-1}$ | |
| F(000) | 1072.0 | |
| Crystal size | 1.0 × 0.3 × 0.2 $mm^3$ | |
| Theta range for data collection | 3.098 to 64.204°. | |
| Index ranges | -10 ≤ h ≤ 10, -38 ≤ k ≤ 37, -15 ≤ l ≤ 15 | |
| Reflections collected | 32541 | |
| Independent reflections | 6178 [$R_{int}$ = 0.0997, $R_{sigma}$ = 0.0983] | |
| Refinement method | Full-matrix least-squares on $F^2$ | |
| Data / restraints / parameters | 6178 / 25 / 243 | |
| Goodness-of-fit on $F^2$ | 1.022 | |
| Final R indices [I≥2σ(I)] | $R_1$ = 0.0532, $wR_2$ = 0.1121 | |
| R indices (all data) | $R_1$ = 0.1027, $wR_2$ = 0.1324 | |
| Largest diff. peak and hole | 3.82 and -2.18 e.$Å^{-3}$ | |

Crystal data and structure refinement for **5a**

| | | |
|---|---|---|
| Empirical formula | $C_{25}H_{28}IIrN_4$ | |
| Formula weight | 703.61 | |
| CCDC Deposit Number | 2082129 | |
| Temperature | 140.01 K | |
| Wavelength | 0.71073 Å | |
| Crystal system | triclinic | |
| Space group | P-1 | |
| Unit cell dimensions | a = 9.0988(17) Å | α = 84.348(8)° |
| | b = 11.2121(19) Å | β = 75.139(9)° |
| | c = 12.662(2) Å | γ = 87.321(8)° |
| Volume | 1242.1(4) $Å^3$ | |
| Z | 2 | |
| Density (calculated) | 1.881 $g/m^3$ | |
| Absorption coefficient | 6.636 $mm^{-1}$ | |
| F(000) | 672.0 | |
| Crystal size | 0.75 × 0.334 × 0.283 $mm^3$ | |
| Theta range for data collection | 3.342 to 66.484°. | |
| Index ranges | -13 ≤ h ≤ 14, -17 ≤ k ≤ 8, -19 ≤ l ≤ 19 | |
| Reflections collected | 40477 | |
| Independent reflections | 9418 [$R_{int}$ = 0.0295, $R_{sigma}$ = 0.0246] | |
| Refinement method | Full-matrix least-squares on $F^2$ | |
| Data / restraints / parameters | 9418 / 0 / 290 | |
| Goodness-of-fit on $F^2$ | 1.060 | |
| Final R indices [I≥2σ(I)] | $R_1$ = 0.0198, $wR_2$ = 0.0432 | |

| | | |
|---|---|---|
| R indices (all data) | $R_1$ = 0.0233, $wR_2$ = 0.0446 | |
| Largest diff. peak and hole | 1.18 and -1.39 e.Å$^{-3}$ | |

Crystal data and structure refinement for **5b**

| | | |
|---|---|---|
| Empirical formula | $C_{14.5}H_{18}Cl_{0.95}I_{0.5}Ir_{0.5}N_2$ | |
| Formula weight | 413.62 | |
| CCDC Deposit Number | 2082128 | |
| Temperature | 140.01 K | |
| Wavelength | 0.71073 Å | |
| Crystal system | triclinic | |
| Space group | P-1 | |
| Unit cell dimensions | a = 10.8224(5) Å | α = 82.449(2)°. |
| | b = 11.3562(5) Å | β = 70.549(2)°. |
| | c = 14.2993(6) Å | γ = 84.804(2)°. |
| Volume | 1640.67(13) Å$^3$ | |
| Z | 4 | |
| Density (calculated) | 1.675 g/m$^3$ | |
| Absorption coefficient | 5.188 mm$^{-1}$ | |
| F(000) | 801.0 | |
| Crystal size | 0.686 × 0.388 × 0.371 mm$^3$ | |
| Theta range for data collection | 3.622 to 66.572°. | |
| Index ranges | -16 ≤ h ≤ 16, -17 ≤ k ≤ 17, -22 ≤ l ≤ 22 | |
| Reflections collected | 53931 | |
| Independent reflections | 12500 [$R_{int}$ = 0.0303, $R_{sigma}$ = 0.0261] | |
| Refinement method | Full-matrix least-squares on F$^2$ | |
| Data / restraints / parameters | 12500 / 0 / 348 | |
| Goodness-of-fit on F$^2$ | 1.056 | |
| Final R indices [I≥2σ(I)] | $R_1$ = 0.0243, $wR_2$ = 0.0576 | |
| R indices (all data) | $R_1$ = 0.0308, $wR_2$ = 0.0609 | |
| Largest diff. peak and hole | 2.29 and -1.85 e.Å$^{-3}$ | |

Crystal data and structure refinement for **5d**

| | | |
|---|---|---|
| Empirical formula | $C_{29}H_{33}F_6IrN_6O_6S_2$ | |
| Formula weight | 931.93 | |
| CCDC Deposit Number | 2082093 | |
| Temperature | 139.99 K | |
| Wavelength | 0.71073 Å | |
| Crystal system | Monoclinic | |
| Space group | C2/c | |
| Unit cell dimensions | a = 35.8233(15) Å | α = 90°. |
| | b = 18.3547(4) Å | β = 90.215(4)°. |
| | c = 23.0390(9) Å | γ = 90°. |
| Volume | 6895.4(5) Å$^3$ | |
| Z | 8 | |

| | |
|---|---|
| Density (calculated) | 1.798 g/m$^3$ |
| Absorption coefficient | 4.077 mm$^{-1}$ |
| F(000) | 3680.0 |
| Crystal size | 0.204 × 0.131 × 0.12 mm$^3$ |
| Theta range for data collection | 3.536 to 61.168°. |
| Index ranges | -50 ≤ h ≤ 51, -11 ≤ k ≤ 11, -32 ≤ l ≤ 32 |
| Reflections collected | 109215 |
| Independent reflections | 10570 [$R_{int}$ = 0.0484, $R_{sigma}$ = 0.0315] |
| Refinement method | Full-matrix least-squares on F$^2$ |
| Data / restraints / parameters | 10570 / 324 / 502 |
| Goodness-of-fit on F$^2$ | 1.038 |
| Final R indices [I≥2σ(I)] | $R_1$ = 0.0266, $wR_2$ = 0.0507 |
| R indices (all data) | $R_1$ = 0.0434, $wR_2$ = 0.0553 |
| Largest diff. peak and hole | 0.95 and -0.67 e.Å$^{-3}$ |

Crystal data and structure refinement for **5d-acetone**

| | | |
|---|---|---|
| Empirical formula | $C_{27}H_{30}F_6IrN_5O_6S_2$ | |
| Formula weight | 890.88 | |
| CCDC Deposit Number | 2114162 | |
| Temperature | 139.99 K | |
| Wavelength | 0.71073 Å | |
| Crystal system | Triclinic | |
| Space group | P-1 | |
| Unit cell dimensions | a = 10.7638(8) Å | α = 109.485(3°. |
| | b = 10.9663(7) Å | β = 92.013(5)°. |
| | c = 14.220(1) Å | γ = 100.650(4)°. |
| Volume | 1546.7(2) Å$^3$ | |
| Z | 2 | |
| Density (calculated) | 1.913 g/m$^3$ | |
| Absorption coefficient | 4.538 mm$^{-1}$ | |
| F(000) | 876.0 | |
| Crystal size | 0.242 × 0.155 × 0.065 mm$^3$ | |
| Theta range for data collection | 3.054 to 52.974°. | |
| Index ranges | -13 ≤ h ≤ 13, -13 ≤ k ≤ 13, -16 ≤ l ≤ 17 | |
| Reflections collected | 27169 | |
| Independent reflections | 6336 [$R_{int}$ = 0.0675, $R_{sigma}$ = 0.0774] | |
| Data / restraints / parameters | 6336 / 282 / 435 | |
| Goodness-of-fit on F$^2$ | 1.059 | |
| Final R indices [I≥2σ(I)] | $R_1$ = 0.0573, $wR_2$ = 0.1574 | |
| R indices (all data) | $R_1$ = 0.0804, $wR_2$ = 0.1754 | |
| Largest diff. peak and hole | 3.42 and -2.42 e.Å$^{-3}$ | |

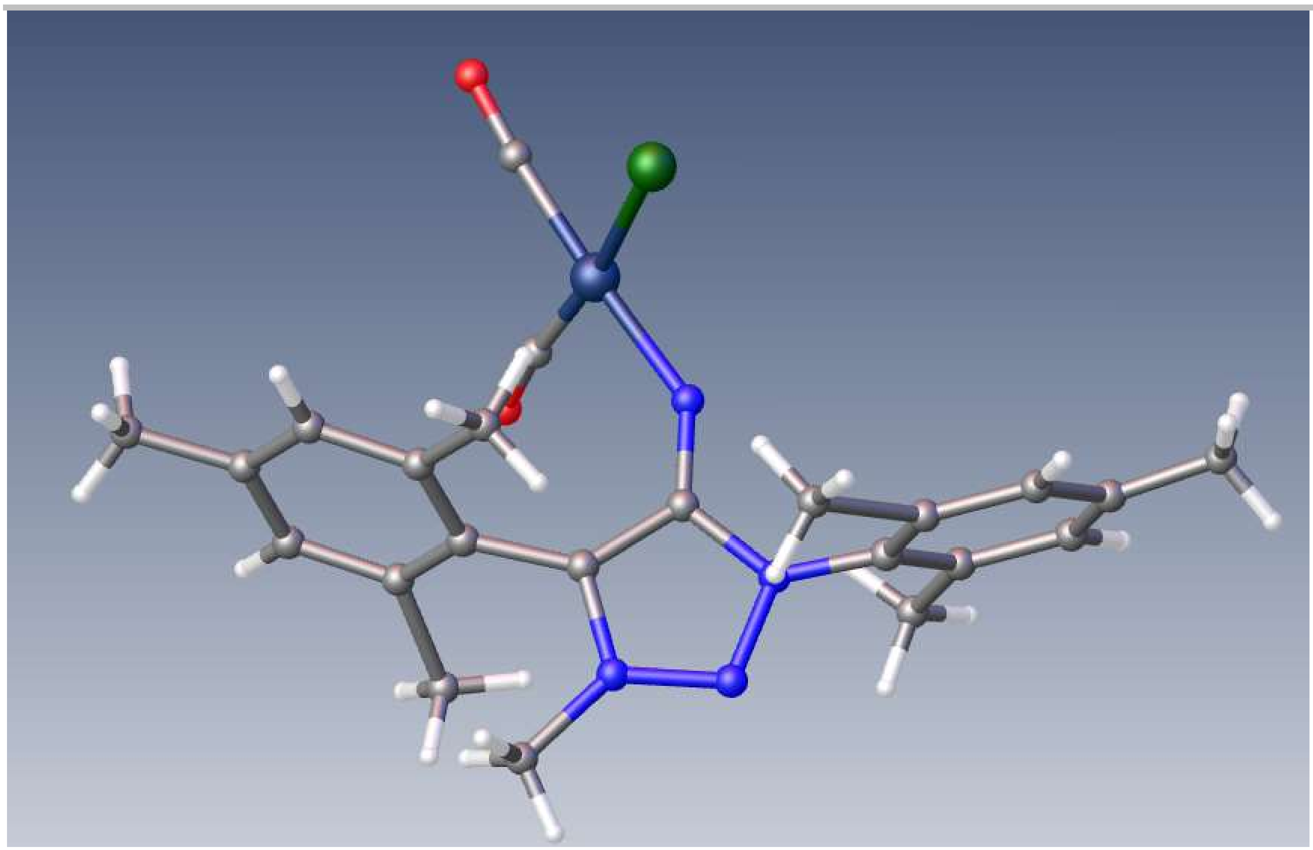

**Figure S72.** X-ray solid-state structure **3c-Rh(CO)$_2$Cl**.

## References


[1] J. T. Simmons, J. R. Allen, D. R. Morris, R. J. Clark, C. W. Levenson, M. W. Davidson, L. Zhu, *Inorg. Chem.* **2013**, *52*, 5838.
[2] F. Kloss, U. Köhn, B. O. Jahn, M. D. Hager, H. Görls, U. S. Schubert, *Chem. Asian J.* **2011**, *6*, 2816.
[3] N. Kore, P. Pazdera, *Curr. Org. Synth.* **2018**, *15*, 552.
[4] G. L'Abbé, K. Vercauteren, W. Dehaen, *Bull. Soc. Chim. Belg.* **1994**, *103*, 321.
[5] G. R. Fulmer, A. J. M. Miller, N. H. Sherden, H. E. Gottlieb, A. Nudelman, B. M. Stoltz, J. E. Bercaw, K. I. Goldberg, *Organometallics* **2010**, *29*, 2176.
[6] a) R. K. Harris, E. D. Becker, Cabral de Menezes, Sonia M., R. Goodfellow, P. Granger, *Solid State Nucl. Magn. Reson.* **2002**, *22*, 458; b) R. K. Harris, E. D. Becker, S. M. C. de Menezes, R. Goodfellow, P. Granger, *Magn. Reson. Chem.* **2002**, *40*, 489.
[7] a) G. M. Sheldrick, *Acta Crystallogr. A* **2008**, *64*, 112; b) G. M. Sheldrick, *Acta Crystallogr. C* **2015**, *71*, 3; c) G. M. Sheldrick, *Acta Crystallogr. A* **2015**, *71*, 3.
[8] A. Bolje, J. Košmrlj, *Org. Lett.* **2013**, *15*, 5084.
[9] P. Mathew, A. Neels, M. Albrecht, *J. Am. Chem. Soc.* **2008**, *130*, 13534.
[10] J. M. Blackwell, W. E. Piers, M. Parvez, R. McDonald, *Organometallics* **2002**, *21*, 1400.
[11] a) F. Neese, *Wiley Interdiscip. Rev.: Comput. Mol. Sci.* **2012**, *2*, 73; b) F. Neese, *Wiley Interdiscip. Rev.: Comput. Mol. Sci.* **2018**, *8*, e1327.
[12] C. Adamo, V. Barone, *J. Chem. Phys.* **1999**, *110*, 6158.
[13] F. Weigend, R. Ahlrichs, *PCCP* **2005**, *7*, 3297.
[14] A. Kundu, S. Chandra, D. Mandal, N. I. Neuman, A. Mahata, S. Anga, H. Rawat, S. Pal, C. Schulzke, B. Sarkar et al., *J. Org. Chem.* **2021**, *86*, 12683.
[15] A. V. Marenich, C. J. Cramer, D. G. Truhlar, *J. Phys. Chem. B* **2009**, *113*, 6378.
[16] V. Barone, M. Cossi, *J. Phys. Chem. A* **1998**, *102*, 1995.
[17] a) T. Petrenko, S. Kossmann, F. Neese, *J. Chem. Phys.* **2011**, *134*, 54116; b) F. Neese, G. Olbrich, *Chem. Phys. Lett.* **2002**, *362*, 170; c) R. Izsák, F. Neese, *J. Chem. Phys.* **2011**, *135*, 144105; d) J. L. Whitten, *J. Chem. Phys.* **1973**, *58*, 4496; e) O. Vahtras, J. Almlöf, M.W. Feyereisen, *Chem. Phys. Lett.* **1993**, *213*, 514; f) F. Neese, F. Wennmohs, A. Hansen, U. Becker, *Chem. Phys.* **2009**, *356*, 98.
[18] a) K. Eichkorn, O. Treutler, H. Öhm, M. Häser, R. Ahlrichs, *Chem. Phys. Lett.* **1995**, *242*, 652; b) K. Eichkorn, F. Weigend, O. Treutler, R. Ahlrichs, *Theor. Chem. Acc.* **1997**, *97*, 119; c) F. Weigend, *PCCP* **2006**, *8*, 1057.
[19] Chemcraft - graphical software for visualization of quantum chemistry computations. https://www.chemcraftprog.com.
